# Association of Bevacizumab Plus Oxaliplatin-Based Chemotherapy With Disease-Free Survival and Overall Survival in Patients With Stage II Colon Cancer: A Secondary Analysis of the AVANT Trial

**DOI:** 10.1001/jamanetworkopen.2020.20425

**Published:** 2020-10-19

**Authors:** Benoist Chibaudel, Julie Henriques, Manel Rakez, Baruch Brenner, Tae Won Kim, Mercedes Martinez-Villacampa, Javier Gallego-Plazas, Andres Cervantes, Katharine Shim, Derek Jonker, Veronique Guerin-Meyer, Laurent Mineur, Chiara Banzi, Alice Dewdney, Thitiya Dejthevaporn, Haiko J. Bloemendal, Arnaud Roth, Markus Moehler, Enrique Aranda, Eric Van Cutsem, Josep Tabernero, Hans-Joachim Schmoll, Paulo M. Hoff, Thierry André, Aimery de Gramont

**Affiliations:** 1 Department of Medical Oncology, Franco-British Hospital–Fondation Cognacq-Jay, Levallois-Perret, France; 2 Statistical Unit, Aide et Recherche en Cancérologie Digestive, Foundation, Levallois-Perret, France; 3 Methodology and Quality of Life Unit in Oncology, University Hospital of Besançon, Institut National de la Santé et de la Recherche Médicale, Unité Mixte de Recherche 1098, Besançon, France; 4 Institute of Oncology, Davidoff Cancer Center, Rabin Medical Center, Petah Tiqva, Tel Aviv University, Tel Aviv, Israel; 5 Department of Oncology, Asan Medical Center, University of Ulsan College of Medicine, Seoul, Korea; 6 Department of Medical Oncology, Institut Català d'Oncologia-Bellvitge Institute for Biomedical Research, L’Hospitalet, Barcelona, Spain; 7 Department of Medical Oncology, General Universitario de Elche Hospital, Elche, Spain; 8 Department of Medical Oncology, Hospital Clinico Universitario de Valencia, Valencia, Spain; 9 Department of Medical Oncology, Lakeridge Health R.S. McLaughlin Durham Regional Cancer Centre, Oshawa, Ontario, Canada; 10 Division of Medical Oncology, Department of Medicine, Ottawa Hospital Research Institute, University of Ottawa, Ottawa, Canada; 11 Department of Gastroenterology and Hepatology, Institut de Cancérologie de l’Ouest Paul Papin, Angers, France; 12 Department of Radiotherapy and Oncology Gastrointestinal and Liver, Institut Sainte Catherine, Avignon, France; 13 Medical Oncology Unit, Arcispedale Santa Maria Nuova-Istituto di Ricovero e Cura a Carattere Scientifico, Reggio Emilia, Italy; 14 Department of Oncology, Weston Park Hospital Cancer Research Centre, Sheffield, United Kingdom; 15 Medical Oncology Unit, Ramathibodi Hospital, Bangkok, Thailand; 16 Department of Internal Medicine and Medical Oncology, Radboud University Medical Center, Nijmegen, Netherlands; 17 Digestive Tumor Unit, Department of Oncology, Hôpitaux Universitaires de Genève, Geneva, Switzerland; 18 First Department of Internal Medicine, University Hospital of Mainz, Mainz, Germany; 19 Department of Medical Oncology, Reina Sofía University Hospital, Córdoba, Spain; 20 Department of Gastroenterology and Digestive Oncology, University Hospitals Gasthuisberg/Leuven and KULeuven, Leuven, Belgium; 21 Department of Medical Oncology, Vall d’Hebron University Hospital and Vall d’Hebron Institute of Oncology, Universitat de Vic–Universitat Central de Catalunya, International Oncology Bureau–Quiron, Barcelona, Spain; 22 Department of Oncology and Hematology, Martin Luther University, Halle, Germany; 23 Department of Radiology and Oncology, Instituto de Câncer do Estado de São Paulo, São Paulo, Brazil; 24 Department of Medical Oncology, Hôpital Saint-Antoine, Assitance Publique des Hôpitaux de Paris, Paris, France

## Abstract

**Importance:**

In the pivotal Bevacizumab-Avastin Adjuvant (AVANT) trial, patients with high-risk stage II colon cancer (CC) had 5-year and 10-year overall survival (OS) rates of 88% and 75%, respectively, with adjuvant fluorouracil and oxaliplatin–based chemotherapy; however, the trial did not demonstrate a disease-free survival (DFS) benefit of adding bevacizumab to oxaliplatin-based chemotherapy in stage III CC and suggested a detrimental effect on OS. The Long-term Survival AVANT (S-AVANT) study was designed to collect extended follow-up for patients in the AVANT trial.

**Objective:**

To explore the efficacy of adjuvant bevacizumab combined with oxaliplatin-based chemotherapy in patients with high-risk, stage II CC.

**Design, Setting, and Participants:**

This prespecified secondary end point analysis of the AVANT and S-AVANT studies included 573 patients with curatively resected high-risk stage II CC and at least 1 of the following criteria: stage T4, bowel obstruction or perforation, blood and/or lymphatic vascular invasion and/or perineural invasion, age younger than 50 years, or fewer than 12 nodes analyzed. The AVANT study was a multicenter randomized stage 3 clinical trial. Data were collected from December 2004 to February 2019, and data for this study were analyzed from March to September 2019.

**Intervention:**

Patients were randomly assigned to receive 5-fluorouracil, leucovorin, and oxaliplatin (FOLFOX4), FOLFOX4 with bevacizumab, or capecitabine and oxaliplatin (XELOX) with bevacizumab.

**Main Outcomes and Measures:**

The primary end points of this secondary analysis were DFS and OS in patients with high-risk stage II CC.

**Results:**

The AVANT study included 3451 patients, of whom 573 (16.6%) had high-risk stage II CC (192 [33.5%] randomized to FOLFOX4 group; 194 [33.9%] randomized to FOLFOX4 with bevacizumab group; 187 [32.6%] randomized to XELOX with bevacizumab group). With a median (interquartile range) age of 57.0 (47.2-65.7) years, the study population comprised 325 men (56.7%) and 248 women (43.3%). After a median (interquartile range) follow-up of 6.9 (6.1-11.3) years, the 3-year DFS and 5-year OS rates were 88.2% (95% CI, 83.7%-93.0%) and 89.7% (95% CI, 85.4%-94.2%) in the FOLFOX4 group, 86.6% (95% CI, 81.8%-91.6%) and 89.7% (95% CI, 85.4%-94.2%) in the FOLFOX4 with bevacizumab group, and 86.7% (95% CI, 81.8%-91.8%) and 93.2% (95% CI, 89.6%-97.0%) in the XELOX with bevacizumab group, respectively. The DFS hazard ratio was 0.94 (95% CI, 0.59-1.48; *P* = .78) for FOLFOX4 with bevacizumab vs FOLFOX4 and 1.07 (95% CI, 0.69-1.67; *P* = .76) for XELOX with bevacizumab vs FOLFOX4. The OS hazard ratio was 0.92 (95% CI, 0.55-1.55; *P* = .76) for FOLFOX4 with bevacizumab vs FOLFOX4 and 0.85 (95% CI, 0.50-1.44; *P* = .55) for XELOX with bevacizumab vs FOLFOX4.

**Conclusions and Relevance:**

In this secondary analysis of data from the AVANT trial, adding bevacizumab to oxaliplatin-based chemotherapy was not associated with longer DFS or OS in patients with high-risk stage II CC. The findings suggest that the definition of high-risk stage II CC needs to be revisited.

**Trial Registration:**

ClinicalTrial.gov Identifiers: AVANT (NCT00112918); S-AVANT (NCT02228668)

## Key Points

**Question:**

What was the efficacy of adjuvant bevacizumab on disease-free and overall survival among patients with curatively resected high-risk stage II colon cancer?

**Findings:**

In this secondary analysis of 573 patients with high-risk stage II colon cancer enrolled in the AVANT trial, the addition of bevacizumab to oxaliplatin-based chemotherapy did not prolong disease-free survival or overall survival. The 5-year survival rates in patients with high-risk stage II colon cancer in this study were 89.7% in the FOLFOX4 control group and in the FOLFOX4 with bevacizumab group and 93.2% in the XELOX with bevacizumab group.

**Meaning:**

The findings of this study suggest that adding bevacizumab to oxaliplatin-based chemotherapy was not associated with longer DFS or OS in patients with high-risk stage II colon cancer and that the definition of high-risk stage II colon cancer needs to be revisited.

## Teaser

This secondary analysis of the AVANT trial explores the efficacy of adjuvant bevacizumab combined with oxaliplatin-based chemotherapy and disease-free survival (DFS) rates in patients with high-risk, stage II colon cancer.

## Introduction

Colorectal cancer (CRC) is the fourth most common cancer in the world and the fifth leading cause of death.^1^ Nearly 25% of colon cancers (CCs) are diagnosed with stage II disease in western countries.^2^ In the pivotal Multicenter International Study of Oxaliplatin/Fluorouracil/Leucovorin in the Adjuvant Treatment of Colon Cancer (MOSAIC) trial, which assessed the addition of oxaliplatin to fluorouracil, patients with high-risk stage II CC had 5-year and 10-year overall survival (OS) rates of 88% and 75%, respectively, with adjuvant folinic acid, fluorouracil, oxaliplatin (FOLFOX) chemotherapy.^3,4^ However, the use of adjuvant therapy for patients with stage II CC remains controversial.

The survival benefit of 6-month adjuvant therapy with bolus 5-fluorouracil/leucovorin (5-FU/LV) in patients with resected, node-positive CC (stage III) was established in the 1990s.^5,6,7^ It was later superseded in trials by infusional 5-FU/LV regimens, which showed an improved safety profile.^8^ The efficacy of 5-FU/LV in adjuvant therapy for stage II CC was established by the Quick and Simple and Reliable (QUASAR) study.^9^ In the population with stage II disease, this study showed that adjuvant chemotherapy decreased the relative risk of recurrence by 29% (hazard ratio [HR], 0.71; 95% CI, 0.54-0.92), but there was no difference in OS (HR, 0.83; 95% CI, 0.65-1.07). However, the absolute benefit of adjuvant fluoropyrimidine was only 2.9%, leading to an absence of therapeutic consensus.^9^ Further changes to adjuvant treatment have since been made by the addition of oxaliplatin to fluoropyrimidines (5-FU/LV or capecitabine), reducing the relative risk of recurrence by 20% to 23% in patients with stage III CC.^3,10,11^ In the MOSAIC study, oxaliplatin added to 5-FU/LV had some activity in patients with high-risk stage II CC. The 5-year disease-free survival (DFS) rate for these patients was 82.3% (vs 74.6% for those receiving only 5-FU/LV; HR, 0.72; 95% CI, 0.50-1.02). However, OS was not increased.^12^ Considering these results, adjuvant treatment for stage II CC is discussed according to tumor-related prognostic factors and should be balanced with patient’s comorbidity and life expectancy.

Vascular endothelial growth factor inhibition with bevacizumab, a humanized anti–vascular endothelial growth factor monoclonal antibody, has a direct antivascular effect in human tumors, improving OS when given with chemotherapy in patients with metastatic CRC.^13,14^ The Bevacizumab-Avastin Adjuvant (AVANT) trial, a 3-group multinational phase 3 study, failed to demonstrate the superiority of bevacizumab with 5-fluorouracil, leucovorin, and oxaliplatin (FOLFOX4) or capecitabine and oxaliplatin (XELOX) compared with FOLFOX4 alone in terms of DFS in patients who had undergone surgery with curative intent for stage III CC.^15^

The National Surgical Adjuvant Breast and Bowel Project C-08 phase 3 trial also investigated the efficacy of bevacizumab plus adjuvant oxaliplatin-based chemotherapy in US patients with stage II or III CC.^16^ Similarly, adding bevacizumab did not increase DFS. In that study,^16^ the effect of bevacizumab treatment did not vary by stage. QUASAR2 evaluated adjuvant capecitabine plus bevacizumab vs capecitabine alone in patients with high-risk stage II or stage III CRC. The addition of bevacizumab to capecitabine yielded no DFS benefit (HR, 1.06; 95% CI, 0.89-1.25).^17^ In the present study, we report the updated results of bevacizumab as adjuvant therapy combined with oxaliplatin-based chemotherapy in the exploratory cohort of patients with high-risk stage II CC, who were included in the AVANT trial.

## Methods

### Study Design and Treatment

The AVANT trial was a prospective, multicenter, randomized, parallel, 3-group phase 3 trial conducted from December 2004 to June 2007. The primary objective was to evaluate the addition of bevacizumab to a standard oxaliplatin-based chemotherapy regimen in patients with stage III CC. Patients with high-risk stage II CC were also eligible for secondary objectives. A total of 2867 patients with stage III CC and 573 patients with stage II were randomized. Full details of the trial design were published in 2012.^15^

Eligible patients were aged 18 years or older and had an Eastern Cooperative Oncology Group (ECOG) performance status of 0 or 1 with histologically confirmed stage III or high-risk stage II CC (defined by the American Joint Cancer Committee/Union for International Cancer Control). Patients with stage II CC were considered high-risk if they fulfilled at least 1 of the following criteria: younger than 50 years, stage T4, perforation or obstruction, fewer than 12 lymph nodes examined, or vascular (ie, blood and lymphatic vessels) or perineural invasion. Curative surgery was performed 4 to 8 weeks before randomization.

Key exclusion criteria included remaining tumor, carcinoembryonic antigen level greater than 1.5 times the upper reference limit after surgery, previous antiangiogenic treatment, major surgical procedure, open biopsy, significant traumatic injury fewer than 28 days before study treatment start, and abnormal hematological, liver, or renal function.

The study was done in accordance with the Declaration of Helsinki,^18^ and the protocol (Supplement 1) was approved by the ethics review committees or boards of the institutions involved in this multinational study. Patients provided written informed consent. This study follows the Consolidated Standards of Reporting Trials (CONSORT) reporting guideline.

Randomization was performed after surgery using a centralized interactive computerized system and stratified according to geographic region (n = 8) and disease stage. The study had an open-label design.

Patients were randomly assigned in a 1:1:1 ratio to 1 of 3 treatment options: FOLFOX4 for 24 weeks followed by observation for 24 weeks; FOLFOX4 with bevacizumab for 24 weeks followed by bevacizumab monotherapy for 24 weeks, or XELOX with bevacizumab for 24 weeks followed by bevacizumab monotherapy for 24 weeks. FOLFOX4 and XELOX were given as described previously.^3,10^ Bevacizumab was administered as a 30-minute to 90-minute intravenous infusion on day 1 before oxaliplatin (5 mg/kg every 2 weeks for FOLFOX4; 7.5 mg/kg every 3 weeks for XELOX). Bevacizumab monotherapy was given at 7.5 mg/kg every 3 weeks.

### Study End Points

The primary end point of the AVANT study was the superiority of bevacizumab with FOLFOX4 or XELOX vs FOLFOX4 alone in terms of DFS in patients with stage III CC only. DFS was defined as the time between randomization and recurrence, new occurrence of CRC, or death from any cause, whichever occurred first. Patients who were event free at the clinical cutoff date were censored at the last date at which they were known to be disease free. Recurrences and new occurrences were based on an investigator tumor assessment, prescheduled every 6 months for 4 years following randomization and scheduled annually thereafter. OS was defined as the time from randomization to death. Patients who were still alive at the clinical cutoff date were censored at the date at which they were last confirmed to be alive. Survival status was assessed every 6 months for the first 4 years after randomization and annually thereafter. AVANT was an event and/or time-driven trial. The study continued until 36 months after the last patient was randomized, and results were mature for analysis in 2010 for the study primary objective.

Adverse events were monitored starting 28 days after the last dose of study treatment and/or the end of the observation phase and were graded according to National Cancer Institute Common Toxicity Criteria for Adverse Events version 3.0. Safety was previously reported in the pooled population of patients with stage II and stage III CC in the AVANT trial.^15^

### Follow-up and Update

Follow-up was initially done by the sponsor Roche with a data lock on June 30, 2010, and a median (range) follow-up of 48 (0-66) months. At that time, DFS but not OS was mature. Given the negative results, Roche decided to close the trial. Consequently, following the investigators’ wish to obtain mature survival data, the database was transferred to Gercor in 2012. The S-AVANT study was designed to obtain the ultimate DFS and OS results with extended follow-up of patients previously randomized in the AVANT trial and to provide estimates of long-term effects of bevacizumab added to adjuvant chemotherapy in patients with stage II and stage III CC. Further analyses were performed using updated individual patient data and focusing on DFS, OS, comorbidities observed during the follow-up period, and reasons of deaths. Adverse events of special interest to bevacizumab and their related serious adverse events were also collected. Long-term survival results in patients with stage III CC were published previously.^19^ We report here the survival results of patients with high-risk stage II CC.

### Statistical Analysis

All patients with high-risk stage II CC who were randomized in the AVANT study were eligible for exploratory analyses. DFS and OS were the primary end points of this prespecified secondary analysis. Continuous and categorical variables were described using the median value with interquartile range (IQR) and frequency with percentage, respectively. DFS and OS were estimated using the Kaplan-Meier method, and the log-rank test was used to assess for treatment differences overall. Follow-up was calculated using the reverse Kaplan-Meier estimation. Cox proportional hazard models were performed to estimate the association of factors with DFS and OS by means of HRs and 95% CIs. To assess the association between DFS and OS and the baseline parameters, univariate Cox analyses were first carried out. Factors with *P* < .10 were then added to the final multivariate Cox model, after consideration of collinearity among variables. The proportional hazard assumption was checked using statistical tests and graphical diagnostics based on the scaled Schoenfeld residuals. Treatment group associations (FOLFOX4 with bevacizumab vs FOLFOX4 alone, and XELOX with bevacizumab vs FOLFOX4 alone) with DFS and OS were assessed using subgroup analyses, and results were summarized in forest plots. An interaction term in each subgroup was obtained by considering the subgroup, the treatment group, and their interaction in a Cox model. An exploratory analysis of OS according to the number of stage II high-risk factors was performed. Considering that patients with high-risk stage II CC were recruited for exploratory analyses and that their total number was restricted to approximately 16% of all patients, no formal statistical hypotheses were formulated for this prespecified study analysis. *P* values are shown for exploratory purposes and were not adjusted for multiple testing. All tests were 2-sided. All analyses were performed using R version 3.6.3 software (R Project for Statistical Computing) from March to September 2019.

## Results

### Patient Characteristics

**Figure 1.**
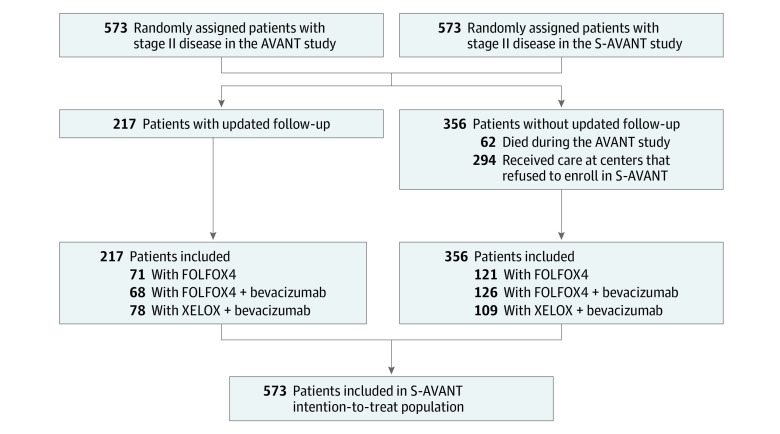
Flowchart of Participants in Bevacizumab-Avastin Adjuvant (AVANT) and Long-term Survival Bevacizumab-Avastin Adjuvant (S-AVANT) Studies FOLFOX4 indicates 5-fluorouracil, leucovorin, and oxaliplatin; and XELOX, capecitabine and oxaliplatin.

The AVANT study included 3451 patients, of whom 573 (16.6%) had stage II CC (192 [33.5%] in FOLFOX4 group; 194 [33.9%] in FOLFOX4 with bevacizumab group; 187 [32.6%] in XELOX with bevacizumab group) (Figure 1). With a median (IQR) age of 57.0 (47.2-65.7) years, the study population comprised 325 men (56.7%) and 248 women (43.3%). Patient characteristics and high-risk stage II CC criteria are presented in Table 1 and eFigure 1 in Supplement 2. The most frequent high-risk stage II criterion was fewer than 12 examined lymph nodes (243 patients [42.4%]). Of note, 318 patients (55.5%) had a single high-risk stage II criterion, and 255 patients (44.5%) had more than 1. When data were locked for analysis on February 15, 2019, the median (IQR) follow-up for the whole population of high-risk stage II CC was 6.9 (6.1-11.3) years, with 6.9 (6.1-11.3) years in the FOLFOX4 group, 6.8 (6.2-11.3) years in the FOLFOX4 with bevacizumab group, and 6.9 (6.1-11.5) years in the XELOX with bevacizumab group. Of 511 patients (89.2%) with high-risk stage II CC who were still alive after the AVANT study, 217 (42.5%) had an updated median (IQR) follow-up of 11.6 (10.8-12.6) years.

**Table 1.** Clinical Characteristics of Patients With Stage II Colon Cancer

Characteristic	Patients, No. (%)			
	All (N = 573)	FOLFOX4 group (n = 192)	FOLFOX4 with bevacizumab group (n = 194)	XELOX with bevacizumab group (n = 187)
Sex^a^				
Men	325 (56.7)	124 (64.6)	99 (51.0)	102 (54.5)
Women	248 (43.3)	68 (35.4)	95 (49.0)	85 (45.4)
Age, y^a^				
Median (IQR)	57.0 (47.2-65.7)	57.2 (48.2-66.9)	57.1 (46.0-66.2)	56.1 (47.4-64.3)
<50	191 (33.3)	62 (32.3)	70 (36.1)	59 (31.5)
≥50	382 (66.7)	130 (67.7)	124 (63.9)	128 (68.4)
Primary tumor				
Left or rectum	343 (61.9)	116 (62.03)	120 (64.5)	107 (59.1)
Right	211 (38.1)	71 (37.97)	66 (35.5)	74 (40.9)
Missing, No.	19	5	8	6
Differentiation				
Poor	67 (12.2)	22 (12.1)	22 (11.7)	23 (12.8)
Moderate	374 (68.0)	121 (66.5)	132 (70.2)	121 (67.2)
Good	109 (19.8)	39 (21.4)	34 (18.1)	36 (20.0)
Missing, No.	23	10	6	7
ECOG performance status				
0	464 (82.1)	161 (84.7)	160 (82.9)	143 (78.6)
1	101 (17.9)	29 (15.3)	33 (17.1)	39 (21.4)
Missing, No.	8	2	1	5
T stage^a^				
3	362 (63.2)	124 (64.6)	119 (61.3)	119 (63.6)
4	211 (36.8)	68 (35.4)	75 (38.7)	68 (36.4)
Perforation or obstruction^a^				
No	428 (74.7)	145 (75.5)	139 (71.6)	144 (77.0)
Yes	145 (25.3)	47 (24.5)	55 (28.3)	43 (23.0)
Vascular or perineural invasion^a^				
No	456 (79.6)	152 (79.2)	157 (80.9)	147 (78.6)
Yes	117 (20.4)	40 (20.9)	37 (19.1)	40 (21.4)
Examined nodes				
Median (IQR)	14 (8-21)	13 (7-20)	13 (9-21)	14 (7-21)
<12	243 (42.6)	82 (42.7)	83 (43.0)	78 (41.9)
≥12	328 (57.4)	110 (57.3)	110 (57.0)	108 (58.1)
Missing, No.	2	0	1	1

Abbreviations: ECOG, Eastern Cooperative Oncology Group; IQR, interquartile range.

^a^ No data were missing.

### Survival Results

**Figure 2.**
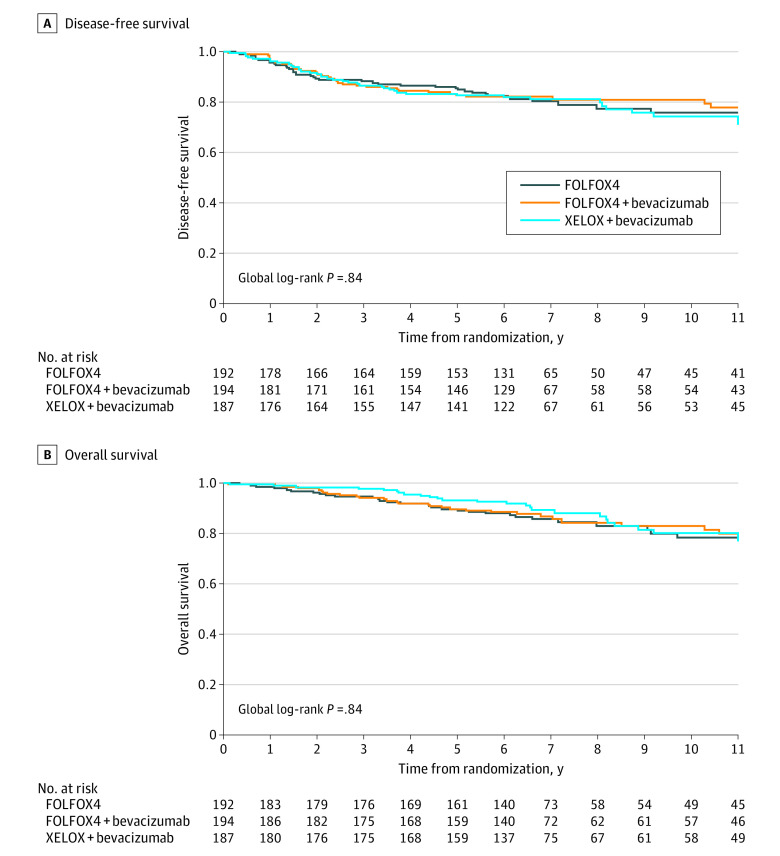
Disease-Free Survival and Overall Survival, According to Treatment Group, in 318 Patients With High-Risk Stage II Colon Cancer FOLFOX4 indicates 5-fluorouracil, leucovorin, and oxaliplatin; XELOX, capecitabine and oxaliplatin.

DFS was observed in 38 patients (19.8%) in the FOLFOX4 group, 36 (18.6%) in the FOLFOX4 with bevacizumab group, and 40 (21.4%) in the XELOX with bevacizumab group. The 3-year DFS rate was 88.2% (95% CI, 83.7%-93.0%) in the FOLFOX4 group, 86.6% (95% CI, 81.8%-91.6%) in the FOLFOX4 with bevacizumab group, and 86.7% (95% CI, 81.8%-91.8%) in the XELOX with bevacizumab group (Figure 2A). The DFS HR was 0.94 (95% CI, 0.59-1.48; *P* = .78) for FOLFOX4 with bevacizumab vs FOLFOX4 alone and 1.07 (95% CI, 0.69-1.67; *P* = .76) for XELOX with bevacizumab vs FOLFOX4 alone.

The 5-year OS rate was 89.7% (95% CI, 85.4%-94.2%) in the FOLFOX4 group, 89.7% (95% CI, 85.4%-94.2%) in the FOLFOX4 with bevacizumab group, and 93.2% (95% CI, 89.6%-97.0%) in the XELOX with bevacizumab group (Figure 2B). The OS HR was 0.92 (95% CI, 0.55-1.55; *P* = .76) for FOLFOX4 with bevacizumab vs FOLFOX4 alone and 0.85 (95% CI, 0.50-1.44; *P* = .55) for XELOX with bevacizumab vs FOLFOX4 alone.

Forest plots for main prognostic factors of DFS and OS for the FOLFOX4 with bevacizumab group and the XELOX with bevacizumab group vs the FOLFOX4 group are presented in eFigure 2 in Supplement 2. There was no benefit of bevacizumab when added to FOLFOX4 in patients with right CC in terms of DFS (HR = 0.50; 95% CI, 0.22-1.18) and OS (HR = 0.37; 95% CI, 0.13-1.02).

### High-Risk Stage II Classification

**Figure 3.**
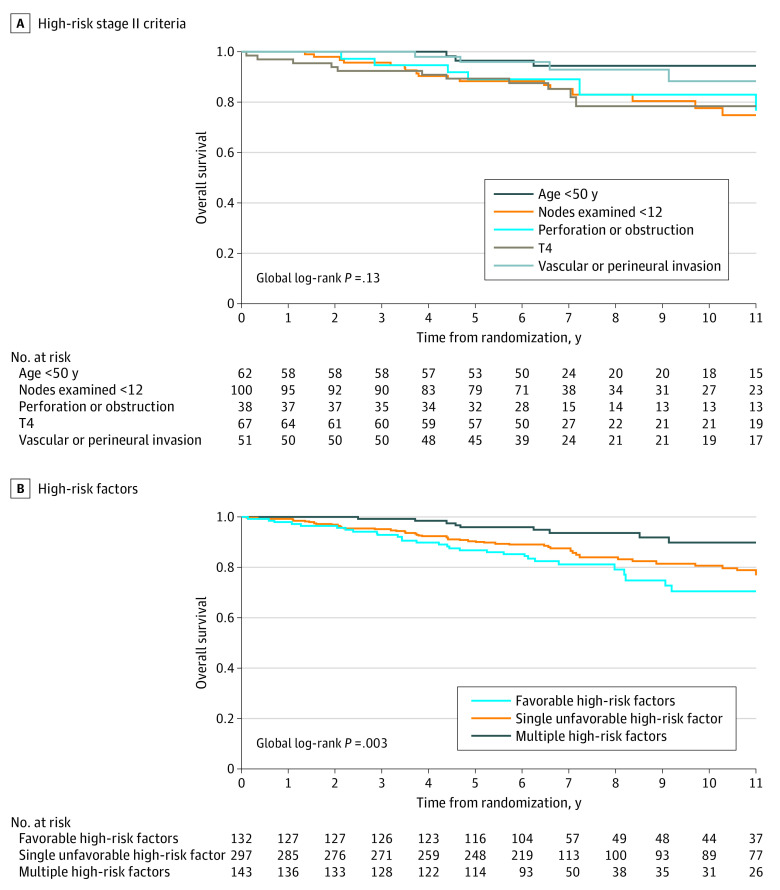
Overall Survival According to High-Risk Stage II Criteria and to Favorable, Single Unfavorable, or Multiple Unfavorable Criteria in Patients With High-Risk Stage II Colon Cancer Favorable high-risk factors included being younger than 50 years and/or having vascular or perineural invasion. Single unfavorable high-risk factors were having fewer than 12 examined nodes, having stage T4 disease, or having perforation or obstruction. Multiple high-risk factors included all other high-risk factor combinations.

Among the 318 patients (55.5%) classified as having high-risk stage II CC based on a single factor, being younger than 50 years was observed in 62 (10.8%); vascular or perineural invasion, 51 (8.9%); stage T4, 67 (11.7%); perforation or obstruction, 38 (6.6%); and fewer than 12 examined lymph nodes in 100 (17.5%). These patients’ OS curves showed that those who were younger than 50 years or who presented with a vascular or perineural invasion had better survival than those with other high-risk factors (Figure 3A). However, the difference was significant only for having fewer than 12 lymph nodes examined (Table 2). Consequently, we defined age and vascular or perineural invasion as favorable high-risk factors and the other high-risk factors (ie, <12 examined lymph nodes, stage T4, and perforation or bowel obstruction) as unfavorable high-risk factors. Comparison of OS for favorable high-risk factors vs a single unfavorable factor and 2 or more high-risk factors was also significant (Figure 3B and Table 2).

**Table 2.** Overall Survival and High-Risk Factors for Patients with Stage II Colon Cancer

Factor	Patients, No. (events)	HR (95% CI)	*P* value
Patients with single high-risk factor (n = 318)			
Age <50 y only	62 (3)	1 [Reference]	NA
<12 Nodes examined	100 (17)	3.64 (1.07-12.43)	.04
Perforation or obstruction	38 (6)	3.15 (0.79-12.60)	.10
Stage T4 disease	67 (11)	3.39 (0.95-12.16)	.06
Vascular or perineural invasion only	51 (4)	1.49 (0.33-6.66)	.60
All patients (N = 573)			
Favorable high-risk factors^a^	132 (9)	1 [Reference]	NA
Single unfavorable high-risk factors^b^	297 (46)	2.40 (1.18-4.91)	.02
Multiple high-risk factors^c^	143 (29)	3.43 (1.62-7.25)	.001

Abbreviations: HR, hazard ratio; NA, not applicable.

^a^ Favorable high-risk factors were being younger than 50 years and/or having vascular or perineural invasion.

^b^ Single unfavorable high-risk factors were having fewer than 12 nodes examined, having perforation or obstruction, or having tumor stage 4.

^c^ This category included all combinations of high-risk factors combination.

### Prognostic Factors

For DFS in univariate analysis, stage T4 vs T3, ECOG performance score 1 vs 0, fewer than 12 vs at least 12 examined nodes, and age 50 years or older vs younger than 50 years were prognostic factors (eTable 1 in Supplement 2). In multivariate analysis, stage T4 vs T3 and fewer than 12 vs at least 12 examined nodes remained prognostic (eTable 2 in Supplement 2).

For OS in univariate analysis, stage T4 vs T3, fewer than 12 vs at least 12 examined nodes, and age 50 years or older vs younger than 50 were prognostic factors (eTable 1 in Supplement 2). In multivariate analysis, stage T4 vs T3, fewer than 12 vs at least 12 examined nodes, and age 50 years or older vs younger than 50 years remained prognostic (eTable 2 in Supplement 2).

### Late Toxic Effects and Cause of Death

Early safety data of high-risk stage II and stage III CC were previously reported.^15^ With a total of 84 deaths, CC-related deaths occurred in 55 patients (65.5%) (18 of 30 [60.0%] in FOLFOX4 group; 19 of 28 [67.9%] in FOLFOX4 with bevacizumab group; 18 of 26 [69.2%] in XELOX with bevacizumab group). Deaths related to cardiomyopathy, congestive heart failure, cardiac ischemia, pulmonary embolism, stroke, intracranial hemorrhage, and sudden deaths were reported in 5 patients (16.7%) in the FOLFOX4 group, 6 patients (21.4%) in the FOLFOX4 with bevacizumab group, and in 1 patient (3.8%) in the XELOX with bevacizumab group. Other noncancer deaths or unknown causes of death occurred in 7 patients (23.3%) in the FOLFOX4 group, 3 patients (10.7%) in the FOLFOX4 with bevacizumab group, and 7 patients (26.9%) in the XELOX with bevacizumab group.

## Discussion

In this study, the 3-year DFS rates in patients with high-risk stage II CC were 88.2% in the FOLFOX4 group, 86.6% in the FOLFOX4 with bevacizumab group, and 86.7% in the XELOX with bevacizumab group. A Will Rogers^20^ stage migration phenomenon is likely, given that these results contrast with the 3-year DFS rates of 81.3% achieved in the fluoropyrimidine-alone group and 86.3% in the same FOLFOX4 regimen observed in patients with high-risk stage II CC in the MOSAIC trial.^4^ In the complete stage II CC population, including high-risk and low-risk patients, the ACCENT analysis of 21 clinical trials with identical treatment (5-FU/LV–based therapy without oxaliplatin), survival rate was better among patients treated from 1996 to 2007 compared with patients treated from 1978 to 1995 (89.0% vs 82.5%; HR, 0.67; 95% CI, 0.58-0.76). This reflects the stage migration over time, challenging historical data related to the benefit of 5-FU/LV–based adjuvant therapy in such patients.^21^ The 2.9% absolute improvement in OS demonstrated for stage II patients with adjuvant chemotherapy in the QUASAR study might be substantially smaller if repeated in the current clinical setting.

Our study does not suggest that bevacizumab may prolong DFS or OS of patients with high-risk stage II CC even if the small number of patients and the lack of formal hypothesis in this exploratory study must make us cautious. However, the effect of bevacizumab is not deleterious, as shown in patients with stage III CC in the recent AVANT study update.^19^ The differential effect of bevacizumab on survival between stage II and III could be partly explained by bevacizumab-induced tumor cell dormancy phenomenon, which makes these cells inaccessible to chemotherapy.^22,23^ The lower risk of having residual cells after the intervention in stage II CC than in stage III CC reduces the potential of this negative effect of tumor dormancy. Besides, we cannot exclude a bevacizumab differential effect according to primary tumor sidedness with a potential benefit in high-risk stage II right CC. This is in accordance with the different prognosis between right and left CC in all settings, which is driven by tumor biology.

The adverse effects remained moderate. The most serious adverse effects associated with bevacizumab (ie, intestinal perforation and arterial thromboses) were not increased compared with the control group.^15^ The younger age of the study population as well as their generally good health could explain this observation.

Of note, statistically nonsignificant but numerically higher OS rates were observed in the XELOX with bevacizumab group than in either FOLFOX4 group. A potential superiority of XELOX over FOLFOX has been suggested among patients with low-risk stage III CC from the IDEA meta-analysis.^24^ However, in the IDEA study, patients were not randomized between XELOX and FOLFOX but between 3-month and 6-month durations of chemotherapy.

### Limitations

This study has limitations, particularly the debatable definition of high-risk stage II CC. In this study, the high-risk criteria were being younger than 50 years; having a local, stage T4 tumor; having vascular or perineural invasion; having perforation or bowel obstruction; and having fewer than 12 lymph nodes examined. However, among our patients, being younger than 50 years had a good prognosis, and neither vascular nor perineural tumoral invasion conferred negative outcomes. To be more consistent with recent staging, it would have been also judicious to distinguish between tumors with grade T4a and T4b and to separate obstruction from perforation.^25^

Furthermore, the very high prognostic value of circulating tumor DNA (ctDNA) for OS and recurrence-free survival shown in small cohorts led to the development of larger studies to confirm this prognostic value in stage II and III CC.^26,27,28^ The ultimate goal of these studies is to explore the interest of ctDNA-driven adjuvant treatment.^29^

## Conclusions

In this secondary analysis of data from the AVANT trial, bevacizumab did not improve DFS and OS in the adjuvant setting among patients with high-risk stage II CC, but a potential benefit cannot be excluded in patients with right-sided tumors. A better classification of stage II risk factors, including ctDNA, will be useful for future studies, even if it is worth validating in ongoing studies.

## Notes

Supplement 1. **Trial Protocol**

Supplement 2. **eTable 1. **Univariate Analysis of Prognostic Factors for Disease-Free Survival and Overall Survival **eTable 2. **Multivariate Analysis of Prognostic Factors for Disease-Free Survival and Overall Survival **eFigure 1. **Distribution of Patients According to the Criteria Defining High-Risk Stage II Colon Cancer **eFigure 2. **Forest Plots for Disease-Free Survival and Overall Survival for FOLFOX4 vs FOLFOX4 with Bevacizumab and Disease-Free Survival and Overall Survival for FOLFOX4 vs XELOX with Bevacizumab

Supplement 3. **Data Sharing Statement**
