# Errors in Abstract and Figure

**DOI:** 10.1001/jamanetworkopen.2021.0700

**Published:** 2021-02-09

In the Original Investigation titled “Association of Bevacizumab Plus Oxaliplatin-Based Chemotherapy With Disease-Free Survival and Overall Survival in Patients With Stage II Colon Cancer: A Secondary Analysis of the AVANT Trial,”^1^ published October 19, 2020, there were errors in the Abstract and Figure 3B. The Abstract incorrectly described the results of the Multicenter Study of Oxaliplatin/5FU-LV in the Adjuvant Treatment of Colon Cancer trial as the results of the Bevacizumab-Avastin Adjuvant trial. In Figure 3B, the key reversed the colors for patients with favorable high-risk factors and those with multiple high-risk factors. This article has been corrected.^1^
