# Supplementary material for: Association of Bevacizumab Plus Oxaliplatin-Based Chemotherapy With Disease-Free Survival and Overall Survival in Patients With Stage II Colon Cancer: A Secondary Analysis of the AVANT Trial
Source: JAMA Netw Open. 2020 Oct 19;3(10):e2020425. doi: 10.1001/jamanetworkopen.2020.20425 (PMC7573695; doi:10.1001/jamanetworkopen.2020.20425)
Supplement: Supplement 1. — Trial Protocol [file jamanetwopen-e2020425-s001.pdf]

F HOFFMANN - LA ROCHE AG

CLINICAL STUDY PROTOCOL

A Randomized, Three Arm Multinational Phase III Study to Investigate Bevacizumab (q3w or q2w) in Combination With Either Intermittent Capecitabine Plus Oxaliplatin (XELOX) (q3w) or Fluorouracil/ Leucovorin With Oxaliplatin (FOLFOX-4) Versus FOLFOX-4 Regimen Alone as Adjuvant Chemotherapy in Colon Carcinoma.

The AVANT Study

PROTOCOL NUMBER BO 17920

RO 4876646

BEVACIZUMAB

PROTOCOL APPROVAL

Protocol Number/ Version:	BO 17920 / Version A
Date:	30 September 2004
Clinical Science Leader :	Harald Weber, MD
Project Statistician:	Cornelia Irl, PhD

Confidentiality Statement

The information contained in this document, especially unpublished data, is the property of F. Hoffmann-La Roche AG (or under its control), and therefore provided to you in confidence as an investigator, potential investigator or consultant, for review by you, your staff and an applicable Independent Ethics Committee/Institutional Review Board. It is understood that this information will not be disclosed to others without written authorization from Roche except to the extent necessary to obtain informed consent from those persons to whom the drug may be administered.

SYNOPSIS OF PROTOCOL BO 17920

TITLE	A Randomized, Three Arm Multinational Phase III Study to Investigate Bevacizumab (q3w or q2w) in Combination With Either Intermittent Capecitabine Plus Oxaliplatin (XELOX) (q3w) or Fluorouracil/ Leucovorin With Oxaliplatin (FOLFOX-4) Versus FOLFOX-4 Regimen Alone as Adjuvant Chemotherapy in Colon Carcinoma.
SPONSOR	F. Hoffmann-La Roche AG CLINICAL PHASE III<br>Roche Global Development
INDICATION	Adjuvant treatment for patients who have undergone surgery for Colon cancer, AJCC/UICC high-risk Stage II & stage III
OBJECTIVES	Primary:<br>1. To demonstrate that the combination of bevacizumab and FOLFOX-4 is superior to FOLFOX-4 alone in terms of disease-free survival in chemotherapy-naïve patients who underwent surgery with curative intent for colon carcinoma.<br>2. To demonstrate that the combination of bevacizumab and XELOX is superior to FOLFOX-4 alone in terms of disease-free survival in chemotherapy-naïve patients who underwent surgery with curative intent for colon carcinoma.<br>Secondary:<br>• To demonstrate that the combination of bevacizumab and FOLFOX-4 is superior to FOLFOX-4 alone in terms of overall survival in chemotherapy-naïve patients who underwent surgery with curative intent for colon carcinoma.<br>• To demonstrate that the combination of bevacizumab and XELOX is superior to FOLFOX-4 alone in terms of overall survival in chemotherapy-naïve patients who underwent surgery with curative intent for colon carcinoma.<br>• In case both primary objectives are achieved, to further investigate if the combination of bevacizumab and XELOX is at least as efficacious as the combination of bevacizumab and FOLFOX-4 in terms of disease-free survival and overall survival<br>• To evaluate and compare the safety profiles of the treatment groups.<br>• To evaluate the immunogenicity of bevacizumab measured as induction of HAHA.<br>Tertiary objectives:<br>• To evaluate and compare the perceived convenience and satisfaction with chemotherapy for patients in the three treatment groups.<br>• To evaluate and compare medical care utilization in the three treatment groups.

TRIAL DESIGN	Multicenter, multinational, randomized, comparative efficacy and safety 3-arm study. The treatment phase consists of two parts of 24 weeks for a total of 48 weeks:<br>• Treatment with either FOLFOX-4, FOLFOX-4 in combination with bevacizumab, or XELOX in combination with bevacizumab<br>• Observation period for patients assigned to arm A or a treatment period with bevacizumab as a single agent in arms B and C.<br>The study regimens include:<br>• Arm A (FOLFOX-4): Oxaliplatin, leucovorin (LV) and 5-fluorouracil (5-FU).<br>• Arm B (FOLFOX-4+bev): Bevacizumab, oxaliplatin, leucovorin (LV) and 5-fluorouracil (5-FU).<br>• Arm C (XELOX+bev): Bevacizumab, oxaliplatin in combination with capecitabine.<br>The cycle duration during week 1-24 in arms A and B is 2 weeks with a total of 12 planned cycles, in arm C it is 3 weeks and a total of 8 cycles. The cycle duration during week 25 to 48 of bevacizumab as a single agent will be 3 weeks for a total of 8 cycles for patients on arms B and C. In arm A the visit schedule for patients on observation only will be 3 weeks as well.<br>Those patients who experience a confirmed recurrence, occurrence of a new colorectal cancer during therapy, or experience unacceptable toxicity will be taken off study treatment. Patients will be assessed for recurrence/new occurrence of colorectal cancer before and at 6 months after randomization. Further assessment time points for recurrence/new occurrence of colorectal cancer and survival are 1 year, 1.5, 2, 2.5, 3, 3.5 and 4 years after randomization and yearly afterwards. CEA will be analyzed every 6 months. The primary analysis will be performed after approximately 836 events have occurred in patients with stage III disease. Then patients will be followed for recurrence/appearance of new colorectal cancer and survival for additional 2 years after the primary analysis. In case of a confirmed recurrence/appearance of new colorectal cancer patients will be followed for survival until the end of study follow up period as well.
NUMBER OF SUBJECTS	3450 patients (1150 per treatment arm)
TARGET POPULATION	Male and female outpatients ≥ 18 years of age with histological confirmed colon carcinoma who have had potentially curative surgery not less than 4 and not more than 8 weeks prior to randomization.<br>Patients must not have previous anti-angiogenic treatment for any malignancy; cytotoxic chemotherapy, radiotherapy or immunotherapy for colon cancer. ECOG performance status ≤ 1 with no evidence of remaining tumour.
LENGTH OF STUDY	The total recruitment period is expected to be 23 months. Recruitment will stop when at least 3450 patients have been randomized. The primary efficacy analysis will take place when approximately 836 events have occurred in patients with stage III disease. This is expected to take place after approximately 36 months after the last patient has been randomized. Thereafter, patients will be followed for recurrence/appearance of new colorectal cancer and survival for further 2 years (end of the study follow up).

INVESTIGATIONAL PRODUCT(S) DOSE/ ROUTE/ REGIMEN	*Arm A (FOLFOX-4):*<br>*Week 1 – 24 (FOLFOX-4):* Oxaliplatin will be administered as an 85 mg/m2 intravenous infusion over 2 hours concomitantly with leucovorin (LV), as a 200 mg/m2 infusion over 2 hours, followed by 5-fluorouracil (5-FU), given as a 400 mg/m2 bolus injection, and then as a 600 mg/m2 continuous infusion over 22 hours. LV 200 mg/m2 (alone), followed by 5-FU 400 mg/m2 bolus injection, and 5-FU 600 mg/m2 continuous infusion are repeated on day 2. Cycle length is 2 weeks comprising approximately 48 hours of infusion and 12 days of rest. Cycles to be repeated every second week for a total of 12 cycles (24 weeks).<br>*Week 25 – 48 :* Observation only<br><br>*Arm B (FOLFOX-4+Bev):*<br>*Week 1 – 24* (*FOLFOX*-4+Bev)*:*<br>Bevacizumab at 5 mg/kg will be administered as an intravenous infusion over 30 – 90 minutes followed by oxaliplatin, administered as an 85 mg/m2 intravenous infusion over 2 hours (on day 1 only) concomitantly with leucovorin (LV), as a 200 mg/m2 infusion over 2 hours, followed by 5-fluorouracil (5-FU), given as a 400 mg/m2 bolus injection, and then as a 600 mg/m2 continuous infusion over 22 hours. LV 200 mg/m2 (alone), followed by 5-FU 400 mg/m2 bolus injection, and 5-FU 600 mg/m2 continuous infusion are repeated on day 2. Cycle length is 2 weeks comprising approximately 49 hours of infusion and 12 days of rest. Cycles to be repeated every second week for a total of 12 cycles (24 weeks).<br>*Week 25 – 48 (Bev Monotherapy):*<br>Bevacizumab at 7.5 mg/kg will be *administered* as an intravenous infusion over 30 minutes. Cycle length is 3 weeks. Cycles to be repeated every 3 week for a total of 8 cycles (24 weeks).<br><br>*Arm C (XELOX+Bev):*<br>*Week 1 – 24* (XELOX+Bev):<br>Bevacizumab at 7.5 mg/kg will be administered as an intravenous infusion over 30 – 90 minutes followed by oxaliplatin administered as a 130 mg/m2 intravenous infusion over 2 hours (day 1 every 3 weeks) in combination with capecitabine, which will be administered orally at a dose of 1000 mg/m2 twice-daily (equivalent to a total daily dose of 2000 mg/m2), with first dose the evening of day 1 and last dose the morning of day 15, given as intermittent treatment (3-week cycles consisting of 2 weeks of treatment followed by 1 week without treatment), for a total of 8 cycles (24 weeks).<br>*Week 25 –* 48 *(Bev Monotherapy):*<br>Bevacizumab at 7.5 mg/kg will be administered as an intravenous infusion over 30 minutes. Cycle length is 3 weeks. Cycles to be repeated every 3 week for a total of 8 cycles (24 weeks).

ASSESSMENTS OF:	
- EFFICACY	Primary: Disease-free survival<br>Secondary: Overall survival
- SAFETY	Adverse events and laboratory abnormalities, graded according to the CTCAE v 3.0.
- PHARMACOKINETICS/ PHARMACODYNAMICS	Induction of HAHA by bevacizumab
- QUALITY OF LIFE/	An assessment of the Chemotherapy Convenience and Satisfaction by questionnaire (CCSQ) will be performed. The objective will be to summarize and evaluate treatment group differences in chemotherapy convenience and satisfaction.<br>.
PHARMACOECONOMICS	Pharmacoeconomic analyses will be performed on medical care utilization (MCU). The objective will be to summarize and evaluate treatment group differences in total resource use, and more specifically, in resource use associated with (1) treatment of drug-related adverse events and in (2) resource use for the routine drug administration and monitoring associated with study drugs
PATIENT ASSIGNMENT	Patients will be randomly assigned to one of the three treatment arms (A, B or C).<br>Stratification will done by:<br>• geographic region,<br>• AJCC/UICC stage (high-risk stage II vs. stage III N1 vs. stage III N2),<br>Patient numbers will be chronologically assigned within each center as patients are enrolled in the study. All patient numbers across the study will be unique.

PROCEDURES (summary):

Written informed consent will be obtained before any study specific procedures are undertaken. Written informed consent must be obtained prior to the patient undergoing any study-specific procedures.

Screening/Baseline tests: The screening procedure may be done in different stages.

– Tumour assessments: abdominal & pelvic CT/MRI AND chest CT/MRI or X-ray may be obtained up to 3 weeks before surgery (Note: surgery should occur within 8 weeks before randomization, allowing a maximum of 11 weeks between scans and randomization), but may be done after surgery (up to randomization) as well.
– Chest X-ray safety assessment must be done within 3 weeks before randomization. If chest CT/MRI or X-ray are performed within 3 weeks before randomization (e.g. as tumour assessment), no additional chest X-ray is necessary.
– Assessments to be made up to 14 days before randomization include: demographic data, medical history, cancer/treatment history, concomitant disease/treatment, physical examination (including basic neurological exam by investigator), electrocardiogram (ECG) and CEA determination.
– Assessments to be made within 7 days before randomization: height, weight, vital signs (body temperature, blood pressure, and pulse/heart rate), ECOG Performance Status, hematology, blood chemistry (including creatinine clearance calculation), INR, APTT and plasma D-dimer, serum pregnancy test (for all women less than 2

years amenorrheic), dipstick urinalysis for proteinuria, 24-hour urine collection for determination of total protein and CCSQ baseline question.

– Urine pregnancy test is required prior to first administration of study treatment if more than 7 days have elapsed from baseline serum pregnancy test.
– Arms B and C: Serum will be collected prior to first administration of bevacizumab for central analysis of antibodies to rHuMAb VEGF.

Inclusion Criteria: In order to be eligible for the trial patients have to fulfill the following criteria:

1. Signed written informed consent obtained prior to any study specific screening procedures.
2. Patient must be willing and able to comply with the protocol.
3. Age ≥ 18.
4. Histologically confirmed colon carcinoma, AJCC/UICC Stage II or Stage III defined as a tumour location ≥15 cm from the anal verge by endoscopy or above the peritoneal reflection at surgery. The patient must not be a candidate for (neo) adjuvant radiotherapy.

Note! Stage II patients have to be considered as high-risk patients fulfilling one of the following criteria:

– T4 tumours,
– Patients presenting with bowel obstruction or perforation,
– Histological signs of vascular invasion (i.e. blood and lymphatic vessels) or perineural invasion,
– Patients aged less than 50 years,
– Patients with sub-optimal surgery (less than 12 nodes analyzed).

5. Curative surgery not less than 4 and not more than 8 weeks prior to randomization.
6. ECOG performance status 0 or 1.
7. Life expectancy of ≥ 5 years.

Exclusion Criteria: Patients presenting with any of the following criteria are not eligible for the study:

1. Macroscopic or microscopic evidence of remaining tumour. Patients should never have had any evidence of metastatic disease (including presence of tumour cells in the ascites). The isolated finding of cytokeratin positive cells in bone marrow is not considered evidence of metastatic disease for purposes of this study.
2. Carcinoembryonic antigen > 1.5 x ULN after surgery (during screening period).
3. For patients with colostomy, unwilling to delay revision until at least 28 days after treatment completion.
4. Major surgical procedure, open biopsy, or significant traumatic injury within 28 days prior to study treatment start, not fully healed wounds, or anticipation of the need for major surgical procedure during the course of the study. CVAD for chemotherapy administration must be inserted at least 2 days prior to treatment start. For details refer to Section 5.1.2.
5. Previous anti-angiogenic treatment for any malignancy; cytotoxic chemotherapy, radiotherapy or immunotherapy for colon cancer.
6. Other malignancies within the last 5 years (other than curatively treated basal cell carcinoma of the skin and/or in situ carcinoma of the cervix).
7. Females with a positive or no pregnancy test (within 7 days before treatment start) unless childbearing potential can be otherwise excluded (postmenopausal i.e. amenorrheic for at least 2 years, hysterectomy or oophorectomy).
8. Lactating women.
9. Fertile women (< 2 years after last menstruation) and men of childbearing potential not willing to use effective means of contraception.
10. History or evidence upon physical examination of CNS disease (e.g., primary brain tumour, seizure not controlled with standard medical therapy, any brain metastases).
11. History of psychiatric disability judged by the investigator to be clinically significant, precluding informed consent or interfering with compliance for oral drug intake.
12. Clinically significant (i.e. active) cardiovascular disease e.g. cerebrovascular accidents (≤ 6 months prior to randomisation), myocardial infarction (≤ 1 year prior to randomisation), uncontrolled hypertension while receiving chronic medication, unstable angina, New York Heart Association (NYHA) Grade II or greater congestive heart failure, or serious cardiac arrhythmia requiring medication.
13. Lack of physical integrity of the upper gastro-intestinal tract, malabsorption syndrome, or inability to take oral medication.
14. Interstitial pneumonia or extensive symptomatic fibrosis of the lungs.
15. Known peripheral neuropathy ≥ CTCAE v 3.0 Grade 1. Absence of deep tendon reflexes (DTRs) as the sole neurological abnormality does not render the patient ineligible.

16. Organ allografts requiring immunosuppressive therapy.
17. Serious, non-healing wound, ulcer, or bone fracture.
18. Evidence of bleeding diathesis or coagulopathy.
19. Current or recent (within 10 days prior to study treatment start) use of full-dose oral or parenteral anticoagulants or thrombolytic agent for therapeutic purposes.
20. Chronic, daily treatment with high-dose aspirin (> 325mg/day) or nonsteroidal anti-inflammatory medications (those known to inhibit platelet function at doses used to treat chronic inflammatory diseases). Patients can be rendered eligible by changing the treatment to COX II inhibitors.
21. Chronic treatment with corticosteroids (dose of ≥ 10 mg/day methylprednisolone equivalent) (excluding inhaled steroids).
22. Serious intercurrent infections (uncontrolled or requiring treatment).
23. Known dihydropyrimidine dehydrogenase (DPD) deficiency.
24. Current or recent (within the 28 days prior to randomization) treatment with another investigational drug or participation in another investigational study.
25. Patients with known allergy to Chinese hamster ovary cell proteins or other recombinant human or humanized antibodies or to any excipients of bevacizumab formulation, platinum compounds or to any other components of the study drugs.
26. History or presence of other disease, metabolic dysfunction, physical examination finding, or clinical laboratory finding giving reasonable suspicion of a disease or condition that contraindicates use of an investigational drug or patient at high risk from treatment complications.
27. Presence of proteinuria at baseline as defined by:
    - Patients with > 1g of protein/24 hr by a 24-hour urine collection.
28. Any laboratory values at baseline are as follows:

    Haematology:
    – Absolute neutrophil count (ANC) < 1.5 x 109/L
    – Platelet count < 100 x 10^9/L
    – Haemoglobin < 9 g/dL (may be transfused to maintain or exceed this level)
    – International Normalized Ratio (INR) > 1.5
    – APTT ≥ 1.5 x Upper Limit of Normal (ULN)

    Biochemistry:
    - Total bilirubin > 1.5 x ULN
    – AST, ALT > 2.5 x ULN
    – Alkaline phosphatase > 2.5 x ULN
    – Serum Creatinine > 1.5 x ULN or creatinine clearance ≤ 50 mL/min (calculated according to Cockroft and Gault, Appendix 6).

Assessments during Study Treatment Phase:

Note: the observation period in Arm A is part of study treatment phase:

- Adverse events will be collected continuously during the Study Treatment Phase and up to 28 days after the end of Study Treatment Phase, and followed up until the event is either resolved or adequately explained, even after the patient has completed his/her study treatment.
- Concomitant diseases / treatment and compliance to study drugs will be monitored continuously during the Study Treatment Phase.
- Vital signs, weight, ECOG Performance Status, hematology, INR (only for patients receiving oral coumarin-derivative anticoagulant therapy), serum chemistries, and urinalysis will be performed for each patient according to the assigned schedule.
- ECG (obligatory at week 28 in arm A) and chest X-ray will be performed as clinically indicated.
- Nature and duration of any hospitalization, treatment of any adverse event and nature and duration of any outpatient care will be recorded during the Study Treatment Phase.
- An assessment of the chemotherapy convenience and satisfaction via questionnaire (CCSQ) will be performed.

Tumour assessments (*abdominal & pelvic* CT/MRI or US AND *chest* CT/MRI or X-ray) and a CEA determination should be performed 6 and 12 months after randomization or if the patient shows signs of a recurrence /new colorectal cancer (e.g. clinical status). Possible re-operation or/and further cancer therapy will be recorded.

Assessments during Follow up Phase: During the Follow-Up Phase the following will apply:

- CEA determinations will be done at 6 and 12 months after randomization, subsequently every 6 months (until a confirmed recurrence/new CRC).
- Tumour assessments will be done 6 months after randomization, then every 6 months for the first 4 years after randomization, and then on a yearly basis (until a confirmed recurrence/new CRC).
- Additional tumour assessments including endoscopy may be required if the patient shows signs of a recurrence (e.g. clinical status or increase of CEA levels) or a new colorectal cancer. All assessments, i.e. also non-protocol specified, used to confirm a recurrence/new tumour should be recorded in the Case Report Form.
- HAHA determination will be done in arms B and C, at 1 and 1.5 years after randomization.
- Extended follow-up of hypertension, proteinuria & wound healing complication until resolution.
- Additional cancer therapy to be recorded as it occurs.
- Survival status should be assessed every 6 months in the first 4 years after randomization and then annually thereafter (may be by telephone contact rather than visit).

Thereafter, patients will be followed for recurrence/appearance of new colorectal cancer and survival for further 2 years (end of the study follow up).

STATISTICAL ANALYSES:

Primary analysis:

Using the closed test procedure, superiority of bevacizumab plus FOLFOX-4 to FOLFOX-4 alone and superiority of bevacizumab plus XELOX to FOLFOX-4 alone in terms of disease-free survival will be tested based on the log-rank test. This analysis will be based on patients with stage III disease only.

Secondary analyses:

Using the closed test procedure, superiority of bevacizumab plus FOLFOX-4 to FOLFOX-4 alone and superiority of bevacizumab plus XELOX to FOLFOX-4 alone in terms of overall survival will be tested based on the log-rank test. Hazard ratio and 95% confidence intervals will be calculated for the comparison of bevacizumab plus FOLFOX-4 versus bevacizumab plus XELOX for disease free survival and overall survival. All these analyses will be based on patients with stage III disease only.

Safety parameters:

- Adverse events,
- Laboratory abnormalities based on CTCAE v 3.0 grading system,
- Vital signs.

Tertiary Variables:

- Medical care utilization,
- Chemotherapy Convenience and Satisfaction Assessment.

Table of Contents

1. BACKGROUND AND RATIONALE 25
1.1 Colon Cancer 25
1.2 Adjuvant Colon Cancer Therapy 25
1.3 Bevacizumab (rHuMAb VEGF) 27
1.3.1 Vascular Endothelial Growth Factor (VEGF) 27
1.3.2 Anti-VEGF Monoclonal Antibody - rHuMAb VEGF 28
1.3.3 Preclinical Safety Studies - Bevacizumab 29
1.3.4 Clinical Studies - Bevacizumab 30
1.3.4.1 Clinical Pharmacology - Bevacizumab 30
1.3.4.2 Clinical Studies in Colorectal Cancer - Bevacizumab 32
1.4 Capecitabine - Xeloda® 35
1.4.1 Clinical Pharmacokinetics and Metabolism of Capecitabine 35
1.4.2 Clinical Studies of Capecitabine Monotherapy in Metastatic Colorectal Cancer 36
1.4.3 Clinical Studies of Capecitabine Monotherapy in Adjuvant Colon Cancer 37
1.5 Oxaliplatin - Eloxatin® 37
1.5.1 Clinical Studies of Oxaliplatin Monotherapy in Colorectal Cancer 38
1.5.2 Clinical Studies of First-line Oxaliplatin in Combination with 5 FU/LV vs. 5-FU/LV Monotherapy in Colorectal Cancer 38
1.6 Clinical Studies of Combination Capecitabine and Oxaliplatin (XELOX) in Colorectal Cancer 38
1.7 Rationale 39
1.7.1 Rationale for the Study and Study Design 39
1.7.2 Rationale for Dosage Selection 40

2. OBJECTIVES OF THE STUDY 40

3. STUDY DESIGN 41
3.1 Overview of Study Design and Dosing Regimen 41
3.2 Number of Patients/Assignment to Treatment Groups 42

4. STUDY POPULATION 43
4.1 Target Population 43
4.2 Inclusion Criteria 43
4.3 Exclusion Criteria 44
4.4 Concomitant Medication, Intervention and Treatment 46
4.4.1 Supportive Measures 46
4.4.2 Oral Coumarin-Derived Anticoagulants, Heparin, Aspirin and Non-Steroidal Anti-Inflammatory Drugs 47
4.4.3 Phenytoin 47

4.4.4 Antivirals and Antiprotozoals 48
4.4.5 Gastro-intestinal Drugs 48
4.4.6 Other Anticancer Therapies 48

5. SCHEDULE OF ASSESSMENTS AND PROCEDURES 48

5.1 Screening Examination and Eligibility Screening Form 58
5.1.1 Screening Procedures for Study Entry 58
5.1.2 Central Venous Access Device (CVAD) 59
5.2 Study Assessments 59
5.2.1 Clinical Assessments 59
5.2.1.1 Assessments of Efficacy and Recurrence 59
5.2.1.2 Standard Clinical Assessments (Safety Assessments excluding laboratory exams) 60
5.2.2 Laboratory Assessments 61
5.2.3 End of Treatment Safety Assessment 61
5.2.4 Follow-up Phase 62
5.2.4.1 Follow up of Premature Withdrawals (see 7.4) 63
5.2.5 Additional Assessments during Treatment Phase 63
5.2.6 Additional Assessments during Follow-up Phase 63
5.2.7 Anti-bevacizumab Antibodies (HAHA) 64
5.2.8 Pharmacoeconomic Assessments 64
5.2.9 Chemotherapy Convenience and Satisfaction Assessment 64

6. INVESTIGATIONAL REGIMENS 64

6.1 Dose and Schedule of Study Medication 65
6.1.1 Specification of Units 66
6.1.2 Study Treatment Duration 66
6.2 Arm A: FOLFOX-4 66
6.2.1 Week 1 - 24 66
6.2.1.1 Oxaliplatin Dosing Instructions 67
6.2.1.2 Dosing Procedures 67
6.2.2 Week 25 - 48 67
6.3 Arm B: FOLFOX-4+Bev 67
6.3.1 Week 1 - 24 67
6.3.1.1 Dosing instructions for Bevacizumab 68
6.3.1.2 Oxaliplatin Dosing Instructions 68
6.3.1.3 Dosing Procedures 68
6.3.2 Week 25 - 48 69
6.3.2.1 Dosing instructions for Bevacizumab 69
6.3.2.2 Dosing Procedures 69
6.4 Arm C: XELOX+Bev 69

6.4.1 Week 1 - 24 69
6.4.1.1 Dosing instructions for Bevacizumab 70
6.4.1.2 Oxaliplatin Dosing Instructions 70
6.4.1.3 Capecitabine Dosing Instructions 70
6.4.1.4 Route of Administration 70
6.4.2 Week 25 - 48 70
6.4.2.1 Dosing instructions for Bevacizumab 71
6.4.2.2 Dosing Procedures 71
6.5 General Bevacizumab Dosing Instructions 71
6.5.1 Permitted Treatment Interruptions 71
6.5.2 Dosage and Method of Administration 71
6.6 General Oxaliplatin Dosing Instructions 72
6.7 Preparation and Administration of Test Drug and Comparator(s) 73
6.7.1 Preparation of Bevacizumab 73
6.7.2 Preparation of Oxaliplatin 74
6.7.2.1 Reconstitution of Oxaliplatin 74
6.7.2.2 Incompatibilities/Precautions 74
6.7.3 5-FU and Leucovorin 74
6.8 Storage Conditions 74
6.8.1 Capecitabine Storage 74
6.8.2 Bevacizumab Storage 74
6.8.3 Oxaliplatin Storage 75
6.8.4 5-FU and Leucovorin Storage 75
6.9 Blinding and Randomization 75
6.10 Drug Accountability 76
6.10.1 5-FU/LV accountability (arms A and B) 76
6.11 Capecitabine Compliance (arm C) 76
6.11.1 Dispensing of capecitabine 77
6.11.2 Accountability of capecitabine supply 77

7. SAFETY INSTRUCTIONS AND GUIDANCE 77

7.1 Adverse Events and Laboratory Abnormalities 77
7.1.1 Clinical Adverse Events 77
7.1.2 Laboratory Test Abnormalities 78
7.2 Handling of Safety Parameters 79
7.2.1 Serious Adverse Events 79
7.2.1.1 Immediately Reportable to Roche 79
7.2.1.2 Project Specific Adverse Event Definition 79
7.2.2 Targeted Events 79
7.2.3 Reporting and Follow-up of Adverse Events after the End of Study Treatment Phase 80

7.2.3.1 Adverse Events in general 80
7.2.3.2 Targeted Events - Reporting and Follow-up Procedure 80
7.2.3.3 Treatment of Adverse Events 81
7.2.4 Follow-up of Abnormal Laboratory Test Values 81
7.2.5 Pregnancy 81
7.3 Dose Modifications for Toxicity 82
7.3.1 General Notes Regarding Dose Modifications 82
7.3.2 Bevacizumab Toxicities (applicable to arms B and C) 83
7.3.2.1 Treatment modifications in case of events attributable to bevacizumab 83
7.3.2.2 Hemorrhage 84
7.3.2.3 Thrombosis/Embolism 84
7.3.2.4 Hypertension 85
7.3.2.5 Proteinuria 85
7.3.2.6 Gastro-intestinal Perforations 86
7.3.2.7 Wound Healing Complications 86
7.3.2.8 Fistula or intra-abdominal abscess 87
7.3.2.9 Infusion-related or allergic reactions 87
7.3.2.10 Summary of Bevacizumab Schedule and Dosage Modification . . 87
7.3.3 Oxaliplatin Toxicities: General Considerations 90
7.3.3.1 Peripheral neuropathy 90
7.3.3.2 Laryngopharyngeal dysesthesias 90
7.3.3.3 Allergic reactions 90
7.3.3.4 Pulmonary fibrosis 91
7.3.3.5 Nausea/vomiting 91
7.3.4 FOLFOX-4 & FOLFOX-4+Bev (arms A & B): Dose Modifications 91
7.3.4.1 Hematological Toxicity in FOLFOX containing regimen 92
7.3.4.2 Non-Hematological Toxicity in FOLFOX containing regimen . . . 92
7.3.4.3 Neurologic toxicity 94
7.3.4.4 Other Toxicities or Miscellaneous Events 94
7.3.5 XELOX+Bev (arm C): Dose Modifications 95
7.3.5.1 Dose Modifications for Hematologic Toxicity 95
7.3.5.2 Capecitabine: Dose Modifications for Non-hematological Toxicity 97
7.3.5.3 Oxaliplatin: Dose Modifications for Non-hematological Toxicity . 99
7.3.6 Treatment schedule modifications 101
7.3.6.1 Bevacizumab-related specific treatment modifications 102
7.3.6.2 Oxaliplatin-related specific treatment modifications: 102
7.3.7 Dose Escalations for Capecitabine or 5-FU/LV after Discontinuation of Oxaliplatin 102
7.4 Criteria for Premature Withdrawal from Study Treatment 103
7.5 Warnings and Precautions 103

7.5.1 Bevacizumab Precautions 103
7.5.2 Capecitabine Precautions 109
7.5.3 Oxaliplatin Precautions 111
7.5.4 5-FU/LV Precautions 112

8. STATISTICAL CONSIDERATIONS AND ANALYTICAL PLAN 113

8.1 Primary and Secondary Study Variables 113
8.1.1 Primary Variable 113
8.1.2 Secondary Variable 113
8.1.3 Safety Variables 113
8.1.4 Tertiary Variables 113
8.2 Statistical and Analytical Methods 113
8.2.1 Primary Analysis 113
8.2.2 Secondary Analyses 114
8.2.3 Exploratory Analyses 114
8.2.4 Safety Variables 115
8.2.4.1 Adverse Events, Laboratory Data, Premature Withdrawals 115
8.2.4.2 Vital Signs 115
8.2.4.3 Cumulative Dose, Received versus Planned Dose 116
8.2.5 Other Analyses 116
8.2.5.1 Pharmacoeconomic Analysis 116
8.2.5.2 Chemotherapy Convenience and Satisfaction Assessment 116
8.2.6 Hypothesis testing 116
8.2.7 Types of Analyses 117
8.2.7.1 Intent-to-Treat Population 118
8.2.7.2 Per-Protocol Population 118
8.2.7.3 Safety Population 118
8.2.8 Exclusion of Data from Analysis 118
8.2.9 Interim Analysis 118
8.3 Sample Size 118
8.4 Replacement Policy (Ensuring Adequate Numbers of Evaluable Patients) 119
8.4.1 For Patients 119
8.4.2 For Centres 119

9. DATA QUALITY ASSURANCE 119

10. INDEPENDENT DATA AND SAFETY MONITORING BOARD 120

11. REFERENCES 121

12. ETHICAL ASPECTS 129

12.1 Local Regulations/Declaration of Helsinki 129
12.2 Informed Consent 129
12.3 Independent Ethics Committees/Institutional Review Board 129

13. CONDITIONS FOR MODIFYING THE PROTOCOL. 130

14. CONDITIONS FOR TERMINATING THE STUDY 130

15. STUDY DOCUMENTATION, CRFS AND RECORD KEEPING 130
15.1 Investigator's Files / Retention of Documents 130
15.2 Source Documents and Background Data 131
15.3 Audits and Inspections. 131
15.4 Case Report Forms 131

16. MONITORING THE STUDY 132

17. CONFIDENTIALITY OF TRIAL DOCUMENTS AND SUBJECT RECORDS 132

18. PUBLICATION OF DATA AND PROTECTION OF TRADE SECRETS 132

List of Tables

Table 1 Schedules of 5-FU/LV in adjuvant colon cancer regimens 27
Table 2 Predicted Bevacizumab Pharmacokinetic Parameters at Steady-state . 31
Table 3 Bevacizumab Minimum and Maximum Concentrations at Steady-state in Clinical Trials 32
Table 4 Efficacy Results in the Phase II Metastatic CRC Study AVF0780g . . . 33
Table 5 ECOG study E3200 interim safety analysis - Grade 3/4 adverse events 34
Table 6 Schedule of Assessments: Week 1 - 24Arm A: FOLFOX-4 49
Table 7 Schedule of Assessments: Week 1 - 24Arm B: FOLFOX-4 + bevacizumab 51
Table 8 Schedule of Assessments: Week 1 - 24Arm C, XELOX + bevacizumab 53
Table 9 Schedule of Assessments: Week 25 - 48 Arm A: Observation Only . . 55
Table 10 Schedule of Assessments: Week 25 - 48Arm B & Arm C: Bevacizumab Monotherapy 56
Table 11 Schedule of Assessments: Follow - Up Phase All Treatment Arms . . 57
Table 12 Bevacizumab Schedule Modification Due to Adverse Events 88
Table 13 Hematologic Toxicities: Oxaliplatin + 5-FU Dose Reductions for FOLFOX regimen. 92
Table 14 Non-Hematological Toxicities: Oxaliplatin + 5-FU/LV Dose Reductions for FOLFOX regimen 93
Table 15 Neurologic Toxicity Scale: Oxaliplatin Dose Adjustments for FOLFOX regimen. 94
Table 16 Febrile Neutropenia (occurring anytime during treatment): Dose Modifications for XELOX regimen 95
Table 17 Neutropenia: Dose Modifications for XELOX regimen 96
Table 18 Thrombocytopenia and Anemia: Dose Modifications for XELOX regimen 96
Table 19 Non-hematologic AEs: Dose Adjustments for Capecitabine 98
Table 20 Oxaliplatin Dose Adjustments for Non- hematologic AEs (Arm C). . 100
Table 21 Neurologic Toxicity Scale for Oxaliplatin Dose Adjustments (Arm C). 101

List of Figures

Figure 1 Predicted Bevacizumab Concentrations When Given Every 2 or 3 Weeks . . . 31
Figure 2 Overview of Study Schedule and Treatment Order . . . 42
Figure 3 Arm A: Week 1-24 FOLFOX-4 dosing schedule. . . . 66
Figure 4 Arm B Week 1 - 24: FOLFOX-4 + Bev dosing schedule . . . 68
Figure 5 Arm B Week 25- 48: Bev dosing schedule . . . 69
Figure 6 Arm C Week 1 - 24: XELOX + Bev dosing schedule . . . 69
Figure 7 Arm C Week 25- 48: Bev dosing schedule . . . 70
Figure 8 Master Label for the Study Drugs . . . 73

List of Appendices

Appendix 1 Adverse Events Categories for Determining Relationship to Test Drug 133
Appendix 2 Serious Adverse Events (SAEs) Reporting Procedure 135
Appendix 3 ICH Guidelines for Clinical Safety Data Management, Definitions and Standards for Expedited Reporting, Topic E2 137
Appendix 4 EU clinical Directives for SARs (Serious Adverse Reactions) management. Definitions and standard for expedited reporting. 139
Appendix 5 Classification of Colon/Rectal Cancer 141
Appendix 6 Calculation of Creatinine Clearance (Cockroft and Gault) 142
Appendix 7 Nomogram for the Determination of the Body Surface Area 143
Appendix 8 Capecitabine Dose Calculation According to Surface Area 144
Appendix 9 Deviations from treatment schedule 145
Appendix 10 Cardiovascular Disease 147
Appendix 11 Monitoring of Proteinuria 148
Appendix 12 Anaphylaxis Precautions 151
Appendix 13 ECOG Performance Status Scale 152
Appendix 14 Human Anti-Human Antibodies to bevacizumab (HAHA) 153
Appendix 15 Chemotherapy Convenience and Satisfaction Questionnaire (CCSQ) 154

GLOSSARY OF ABBREVIATIONS

5-FU	5-fluorouracil
5-HT3	5 hydroxytryptamine 3 receptor
Aci	As clinically indicated
AE	Adverse event
AJCC/UICC	American Joint Cancer Committee/Union Internationale Contre le Cancer
ALAT (SGPT)	Alanine aminotransferase (serum glutamic pyruvic transaminase)
ALP	Alkaline phosphatase
ALT (SGPT)	Alanine aminotransferase
ANC	Absolute neutrophil count
ANOVA	Analysis of variance
APTT	Activated partial thromboplastin time
ASAT (SGOT)	Asparagine aminotransferase (serum glutamic oxaloacetic transaminase)
ASCO	American Society of Clinical Oncology
AST (SGOT)	Aspartate aminotransferase
AUC	Area under the plasma concentration-time curve
bev	Bevacizumab (rHuMab VEGF; Avastin™)
BID	Twice a day
BP	Blood pressure
BSA	Body surface area
CCSQ	Chemotherapy Convenience and Satisfaction Questionnaire
CEA	Carcinoembryonic antigen
CHF	Congestive heart failure
CI	Confidence interval
CL	Clearance
Cmax	Maximum plasma concentration
Cbar	Average concentration at steady state

CNS	Central nervous system
CPT-11	Irinotecan
CPU	Clinical pharmacology unit
CR	Complete response
CRC	Colorectal cancer
CRF	Case report form(s)
CRO	Contract research organisation
CSO	Central Sample Office (Roche Basel)
CT	Computed tomography
CTCAE	Common terminology criteria for adverse events
CVAD	Central venous access device
D5W	Dextrose 5% in water for injection
DACH	1,2-diaminocyclohexane
DFS	Disease-free survival
DLT	Dose limiting toxicity
DNA	Deoxyribonucleic acid
DPD	Dihydropyrimidine dehydrogenase
DSMB	Data and Safety Monitoring Board
DTRs	Deep tendon reflexes
EC50	Plasma concentration associated with half-maximal effect
ECG	Electrocardiogram
ECHO	Echocardiography
ECOG	Eastern cooperative oncology group
EEG	Electroencephalogram
EMEA	European Agency for the Evaluation of Medicinal Products
ESF	Eligibility screening form
EU	European union
FDA	Food and Drug Administration
Flt-1	Fms-like tyrosine kinase

FOLFOX	Chemotherapeutic Regimen consisting of infusional 5-FU, leucovorin and oxaliplatin
GABA	Gamma-aminobutyric acid
GCP	Good clinical practices
G-CSF	Granulocyte colony-stimulating factor
GFR	Glomerular filtration rate
GGT	γ-Glutamyltransferase
GM-CSF	Granulocyte-macrophage colony-stimulating factor
H_0	Null hypothesis
H_1	Alternative hypothesis
HAHA	Human anti-humanized antibody
HCT	Hematocrit
HPLC	High performance liquid chromatography
IB	Investigational brochure
ICH	International Conference on Harmonization
ICU	Intensive care unit
IFL	Irinotecan, bolus 5-FU, leucovorin (Saltz regimen)
IND	Investigational new drug
INR	International normalized ratio (prothrombin ratio)
IRB/IEC	Institutional review board/independent ethics committee
ITT	Intent to treat
IV	Intravenous
IVRS	Interactive voice response system
K10	Elimination rate constant
K12/21	Intercompartment rate constants
KDR	Kinase domain region
keo	Equilibration rate constant
LDH	Lactate dehydrogenase
LFT	Liver function test (s)

LV	Leucovorin
mCRC	Metastatic colorectal cancer
MCU	Medical Care Utilization
mg	Milligram
mL	Milliliter
MRI	Magnetic resonance imaging
MTD	Maximum tolerated dose
MUGA	Multiple ECG-gated radionuclide angiography
NCI	National cancer institute
NCI CTC	National cancer institute common toxicity criteria
NCI CTCAE	National Cancer Institute Common Terminology Criteria for Adverse Events
NOEL	No observable effect level
NOAEL	No observable adverse effect level
NSABP	National Surgical Adjuvant Breast and Bowel Project
NYHA	New york heart association
OR	Overall response
ORR	Objective response rate
OS	Overall survival
PD	Progressive disease
PD	Pharmacodynamic
PFS	Progression free survival
PK	Pharmacokinetic
PP	Per protocol
PR	Partial response
PS	Performance status
Pt	Platinum
PVC	Polyvinyl chloride
Q12H	Very 12 hours

Q3W	Every 3 weeks
Q2W	Every 2 weeks
QW	Once a week
RFS	Relapse-free survival
RIA	Radio immunoassay
SAE	Serious adverse event
SD	Stable disease
SDD	Survival distribution of disease-free survival
SMT	Study management team
SoA	Schedule of Assessment
SWOG	Southwest Oncology Group
$T_{1/2}$	Half-life
TP	Thymidine phosphorylase
TS	Thymidylate synthase
TTP	Time to tumour progression
ULN	Upper limit of normal
US	Ultrasound
USP	U.S. Pharmacopeia
V_c	Volume of central compartment
VEGF	Vascular endothelial growth factor
VEGFR	Vascular endothelial growth factor receptor
vs	versus
XELOX	Chemotherapeutic Regimen consisting of capecitabine and oxaliplatin

PART I: STUDY DESIGN AND CONDUCT

1. BACKGROUND AND RATIONALE

1.1 Colon Cancer

Colorectal cancer is one of the most frequent malignancies, second to breast cancer in women and third to lung cancer and prostate cancer in men [1,2]. The prognosis for the individual patient is dependent upon the extent of the disease. The 5-year survival rate is over 60% in individuals with lymph node involvement but less than 5% in individuals with distant metastatic disease [2,3,4,5]. It is therefore very important that patients with "high-risk" primary colon tumours receive the best possible chance of cure, since metastatic disease is incurable in most patients. Thus, based on the limited chances for a curative treatment of metastatic colorectal cancer, the concept of adjuvant chemotherapy was developed.

5-fluorouracil (5-FU), in use for over 40 years, is the most active single agent against metastatic colorectal cancer, though response rates are usually less than 20% [2,3,6]. In locally advanced and distant metastatic disease 5-FU based chemotherapy improves median survival by 4-6 months compared with best supportive care. 5-FU in combination with leucovorin (5-FU/LV) became the standard chemotherapy for colorectal cancer in the 1980's and was associated with median survival of 11 months. Given by repeated bolus injections or short infusions it is, however, associated with toxicity, in particular gastro-intestinal (including diarrhea and stomatitis), neurological and myelosuppressive toxicity, which limit the intensity and duration of treatment [7]. Trials have reported increased response rates with low dose continuous 5-FU infusion as first line chemotherapy for metastatic colorectal cancer [7,8,9,10]. While less severe acute toxicities are observed with the low dose continuous schedules, these are not eliminated, and other toxicities associated with chronic exposure of tissues to 5-FU or its metabolites become apparent, such as 'hand-foot syndrome' [7,8,9,10]. However, problems associated with a permanent indwelling catheter such as the risk of infection and thromboembolism, as well as administration problems, inconvenience and costs, do not favor protracted infusion.

Two new drugs, the topoisomerase I inhibitor irinotecan (CPT-11) and the platinum compound oxaliplatin, initially showed efficacy as second-line therapy of metastatic disease when used as monotherapy and in combination with 5-FU. More recently phase III trial results were reported, showing superiority of both drugs as first-line treatment in combination with 5-FU/leucovorin (LV), compared to 5-FU/LV therapy alone [11,12,13].

1.2 Adjuvant Colon Cancer Therapy

The Intergroup trial (INT-0035) was the first large-scale study to demonstrate a significant effect of a post-operative adjuvant treatment in patients with Dukes stage C (stage III; pTany N+ M0) colon cancer. This trial randomized 1296 patients with stage II and III cancer (929 with stage III cancer) to one of three arms: (a) surgery alone, (b) surgery plus 12 months of levamisole, or (c) surgery plus 12 months of 5-FU plus levamisole [14,15]. The study showed a 15% absolute reduction (40% relative reduction) in the risk of recurrence and a 16% absolute reduction (33% relative reduction) in the

overall death rate in patients with stage III colon cancer treated with the combination of surgery plus 5-FU/levamisole. The Canadian and European consortium trial (IMPACT) compared adjuvant treatment with high-dose 5-FU and LV with no treatment in nearly 1500 patients, demonstrating a 22% relative risk reduction in mortality at 3 years in patients with colon cancer, Dukes stage C [16]. In a further study utilizing 6 months of treatment, the *Mayo Clinic regimen* (Table 1) was shown to significantly improve time to relapse and survival versus observation alone [17]. In a large randomized study by the North Central Cancer Treatment Group (NCCTG) and the National Cancer Institute of Canada (NCIC), it was shown that there was no additional benefit associated with administration of 12 months chemotherapy compared with just 6 months of treatment and underlined the efficacy of LV in the adjuvant setting [18]. The Intergroup study INT-0089 demonstrated equi-efficacy of the modified *Roswell Park* (Table 1) and *Mayo Clinic* regimens [19].

A recently published consensus emphasized adjuvant treatment in stage III colon cancer ($pT_{any}N+M0$; Dukes stage C) with two bolus 5-FU/LV regimen (*Mayo Clinic* regimen and *Roswell Park* regimen) as standard options [20]. However, it was noted that infusional 5-FU/LV regimens have to be considered in the adjuvant treatment based on the results from ongoing clinical trials.

Demonstration of the efficacy of infusional 5-FU/LV *de Gramont* regimen (Table 1) compared to *Mayo Clinic* regimen was an outcome of a randomized comparison in advanced colorectal cancer [21]. The infusional regimen had a superior response rate (33% vs 14%) and progression-free survival (28 vs 22 weeks). However, no significant difference in terms of overall survival (62 weeks and 57 weeks, respectively, p = 0.07) could be shown. The *de Gramont* regimen has already been applied in the adjuvant setting [22]. The study included 905 patients with Dukes B2 and C colon cancer treated with either 5-FU/LV (de Gramont regimen) or bolus 5FU/LV (5FU-LV hd; [Table 1]). Despite of a lack of a statistical improvement for disease-free survival by the *de Gramont* regimen it became an accepted standard due to the improved safety profile.

Table 1 Schedules of 5-FU/LV in adjuvant colon cancer regimens

Name of regimen	Schedule
Mayo Clinic [18]	5-FU 425 mg/m^2 i.v. bolus +<br>LV 20 mg/m^2 i.v. bolus<br>d1-5, q 4 weeks x 2, then q 5 weeks x 4
modified Roswell Park [20]	LV 500 mg/m^2 2-hour infusion +<br>5-FU 500 mg/m^2 i.v. bolus 1 hour after the start of the LV infusion<br>weekly for weeks 1-6, q 8 weeks x 4
de Gramont or LV5FU2 [26]	LV 200 mg/m^2 2-hour infusion +<br>i.v. bolus 5-FU 400mg/m^2 and 22 hours continuous infusion 600 mg/m^2,<br>d1 and d2, q 2 weeks x 12
5FU-LV hd [24]	LV 200 mg/m^2 15 minute i.v. infusion +<br>5-FU 400 mg/m^2 15 minute infusion,<br>d1-5 q 4 weeks x 6

The treatment of adjuvant colon cancer changed significantly based on the results of the MOSAIC trial using the *de Gramont* regimen with or without oxaliplatin reported at ASCO 2003. The 3-year disease-free survival for the ITT population was significantly increased in the FOLFOX-4 arm compared to the control arm (78.2% versus 72.9%) reflecting a risk reduction for recurrence by 23% [23]. This benefit was observed in all sub-groups.

The NSABP C-07 trial randomized patients to either the modified Roswell Park regimen alone, or a combination of oxaliplatin and the Roswell Park regimen. The data of the C-07 study are pending.

Furthermore, two randomized cooperative group trials evaluate irinotecan in adjuvant treatment. Irinotecan was investigated in the CALGB 89803 study based on the IFL regimen (irinotecan + bolus 5-FU/LV) versus the *Roswell Park* regimen. This study has not yet been presented but it has been communicated that, based on a futility analysis, the primary objective will not be reached (Saltz L. Letter to National Cancer Institute Cooperative Group Investigators, August 26, 2003).

PETACC-3 (Pan-European Trials in Adjuvant Colon Cancer) trial compares the *Douillard* regimen (irinotecan + infusional 5-FU/LV) with the *de Gramont* regimen.

1.3 Bevacizumab (rHuMAb VEGF)

1.3.1 Vascular Endothelial Growth Factor (VEGF)

Of the identified angiogenic factors, vascular endothelial growth factor (VEGF; also known as vascular permeability factor) is the most potent and specific and has been identified as a crucial regulator of both normal and pathologic angiogenesis [27]. VEGF is a highly conserved, homodimeric, secreted, heparin-binding glycoprotein whose dominant isoform has a molecular weight of 45,000 Daltons [26,27]). VEGF produces a

number of biologic effects, including endothelial cell mitogenesis and migration, induction of proteinases leading to remodelling of the extracellular matrix, increased vascular permeability and maintenance of survival for newly formed blood vessels [27]. VEGF expression is regulated by hypoxia via molecular pathways similar to those regulating erythropoietin gene expression [27]. The biologic effects of VEGF are mediated through binding and stimulation of two receptors on the surface of endothelial cells: Flt-1 (fms-like tyrosine kinase) and KDR (kinase domain region) [27]. Increased levels of VEGF expression have been found in most human tumours examined to date, including tumours of the lung, breast, thyroid, gastro-intestinal tract, kidney, bladder, ovary, and cervix, as well as angiosarcomas and glioblastomas [27]. Specifically in colorectal cancer, increased VEGF expression correlates with invasiveness, vascular density, metastasis, recurrence, and prognosis [38,40,41,42,43]. In a nude mouse model of hepatic metastases, antibodies to VEGF decreased the number and size of hepatic metastases [43]. In addition, levels of VEGF in the ascites of patients with metastatic colorectal cancer are markedly elevated, suggesting that VEGF-induced vascular permeability may contribute to the formation of malignant ascites [44]. In *in-vitro* investigations it was shown that VEGF can prevent the functional maturation of dendritic cells. Thus, it was speculated that VEGF plays an important role in the suppression of the antitumoural immune response [45].

1.3.2 Anti-VEGF Monoclonal Antibody - rHuMAb VEGF

Inhibition of VEGF using an anti-VEGF monoclonal antibody blocks the growth of a number of human cancer cell lines including colorectal cancer in nude mice [27]. In addition, the combination of anti-VEGF antibody and chemotherapy in nude mice injected with human cancer xenografts resulted in an increased antitumour effect compared with antibody or chemotherapy treatment alone [24]. To test the hypothesis that inhibition of VEGF in patients with cancer results in clinical benefit, a recombinant humanized version of a murine anti-human VEGF monoclonal antibody, named rhuMAb VEGF (bevacizumab), was created [37]. Single agent activity of bevacizumab was seen in different xenograft models [85, 86]. Bevacizumab has been brought into clinical development for use as a single agent to induce tumour shrinkage in patients with solid tumours and for use in combination with cytotoxic chemotherapy to delay the time to disease progression in patients with metastatic solid tumours.

Recently, direct anti-vascular effects of bevacizumab were observed in rectal cancer patients confirming former *in-vitro* effects [82]. In a neo-adjuvant phase I treatment protocol, patients received a single administration of 5 mg/kg bevacizumab followed by endoscopic tumour assessment and subsequent multi-modal treatment with bevacizumab, 5-FU and external beam radiation after 14 days. After a single administration of bevacizumab a decreased tumour perfusion, vascular volume, microvascular density, and interstitial fluid pressure were observed. Furthermore, an effect on circulating endothelial and progenitor cells was demonstrated. In one out of 6 patients with rectal cancer a tumour shrinkage >30% was observed after a single administration.

1.3.3 Preclinical Safety Studies – Bevacizumab

Treatment-related effects observed in the preclinical safety studies were consistent with the intended pharmacological activity of bevacizumab to inhibit VEGF-dependent angiogenesis and may therefore occur at clinically relevant exposure levels.

In cynomolgus monkey studies, once or twice weekly IV treatments with bevacizumab (doses of 2, 10, and 50 mg/kg) for either 4, 13, or 26 weeks were well tolerated [25, 39]. There were no effects on body weight, food consumption, blood pressure, electrocardiograms, rectal body temperature, respiratory rate, ophthalmologic observations (including electroretinograms), or clinical pathology parameters. In all active treatment groups, animals with open growth plates showed physeal dysplasia with focal to diffuse chondroid necrosis and linear fissuring of the cartilaginous growth plate. The incidence and severity of physeal dysplasia increased in relation to dose and duration of treatment. The NOEL/NOAEL for this finding was not determined and effects occurred at average exposure levels <1-fold the average clinical exposure levels. In all studies, evidence of reversibility was noted. It should be noted, however that this effect occurred only in actively growing animals with open growth plates. Because bevacizumab will most likely be administered to adult patients with closed growth plates, physeal dysplasia is not expected to occur in the clinical population. In addition, female rats treated with 10 or 50 mg/kg once or twice weekly for 13 or 26 weeks had decreased ovarian function and corresponding effects on the reproductive cycle; i.e. decreased number/absence of corpora lutea and inhibition of follicular maturation at the early Graafian follicle stage, decreased ovarian and uterine weights, reduction of endometrial proliferation and decrease in the mean number of menstrual cycles. These findings were shown to be at least partially reversible upon cessation of treatment. These findings were expected considering the known role of VEGF-dependent angiogenesis in formation of the corpora lutea and of the growing bone [28]. VEGF is also known to be involved in the process of wound healing [45,46,47]. To assess the effects of bevacizumab on wound healing, partial thickness circular skin wounds were induced in rabbits treated with bevacizumab every two to four days for 2 weeks at doses from 2 to 50 mg/kg [33]. Dose-related inhibition of wound healing was exhibited following treatment with bevacizumab, however complete healing occurred after cessation of dosing. These findings were confirmed in a full-thickness linear incision model in the rabbit. In this model bevacizumab was administered every other day for 5 days (total of 3 doses) at 0.5, 1, or 2 mg/kg and this resulted in a dose dependent and significant decrease in the tensile strength of the wounds. The exposure levels at the lowest dose tested were approximately 0.1-fold the average clinical exposure levels.

In the cynomolgus monkeys, antibodies against bevacizumab were only detected in two animals; one control animal at study day 15 and one high-dose animal on study day 183 of the 26-week toxicity study. However, these responses were very weak, i.e. just slightly above the minimal detectable level of the available assay, and no effects on toxicokinetics or toxicity profiles were observed in the high-dose animal.

Bevacizumab has been shown to be embryotoxic and teratogenic when administered to rabbits. Observed effects included decreases in maternal and foetal body weights, an increased number of foetal resorptions and an increased incidence of specific gross and

skeletal foetal alterations. Adverse foetal outcomes were observed at all tested doses of 10–100mg/kg and the foetal NOEL/NOAEL could not be determined. Exposure levels at the lowest dose tested (10 mg/kg) were approx. 3-fold the average clinical exposure levels.

1.3.4 Clinical Studies – Bevacizumab

1.3.4.1 Clinical Pharmacology – Bevacizumab

Clinical pharmacokinetics was primary assessed in phase I and phase II studies as single agent or in combination with cytotoxic chemotherapy in several tumour types and supported by population pharmacokinetics including the pivotal study AVF2107g.

At doses of ≥1 mg/kg, bevacizumab disposition was similar in these clinical trials. Bevacizumab was characterized by a slow clearance of ~3 mL/kg/day and a terminal elimination half-life of ~20 days. Co-administration of bevacizumab with cytotoxic chemotherapy did not appear to result in a change in the systemic concentrations of the cytotoxic agents [34].

Body weight and sex were the covariates determined to be the most significant in explaining inter-patient variability in CL and V_c [34]. Patients with higher body weight had a faster CL and a larger V_c compared to patients with lower body weight. Patients with higher body weight received higher doses of bevacizumab, which compensated for the faster CL and V_c. Median trough levels measured in study AVF2107g supported the use of weight-based dosing. Furthermore, population PK analysis in this study demonstrated that bevacizumab CL was similar when administered in combination with IFL chemotherapy compared to as a single agent [35].

Bevacizumab CL was ~20% higher in patients with low serum albumin (≤ 29 g/L), and ~20% higher in patients with elevated alkaline phosphatase (≥ 483 U/L), relative to patients with median values. Given the modest inter-patient variability in CL and V_c and the weight-based dosing regimen, all other identified covariates had only minor effects on bevacizumab PK parameters. Considering the compelling survival benefit seen consistently across all predefined subgroups in the pivotal trial AVF2107g [36] and the supportive trial AVF0780g [3] it is believed that these findings are not of clinical relevance.

As a reference to patient's convenience it is important to link the administration of bevacizumab with the infusion of chemotherapeutic drugs despite different cycle durations for FOLFOX and XELOX. In the pivotal study for first-line treatment of metastatic CRC, AVF2107g, bevacizumab was administered every 2 weeks at 5 mg/kg. The recently accepted new standard treatment FOLFOX has a cycle duration of 2 weeks as well. In contrast, the cycle duration of XELOX is 3 weeks. Both regimen are currently under investigation in first-line metastatic CRC in combination with bevacizumab. Thus, bevacizumab is administered based on the weekly dose equivalent of 2.5 mg/kg i.e. at 5 mg/kg every 2 weeks in combination with FOLFOX or 7.5 mg/kg every 3 weeks in combination with XELOX. This is supported based on simulations on the results of the population PK analysis including data from 491 subjects. The parameters used for simulation were: Vc= 2.66 L, k10=0.0778 day-1, k12=0.223 day-1 and k21=0.215 day-1

and represent the pharmacokinetic parameter for the typical female subject. According to the simulation, the average concentration (cbar) will be very similar and will also result in similar trough concentrations (Table 2). Figure 1 shows the predicted bevacizumab concentration-time profile for a typical female patient receiving 2.5 mg/kg/week every 2 or 3 weeks.

Table 2 Predicted Bevacizumab Pharmacokinetic Parameters at Steady-state

PK Parameter	Dosing Regimen	
	5 mg/kg q 2 weeks	7.5 mg/kg q 3 weeks
Cbar (µg/mL)	121	120
Cmin ss (µg/mL)	85	73
Cmax ss (µg/mL)	215	270
AUCss (µg/mL*days/cycle)	1697	2529
AUCss (µg/mL*days/week)	848,5	843

Where AUCss was calculated using the trapezoidal rule and cbar was calculated as AUCss/τ

At steady-state, the average concentration (cbar) will be very similar for both regimens while the maximum and minimum concentrations will be different by only approximately 20%.

Figure 1 Predicted Bevacizumab Concentrations When Given Every 2 or 3 Weeks

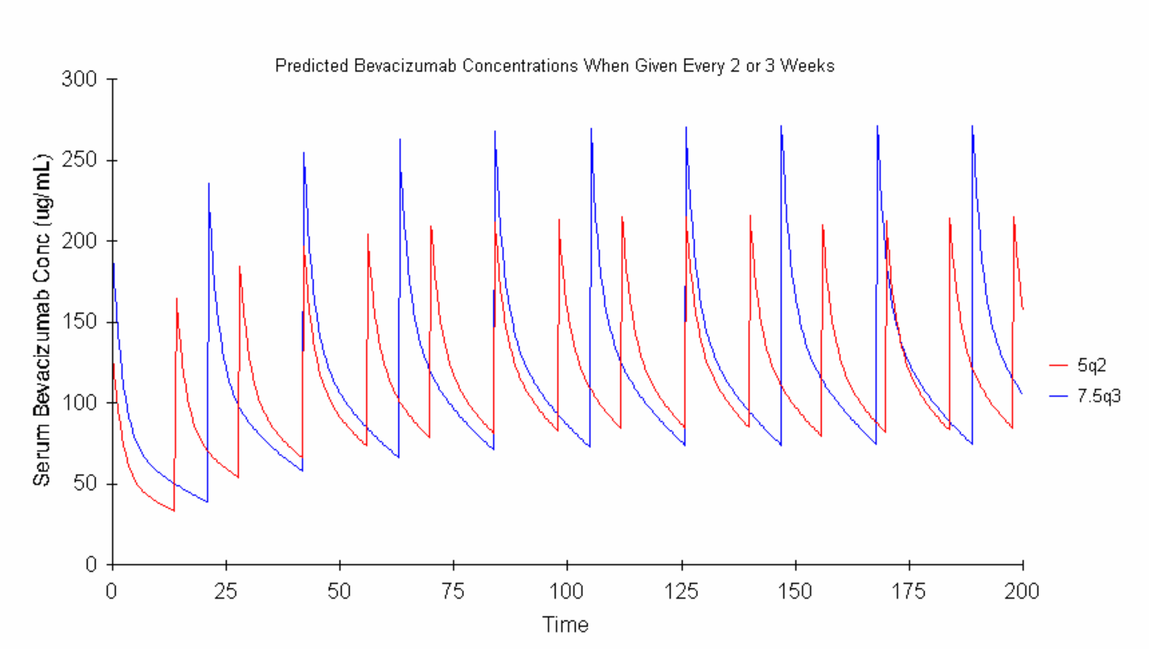

The early studies AVF0780g (mCRC; 5 mg/kg q2w) and AVF0757g (NSCLC; 7.5 mg/kg q3w) compared different doses of bevacizumab at the respective dosing interval in a randomized fashion in combination with either 5-FU/LV or carboplatin/paclitaxel for metastatic colorectal cancer and NSCLC, respectively (Table 3). The PK data of these studies confirmed the simulations based on population PK (Table 2).

Table 3 Bevacizumab Minimum and Maximum Concentrations at Steady-state in Clinical Trials

PK Parameter	Dosing Regimen	
	5 mg/kg q 2 weeks (AVF0780g)	7.5 mg/kg q 3 weeks (AVF0757g)
$Cmin_{ss}$ (µg/mL)	78.7 (34.1)	73.4 (43.2)
$Cmax_{ss}$ (µg/mL)	206 (60.7)	267 (55.2)

$Cmin_{ss}$ and $Cmax_{ss}$ are the mean (SD) at Day 98 and 105 for AV0780g and AVF0757g, respectively

1.3.4.2 Clinical Studies in Colorectal Cancer - Bevacizumab

First results in metastatic CRC were reported in a randomized phase II study (AVF0780g [52], Table 4), evaluating the efficacy, safety and pharmacokinetics of bevacizumab combined with 5-FU/LV in patients with metastatic colorectal cancer. All patients received 5FU/LV given as the Roswell Park regimen. Patients were randomized to three treatment arms: control (5-FU/Leucovorin alone), 5 mg/kg q2w bevacizumab plus 5-FU/Leucovorin, or 10mg/kg q3w bevacizumab plus 5-FU/Leucovorin. Bevacizumab was administered IV every 2 weeks until disease progression or for a maximum of 1 year. The primary efficacy endpoints were time to disease progression and best response rate. Secondary endpoints included survival and duration of response. One hundred and four patients with previously untreated metastatic colorectal cancer were enrolled and randomized to the three treatment arms (36 to the control arm, 35 to the 5 mg/kg bevacizumab arm, and 33 to the 10mg/kg bevacizumab arm).

The response rates were 17% (95%CI 7-34%) in the control arm, 40% (95% CI 24-58%) in the 5 mg/kg bevacizumab arm and, 24% (95% CI 12-43%) in the 10mg/kg bevacizumab arm. The median time to disease progression was 5.2 months in the control arm, 9.0 months in the 5 mg/kg bevacizumab arm, and 7.2 months in the 10mg/kg bevacizumab arm. The median survival rate was 13.8 months in the control arm, 21.5 months in the 5 mg/kg bevacizumab arm, and 16.1 months in the 10 mg/kg bevacizumab arm.

Table 4 Efficacy Results in the Phase II Metastatic CRC Study AVF0780g

Endpoint	Control (N = 36)	Bevacizumab 5 mg/kg/2wk (N = 35)	Bevacizumab 10 mg/kg/2wk (N = 33)
**Time to disease progression (IRF)**			
Number of progressions	26 (72%)	22 (63%)	23 (70%)
Median (months)	5.2	9.0	7.2
Hazard ratio	-	0.440	0.692
p-value (log-rank)	-	0.005	0.217
**Objective response rate (IRF)**			
Objective response	6 (17%)	14 (40%)	8 (24%)
p-value (χ^2)	-	0.029	0.434
Complete response	0	2 (6%)	0
**Duration of survival**			
Number of deaths	19 (53%)	12 (34%)	19 (58%)
Median (months)	13.6	17.7	15.2
Hazard ratio	-	0.521	1.009
p-value (log-rank)	-	0.073	0.978

Note: Independent review facility (IRF)/investigator endpoints were based on the IRF assessment in all but 3 patients; in these patients, the investigator assessment was used.

These phase II results led to a phase III trial that investigated the efficacy and safety of bevacizumab in combination with irinotecan plus bolus 5-FU/LV (IFL, also known as Saltz regimen) as first line therapy for metastatic colorectal cancer [29]. *Eight hundred fifteen* patients were randomized to receive IFL/ placebo or IFL/ bevacizumab (5 mg/kg q2w). A third group of patients (~ 100) received 5-FU/Leucovorin chemotherapy plus bevacizumab. The median survival was 15.6 months in the IFL arm and 20.3 months in the IFL/ bevacizumab arm (p-value = 0.00003). The progression-free survival was 6.24 months in the IFL arm and 10.6 months in the IFL/ bevacizumab arm (p-value <0.00001). The overall response rate was 35% in the IFL arm vs 45% in the IFL/ bevacizumab arm (p-value =0.0029). The duration of response was 7.1 months in the control arm vs 10.4 months in the IFL/ bevacizumab arm (p-value =0.0014). The efficacy results of arm three have been published recently [31]. In the third arm the median survival was 18.3 months, the progression free survival was 8.8 months. The overall response rate was 40% and the duration of response was 8.5 months. This phase III trial demonstrated that addition of bevacizumab to irinotecan plus 5-FU/LV as first line chemotherapy for metastatic colorectal cancer results in clinically meaningful and statistically significant improvement in disease-free survival and overall survival.

An additional study enrolling patients who were not optimal candidates for first-line metastatic CRC treatment with an irinotecan containing regimen (AVF2192g) supported the notion that bevacizumab is an ideal candidate for combination with fluoropyrimidines. This phase II study showed a 67 % prolongation in PFS, which was highly statistically significant. The study also showed a 29 % improvement in survival in

patients who received bevacizumab in combination with chemotherapy compared to those receiving chemotherapy alone. The improvement in overall survival did not achieve statistical significance due to ambitious statistical assumptions. Detailed results will be presented at annual ASCO meeting 2004 [32].

In addition to testing bevacizumab in combination with the IFL regimen a cooperative group study investigated the efficacy and safety of bevacizumab in combination with FOLFOX-4. Patients progressing after treatment with a fluoropyrimidine and an irinotecan based regimen used either alone or in combination were eligible for this trial. An interim safety analysis of the 3-arm ECOG study E3200 consisting of FOLFOX-4, FOLFOX-4 + bevacizumab (10 mg/kg, q2w) and bevacizumab alone (10 mg/kg, q2w) was presented at ASCO GI 2004 [30]. Based on a planned interim analysis, the 3rd arm with single agent bevacizumab treatment was closed prematurely due to survival that appeared to be inferior to the two FOLFOX arms. The safety data presented included information on 757 enrolled patients (Table 5). The conclusion from the ECOG investigators was that addition of bevacizumab to FOLFOX-4 does not substantially alter the toxicity profile of the combination. However, it was noted that the data support the association of bevacizumab with a small incidence of bleeding and hypertension. From the available data set it was not evident that there was an increased risk of thromboembolic events by adding bevacizumab to FOLFOX-4 according to the investigators.

Table 5 ECOG study E3200 interim safety analysis – Grade 3/4 adverse events

	FOLFOX-4 + bevacizumab (10 mg/kg, q2w)		FOLFOX-4		Bevacizumab (10 mg/kg, q2w)	
	N=262		N=265		N=230	
	G3	G4	G3	G4	G3	G4
Hemorrhage	2%	0	0	0	2%	0
Thrombosis/embolism	3%	0	1%	2%	0	<1%
Hypertension	5%	1%	2%	<1%	6%	0
Neutropenia	2%	13%	1%	17%	0	<1%
Febrile neutropenia	3%	0	2%	0	0	0
Infection w/neutropenia	2%	<1%	3%	1%	0	0
Diarrhea	13%	1%	12%	<1%	2%	0
Vomiting	8%	2%	3%	<1%	3%	0
Neuropathy (sensory)	11%	1%	5%	<1%	0	<1%
Fatigue	14%	2%	11%	1%	3%	<1%
Proteinuria	<1%	0	0	0	<1%	0
Any Grade 3 or 4 AE (worst Grade)	44%	21%	32%	25%	25%	9%

Bevacizumab in combination with IFL has shown a significant increase of survival and time to disease progression in first-line CRC [29]. Furthermore, the safety of bevacizumab in combination with FOLFOX-4 was shown in second-line CRC [30]. Therefore, the study NO16966 comparing FOLFOX-4 with XELOX regimen was amended to investigate whether bevacizumab at the recommended dose for CRC in combination with FOLFOX-4 or the XELOX regimen is superior in terms of efficacy compared to chemotherapy alone in first-line CRC. .

Based on the data from the pivotal study AVF2107g, bevacizumab has been approved on February 26, 2004 in the United States as first-line treatment in combination with intravenous 5FU-based regimens for patients with metastatic colorectal cancer.

The cooperative group NSABP has now designed a comparable study protocol for the adjuvant treatment of colon cancer patients with bevacizumab. Study C-08 is a randomized, open-label 2-arm study comparing mFOLFOX-6 with mFOLFOX-6 plus 12 months of bevacizumab. Stage II/III patients will be eligible for this study. The protocol was recently submitted for Special Protocol Assessment to the *Food and Drug Administration* and the approval was granted. The recruitment will start soon.

1.4 Capecitabine - Xeloda®

Capecitabine (Xeloda®) is an oral fluoropyrimidine carbamate rationally designed to generate 5-FU preferentially in tumour tissue through exploitation of high intratumoural concentrations of thymidine phosphorylase (TP), an enzyme present at significantly increased concentrations in a wide range of tumour types, including colorectal, breast and gastric cancers, compared with normal tissue [53].

Human pharmacokinetic studies have shown that after oral administration, capecitabine is rapidly and almost completely absorbed through the gastro-intestinal wall, thus avoiding direct intestinal exposure to 5-FU. Capecitabine is then metabolized to 5-FU via a three-step enzymatic cascade, with the final stage of this conversion mediated by TP [54]. In a second three-step sequence, 5-FU is catabolized to FUH_2 by the enzyme dihydropyrimidine dehydrogenase (DPD) and then to FUPA and FBAL, none of which have any antiproliferative activity.

The preferential activation of capecitabine to 5-FU in tumour, as compared to normal tissue, was demonstrated in a study in patients with colorectal cancer [for details please refer to *Investigators Brochure Capecitabine*].

1.4.1 Clinical Pharmacokinetics and Metabolism of Capecitabine

The clinical pharmacokinetics of capecitabine have been extensively investigated [54]. For details please refer to *Investigators Brochure Capecitabine*.

<u>*Effects of Age and Gender*</u>: Population pharmacokinetic analysis demonstrated that gender had no clinically relevant effect on the pharmacokinetics of capecitabine or its metabolites [56]. Similarly, age had no influence on the pharmacokinetics of 5'-DFUR

or 5-FU. The AUC of FBAL increased with age, an effect probably caused by a change in renal function in the elderly (see Section 7.5.2).

Effects of Hepatic Impairment: Capecitabine (1250 mg/m^2 as a single dose) has been evaluated in patients with mild to moderate hepatic dysfunction caused by liver metastases [57]. No significant differences in the pharmacokinetic parameters of the main metabolites (5'-DFUR, 5-FU and FBAL) were seen in this group compared to patients with normal hepatic function.

Effects of Renal Impairment: Based on a pharmacokinetic study in patients with cancer and mild to severe renal impairment treated with capecitabine 1250 mg/m^2 BID (monotherapy), there is no evidence for an effect of creatinine clearance on the pharmacokinetics of intact drug and 5-FU. However, creatinine clearance was found to influence the systemic exposure to 5'-DFUR (35% increase in AUC when creatinine clearance decreases by 50%) and to FBAL (114% increase in AUC when creatinine clearance decreases by 50%) [37]. 5'-DFUR is the direct precursor of 5-FU and FBAL is a metabolite without antiproliferative activity (see Section 7.5.2).

1.4.2 Clinical Studies of Capecitabine Monotherapy in Metastatic Colorectal Cancer

Two multicenter, open-label, phase III studies were conducted to compare the intermittent regimen of capecitabine with i.v. bolus 5-FU/LV (Mayo Clinic regimen, the regulatory standard at the time), as first-line treatment for metastatic colorectal cancer [60, 61]. The studies used identical protocols and an integrated analysis of all data was prospectively planned [62]. A total of 1207 patients were randomized to either oral capecitabine (1250 mg/m^2 twice daily, days 1–14 every 21 days; n=603) or the Mayo Clinic regimen (n=604). Capecitabine demonstrated a significantly superior response rate compared with 5-FU/LV (26% versus 17%; p<0.0002). Median time to response, time to treatment failure and duration of response were all similar in the two treatment groups. In addition, time to disease progression (TTP) was equivalent in the two treatment groups (median 4.6 versus 4.7 months with capecitabine and 5-FU/LV, respectively). Overall survival was also equivalent in the two treatment groups (median 12.9 versus 12.8 months, respectively). Subgroup analysis demonstrated that capecitabine treatment consistently resulted in superior response rates (p<0.05), even in patient subgroups with poor prognostic indicators. Multivariate Cox regression analysis identified poor performance status (KPS ≤80 vs 100), liver as a dominant site of metastasis and multiple vs single metastases as independent prognostic indicators for poor survival [62].

The clinical safety profile of capecitabine is similar to other fluoropyrimidines. Important differences, however, are noted. In these monotherapy studies, compared to patients treated with 5-FU/LV, capecitabine treated patients experienced a significantly lower incidence of treatment-related (all grades) diarrhea (47.7 vs 58.2%), stomatitis (24.3 vs 61.6%), nausea (37.9 vs 47.6%), alopecia (6.0 vs 20.6%); and Grade 3 or 4 neutropenia (2.3 vs 22.8%). A similar incidence of Grade 3 or 4 diarrhea (13.1 vs 12.2%) and a higher incidence of Grade 3 hand-foot syndrome (17.1 vs 1%) were observed. Grade 3 (NCIC; 1.5 to 3.0 x ULN) and 4 hyperbilirubinemia were reported in 18.3% and 4.5% of patients taking capecitabine, compared to 3.3% and 2.5% of patients treated with 5-FU/LV,

respectively. Episodes of Grade 3 or 4 hyperbilirubinemia were usually isolated abnormalities and not associated with other signs of liver morbidity [63].

The incidence of hospitalization due to treatment-related adverse events was 11.6% with capecitabine vs 18% with 5-FU/LV (p=0.002). The incidence of hospitalizations due to stomatitis (0.2 vs 3.5%) and neutropenic fever and sepsis (0.2 vs 2.9%) was significantly less in patients taking capecitabine. Hand-foot syndrome (HFS) required hospitalization (for less than 24 hours) in only two patients taking capecitabine. Dose modifications for toxicity occurred less frequently (33.9 vs. 42.2%) and later after initiation of therapy (2.5 months vs 1.2 months) with capecitabine compared with 5-FU/LV treatment [62,63].

Capecitabine has been registered globally as first-line monotherapy for patients with metastatic colorectal cancer.

1.4.3 Clinical Studies of Capecitabine Monotherapy in Adjuvant Colon Cancer

Beginning in 1998, a phase III study has been conducted aiming to randomize 1956 patients after surgery for colon cancer, Dukes stage C, to either 6 months of treatment with oral capecitabine (1250 mg/m^2 twice daily, days 1–14, every 21 days) or the Mayo Clinic regimen. Accrual was completed in the fall of 2001 and all patients have now completed the study chemotherapy treatment. An assessment of safety of the study chemotherapy treatment has recently been published [64]. In the safety population (capecitabine n=993, bolus 5-FU/LV n=974), patients receiving capecitabine experienced significantly (p<0.001) less diarrhea, stomatitis, nausea/vomiting, alopecia, and neutropenia but more hand-foot syndrome than those receiving 5-FU/LV. Fewer patients receiving capecitabine experienced Grade 3 or 4 neutropenia, febrile neutropenia/sepsis, and stomatitis (p<0.001), although more experienced Grade 3 hand-foot syndrome than those treated with 5-FU/LV (p<0.001). Capecitabine demonstrates a similar, favorable safety profile in patients aged <65 years or ≥ 65 years. Capecitabine has been shown to be at least equivalent to bolus 5-FU/LV (the Mayo Clinic regimen) with respect to disease-free survival [HR 0.87 (95 % CI 0.75 – 1.00)], the primary objective of the study being met [91].

1.5 Oxaliplatin - Eloxatin®

Oxaliplatin (Eloxatin®) is a platinum derivative in which the platinum atom is complexed with a 1,2 diaminocyclohexane (DACH) and with an oxalate ligand as a leaving group. It was synthesized with the goal of trying to overcome resistance to first- and second generation platinum compounds [65].

The mechanism of action of oxaliplatin is similar to that of cisplatin as well as other platinum (Pt) compounds. Studies conducted to date indicated that the types and percentages of Pt-DNA adducts formed by oxaliplatin were qualitatively similar to those formed by cisplatin, but preclinical data suggested several unique attributes of the cytotoxic/antitumour activity of oxaliplatin. Oxaliplatin demonstrated a broad spectrum of in vitro cytotoxic and in vivo antitumour activity that differed from that of either cisplatin or carboplatin. Oxaliplatin was active against several cisplatin-resistant cell lines, colon carcinoma, and other solid tumours that were not responsive to cisplatin. In

addition, oxaliplatin in combination with 5-FU led to synergistic antiproliferative activity in several in vivo tumour models [66].

1.5.1 Clinical Studies of Oxaliplatin Monotherapy in Colorectal Cancer

Oxaliplatin has been widely studied and is active as monotherapy in front-line or subsequent therapy settings in patients with advanced colorectal cancer [66]. Response rates of 20% and 10% in previously untreated and in 5-FU pretreated/refractory patients, respectively, have been observed [67,68]. Adverse events reported in ≥15% of 153 patients given oxaliplatin monotherapy after failure of CPT-11 and 5-FU/LV in a randomized phase III trial included the following: neuropathy (76%), fatigue (61%), nausea (64%), diarrhea (46%), vomiting (37%), abdominal pain (31%), fever (25%), anorexia (20%), anemia (64%), thrombocytopenia (30%), elevated ALAT (36%) and ASAT (54%). Grade 3 or 4 AEs reported in ≥2% of these patients included: fatigue (9%), neuropathy (7%), dyspnea (7%), abdominal pain (7%), nausea (4%), diarrhea (4%), vomiting (4%), dehydration (3%), pain (3%), anorexia (2%), hypokalemia (2%), thrombocytopenia (3%), elevated bilirubin (5%) and ASAT (4%) [54].

1.5.2 Clinical Studies of First-line Oxaliplatin in Combination with 5-FU/LV vs. 5-FU/LV Monotherapy in Colorectal Cancer

Safety and efficacy of the FOLFOX-4 regimen is established [70, 71]. In previously untreated patients receiving the combination of oxaliplatin and 5-FU/LV, the response rates ranged from 34% to 58% with median survival ranging from 15 to 19 months [13, 65]. Furthermore, the efficacy of the combination of oxaliplatin with infusional 5-FU/LV was superior to the combination with a bolus regimen [72, 92, 93].

Oxaliplatin has been approved for use in combination with 5-FU/LV as first-line chemotherapy in most European countries and the US.

1.6 Clinical Studies of Combination Capecitabine and Oxaliplatin (XELOX) in Colorectal Cancer

The safety and efficacy of the XELOX regimen selected for the third arm of the study BO17920 was confirmed in a large phase II trial and appears to have the best therapeutic ratio of all capecitabine-oxaliplatin combinations tested to date [76]. This trial included 96 patients [median age 64 (range 34-79), 64% male] with previously untreated metastatic colorectal cancer. Twenty-seven percent of the patients had received prior adjuvant 5-FU and the majority had liver metastases. The safety profile of XELOX was very similar to that of the FOLFOX-4 regimen in the pivotal phase III trial [13] described earlier. The most common (≥ 5%) treatment-related grade 3 or 4 adverse events (with a total of only 5% grade 4) were diarrhea (16%), sensory neuropathy (17%), and nausea/vomiting (13%). Only 3% grade 3 hand-foot syndrome was reported. There was 7% grade 3 neutropenia, 4% grade 3 thrombocytopenia, and 4% grade 3 hyperbilirubinemia (no grade 4) [76].

A median of 8 cycles of XELOX were administered and patients received a median of 2 additional capecitabine monotherapy cycles after discontinuing oxaliplatin due to neurotoxicity. Sixteen (17%) patients withdrew due to adverse events. One death was

attributed to study treatment: respiratory failure in a patient with pre-existing pulmonary fibrosis. Despite the long treatment duration in this trial, 50% of the patients did not require any dose reductions; 15% required only capecitabine dose reduction, 13% required only oxaliplatin dose reduction and 23% had a reduction of both agents. The response rate was 55% (95% CI 45-65%) with 2% complete responders. In addition, 31% of the patients had stable disease (95% CI 22-42%) lasting >3 months. Median progression free survival in the intent-to-treat population was 7.7 months (95% CI 6.4-8.6 months). Median overall survival was 19.5 months (95% CI 15.3-21.6 months) with a minimum follow-up of 24 months. The survival rate at 1 and 2 years was 70 and 30%, respectively [76].

1.7 Rationale

1.7.1 Rationale for the Study and Study Design

The MOSAIC study demonstrated an increased DFS with the FOLFOX-4 regimen compared with the infusional 5-FU/LV regime alone for the adjuvant treatment of colon cancer and has positioned FOLFOX-4 regimen as the perceived new standard of care. Due to the neurotoxicity of oxaliplatin the consensus achieved in the last decade with bolus 5-FU/LV regimen stating that treatment duration of 6 months represents the standard will not be challenged [18]. This reflects the hypothesis that micrometastases are either destroyed by chemotherapy or avoid damage by the development of resistance during this interval.

Based on the synergism of bevacizumab in combination with chemotherapy observed in studies AVF2107g and AVF2192g in metastatic CRC, it is important to assess if bevacizumab provides a benefit when added to the currently most effective adjuvant treatment, i.e. FOLFOX-4 in patients with colon cancer who underwent definite surgery. Since the study results will not be available before 2009 the design aims to consider potential changes to the regimen used in the MOSAIC study. Thus, the XELOX regimen was chosen for the third arm of the study with capecitabine as an oral fluoropyrimidine.

The MOSAIC study demonstrated a benefit for all stage II colon cancer patients treated with FOLFOX-4. In order to ensure an acceptable risk-benefit ratio for the 12-months adjuvant treatment period within the stage II patient population a sub-group with a high risk profile for relapse was defined.

The work by Willett et al. showing a direct anti-vascular effect of bevacizumab [82] and considerations of potential suppression of an angiogenic switch [84] support an extended treatment with bevacizumab beyond 6 months with chemotherapy. Therefore, the treatment duration of bevacizumab has been chosen to be 12 months. After 6 months combined treatment with chemotherapy bevacizumab will be continued for another 24 weeks as a maintenance therapy to prolong the direct anti-vasculature (pro-apoptotic) effects demonstrated *in-vivo* and in human rectal cancer [43, 85, 86, 82] as a single agent. The addition of bevacizumab for 12 months is considered to be a reasonable balance between duration and feasibility of the treatment. This should guarantee a sustained suppression of an angiogenic switch of micrometastasis in patients who underwent potential curative surgery for colon cancer [84].

In summary, the trial design for BO17920 study combines the concept of direct anti-vascular effects of bevacizumab as a single agent and synergistic effect in combination with chemotherapy. The requirement of a prolonged administration without interruption of inhibitors of angiogenesis was postulated already in the last decade and is in contrast to conventional chemotherapy [90].

1.7.2 Rationale for Dosage Selection

The recommended dose for bevacizumab in colorectal cancer is 5 mg/kg every two weeks. It was identified by a comparison of two dosing regimens (5 mg/kg and 10 mg/kg every two weeks) in combination with the *Roswell Park* regimen in a Phase II study [52] (Table 4). The decision was based on a statistically significant and clinically meaningful prolongation of TTP and an increase in the RR. This dosing regimen was selected for the pivotal Phase III trial AVF2107g.

The development of dosages and cycle length of chemotherapy combinations is based on the safety and efficacy profile, which is determined by pharmacokinetic features of the components. This has led to different cycle durations of the FOLFOX and XELOX regimen. Nevertheless, the administration of bevacizumab has to be aligned with the infusion of chemotherapeutic drugs. Thus, bevacizumab is administered based on the weekly dose equivalent of 2.5 mg/kg i.e. at 5 mg/kg every 2 weeks in combination with FOLFOX or 7.5 mg/kg every 3 weeks in combination with XELOX. This approach is supported by data from population PK analysis. The concept of a weekly dosing equivalent will be confirmed by a PK sub-study in the NO16966 study in metastatic CRC comparing the exposure to bevacizumab for the two dosing regimen.

For reasons of increased convenience due to a reduced number of required visits the administration interval of bevacizumab as a single agent during week 25 to 48 will be 3 weeks (7.5 mg/kg) for patients on arms B and C.

2. OBJECTIVES OF THE STUDY

Primary objectives

- To demonstrate that the combination of bevacizumab and FOLFOX-4 is superior to FOLFOX-4 alone in terms of disease-free survival in chemotherapy-naïve patients who underwent surgery with curative intent for colon carcinoma.
- To demonstrate that the combination of bevacizumab and XELOX is superior to FOLFOX-4 alone in terms of disease-free survival in chemotherapy-naïve patients who underwent surgery with curative intent for colon carcinoma.

Secondary objectives

- To demonstrate that the combination of bevacizumab and FOLFOX-4 is superior to FOLFOX-4 alone in terms of overall survival in chemotherapy-naïve patients who underwent surgery with curative intent for colon carcinoma.
- To demonstrate that the combination of bevacizumab and XELOX is superior to FOLFOX-4 alone in terms of overall survival in chemotherapy-naïve patients who underwent surgery with curative intent for colon carcinoma.

- In case both primary objectives are achieved, to further investigate if the combination of bevacizumab and XELOX is at least as efficacious as the combination of bevacizumab and FOLFOX-4 in terms of disease-free survival and overall survival
- To evaluate and compare the safety profiles of the treatment groups.
- To evaluate the immunogenicity of bevacizumab measured as induction of HAHA.

Tertiary objectives

- To evaluate and compare the perceived convenience and satisfaction with chemotherapy for patients in the three treatment groups.
- To evaluate and compare medical care utilization in the three treatment groups.

3. STUDY DESIGN

3.1 Overview of Study Design and Dosing Regimen

This is an open-label phase III, multicenter, multinational, randomized, 3-arm study designed to evaluate the efficacy and safety of bevacizumab in combination with either intermittent capecitabine plus oxaliplatin ("XELOX") or fluorouracil/leucovorin with oxaliplatin ("FOLFOX-4") versus “FOLFOX-4” regimen alone as adjuvant chemotherapy in colon carcinoma.

The treatment phase consists of two parts of 24 weeks for a total of 48 weeks (Figure 2). The first part consists of treatment with either FOLFOX-4, FOLFOX-4 in combination with bevacizumab, or XELOX in combination with bevacizumab. The 2nd part consists of an observation period for patients assigned to arm A or a treatment period with bevacizumab as a single agent in arms B and C.

The study regimens include (for detail see 6.1):

Arm A: (FOLFOX-4): Oxaliplatin, leucovorin (LV) and 5-fluorouracil (5-FU).

Arm B: (FOLFOX-4+bev): Bevacizumab, oxaliplatin, leucovorin (LV) and 5-fluorouracil (5-FU).

Arm C: (XELOX+bev): Bevacizumab, oxaliplatin in combination with capecitabine.

The cycle duration in arms A and B is 2 weeks with a total of 12 planned cycles. The cycle duration in arm C is 3 weeks and a total of 8 cycles. The cycle duration during week 25 to 48 of bevacizumab as a single agent will be 3 weeks for a total of 8 cycles for patients on arms B and C. In arm A the visit schedule for patients on observation only will be 3 weeks as well.

Patients will be followed for recurrence/new occurrence of colorectal cancer and survival.

Those patients who experience a confirmed recurrence, occurrence of a new colorectal cancer during therapy, or experience unacceptable toxicity will be taken off study treatment and must enter follow-up phase. In case of a confirmed recurrence/appearance of new colorectal cancer patients will be followed for survival until the end of study

follow up period. The primary analysis will be performed after approximately 836 events have occurred in patients with stage III disease, and is expected to be approximately 36 months after the last patient has been randomized. Patients will be further followed for recurrence/new occurrence of colorectal cancer and survival for further 2 years (end of the study follow up). An additional follow-up analysis for efficacy will be performed at that time point (end of the study follow-up).

Figure 2 Overview of Study Schedule and Treatment Order

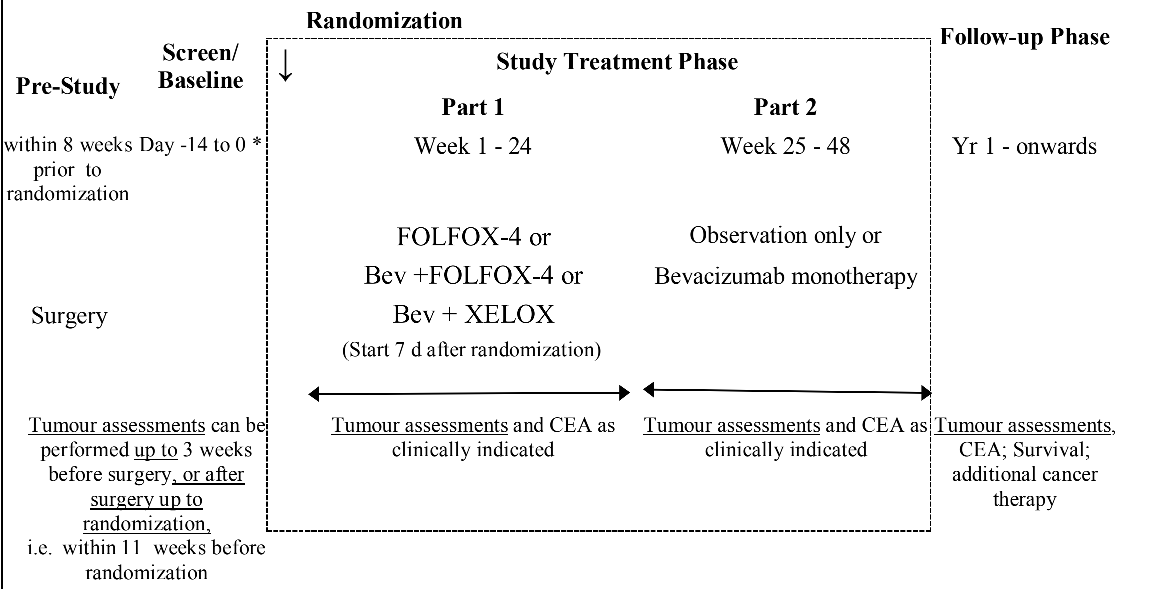

* Days before randomization, including CEA (only exception are tumour assessments which, if already performed in the context of routine patient care, can be performed within 3 weeks before surgery, and surgery can be up to 8 weeks before randomization, for a maximum of 11 weeks between scans and randomization).
Chest X-ray safety assessment is to be performed within 21 days before randomization.

The first 300 patients to be enrolled in the study will be monitored for safety in real time by an independent DSMB. The independent DSMB will review safety listings until the 300th patient has completed 6 weeks of treatment i.e. 3 or 2 cycles completed. In the absence of a safety signal, the independent DSMB will follow all patients on a six-monthly basis until treatment of the last patient is terminated.

3.2 Number of Patients/Assignment to Treatment Groups

The planned total sample size for this study is 3450 patients, with 1150 patients in each of the three treatment arms. Approximately 350 study sites will be involved. Patients who drop-out after randomization (i.e. due to withdrawal of consent) will not be replaced. The total recruitment period is expected to be 23 months. Recruitment will stop when at least 3450 patients have been randomized. Recruitment of high risk stage II patients will be restricted to 570 patients.

A block design randomization procedure will be used. In order to avoid an imbalance of important prognostic factors in the patient population between the three treatment arms, patients will be stratified according to the following criteria:

- geographic region (for details please see section 6.9)
- AJCC/UICC stage (high-risk stage II vs. stage III N1 vs. stage III N2)

The patient randomization numbers will be generated by Roche and are to be allocated sequentially in the order in which the patients are enrolled. At the time of randomization, patients will be randomly assigned on a 1:1:1 basis to FOLFOX-4 or FOLFOX-4 + bevacizumab or XELOX + bevacizumab through a central interactive voice response system (IVRS).

The investigator or designee will use the Case Report Form (CRF) pre-printed with the appropriate patient number (CRF number). He/she will enter the randomization number and treatment group allocation assigned by the IVRS in the appropriate place on each patient's CRF.

4. STUDY POPULATION

4.1 Target Population

Patients who have undergone surgery for colon cancer, AJCC/UICC high-risk Stage II (as defined in 4. in paragraph 4.2) & stage III, with curative intent.

4.2 Inclusion Criteria

1. Signed written informed consent obtained prior to any study specific screening procedures.
2. Patient must be willing and able to comply with the protocol.
3. Age ≥ 18.
4. Histologically confirmed colon carcinoma, AJCC/UICC Stage II or Stage III (see Appendix 5) defined as a tumour location ≥15 cm from the anal verge by endoscopy or above the peritoneal reflection at surgery. The patient must not be a candidate for (neo) adjuvant radiotherapy.

Note! Stage II patients have to be considered as high-risk patients fulfilling one of the following criteria:

- T4 tumours,
- Patients presenting with bowel obstruction or perforation,
- Histological signs of vascular invasion (i.e. blood and lymphatic vessels) or perineural invasion,
- Patients aged less than 50 years,
- Patients with sub-optimal surgery (less than 12 nodes analyzed).

5. Curative surgery not less than 4 and not more than 8 weeks prior to randomization.
6. ECOG performance status 0 or 1.
7. Life expectancy of ≥ 5 years.

4.3 Exclusion Criteria

Patients who fulfil any of the following criteria will be excluded:

1. Macroscopic or microscopic evidence of remaining tumour. Patients should never have had any evidence of metastatic disease (including presence of tumour cells in the ascites). The isolated finding of cytokeratin positive cells in bone marrow is not considered evidence of metastatic disease for purposes of this study.
2. Carcinoembryonic antigen > 1.5 x ULN after surgery (during screening period).
3. For patients with colostomy, unwilling to delay revision until at least 28 days after treatment completion.
4. Major surgical procedure, open biopsy, or significant traumatic injury within 28 days prior to study treatment start, not fully healed wounds, or anticipation of the need for major surgical procedure during the course of the study. CVAD for chemotherapy administration must be inserted at least 2 days prior to treatment start. For details refer to Section 5.1.2.
5. Previous anti-angiogenic treatment for any malignancy; cytotoxic chemotherapy, radiotherapy or immunotherapy for colon cancer.
6. Other malignancies within the last 5 years (other than curatively treated basal cell carcinoma of the skin and/or in situ carcinoma of the cervix).
7. Females with a positive or no pregnancy test (within 7 days before treatment start) unless childbearing potential can be otherwise excluded (postmenopausal i.e. amenorrheic for at least 2 years, hysterectomy or oophorectomy).
8. Lactating women.
9. Fertile women (<2 years after last menstruation) and men of childbearing potential not willing to use effective means of contraception.
10. History or evidence upon physical examination of CNS disease (e.g., primary brain tumour, seizure not controlled with standard medical therapy, any brain metastases).
11. History of psychiatric disability judged by the investigator to be clinically significant, precluding informed consent or interfering with compliance for oral drug intake.
12. Clinically significant (i.e. active) cardiovascular disease e.g. cerebrovascular accidents (≤ 6 months prior to randomisation), myocardial infarction (≤ 1 year prior to randomisation), uncontrolled hypertension while receiving chronic medication, unstable angina, New York Heart Association (NYHA) Grade II or greater congestive heart failure, or serious cardiac arrhythmia requiring medication.
13. Lack of physical integrity of the upper gastro-intestinal tract, malabsorption syndrome, or inability to take oral medication.
14. Interstitial pneumonia or extensive symptomatic fibrosis of the lungs.
15. Known peripheral neuropathy ≥ CTCAE v 3.0 Grade 1. Absence of deep tendon reflexes (DTRs) as the sole neurological abnormality does not render the patient ineligible.

16. Organ allografts requiring immunosuppressive therapy.
17. Serious, non-healing wound, ulcer, or bone fracture.
18. Evidence of bleeding diathesis or coagulopathy.
19. Current or recent (within 10 days prior to study treatment start) use of full-dose oral or parenteral anticoagulants or thrombolytic agent for therapeutic purposes.
20. Chronic, daily treatment with high-dose aspirin (>325mg/day) or nonsteroidal anti-inflammatory medications (those known to inhibit platelet function at doses used to treat chronic inflammatory diseases). Patients can be rendered eligible by changing the treatment to COX II inhibitors.
21. Chronic treatment with corticosteroids (dose of ≥ 10 mg/day methylprednisolone equivalent) (excluding inhaled steroids).
22. Serious intercurrent infections (uncontrolled or requiring treatment).
23. Known dihydropyrimidine dehydrogenase (DPD) deficiency.
24. Current or recent (within the 28 days prior to randomization) treatment with another investigational drug or participation in another investigational study.
25. Patients with known allergy to Chinese hamster ovary cell proteins or other recombinant human or humanized antibodies or to any excipients of bevacizumab formulation, platinum compounds or to any other components of the study drugs.
26. History or presence of other disease, metabolic dysfunction, physical examination finding, or clinical laboratory finding giving reasonable suspicion of a disease or condition that contraindicates use of an investigational drug or patient at high risk from treatment complications.
27. Presence of proteinuria at baseline as defined by:
    - Patients with > 1g of protein/24 hr by a 24-hour urine collection.
28. Any laboratory values at baseline are as follows:

    Haematology:
    - Absolute neutrophil count (ANC) < 1.5 x 10^9/L
    - Platelet count < 100 x 10^9/L
    - Haemoglobin < 9 g/dL (may be transfused to maintain or exceed this level)
    - International Normalized Ratio (INR) > 1.5
    - APTT ≥ 1.5 x Upper Limit of Normal (ULN)

    Biochemistry:
    - Total bilirubin > 1.5 x ULN
    - AST, ALT > 2.5 x ULN
    - Alkaline phosphatase > 2.5 x ULN
    - Serum Creatinine > 1.5 x ULN or creatinine clearance ≤ 50 mL/min (calculated according to Cockroft and Gault, Appendix 6).

4.4 Concomitant Medication, Intervention and Treatment

At study initiation, patients should continue with their concomitant medications, as directed by their physician.

All concomitant medication must be recorded on the CRF. Additionally, any diagnostic, therapeutic or surgical procedure performed during the study period, should be recorded including the date, indication, description of the procedure(s) and any clinical findings.

Any endoscopic investigations have to be assessed carefully for the potential benefit to the patient and documented in the CRF.

4.4.1 Supportive Measures

Supportive care for treatment-related symptoms will be offered as needed to all patients in this study.

If nausea, vomiting or diarrhea occurs, effective symptomatic treatment must be initiated.

Antiemetic treatment and prophylaxis: For acute nausea and vomiting, a 5-HT3 antagonist with corticosteroids prior to infusion would be considered standard premedication for oxaliplatin. For delayed nausea and vomiting, an oral 5-HT3 antagonist is the first option; metoclopramide, alizapride and prochlorperazine may be also used. Alizapride, metoclopramide or prochlorperazine are recommended for primary and secondary prophylaxis of capecitabine-induced nausea/vomiting, and patients should have a supply available at home. Alizapride, metoclopramide or prochlorperazine are also recommended for primary and secondary prophylaxis of 5-FU/LV-induced (arms A and B) nausea/vomiting.

Diarrhea: In general, it is recommended that loperamide treatment is used as standard therapy. The use of drugs with laxative properties should be avoided because of the potential for exacerbation of diarrhea. Patients should be advised to contact their physician to discuss any laxative use.

Hematopoietic growth factors (i.e., G- or GM-CSF) may be used according to institutional or other specific guidelines (e.g. country, regional, or oncology organizations as ASCO, etc) to treat febrile neutropenia, but should not be used as primary prophylaxis. Use of any supplementary growth factor must be documented in the patient record. Growth factors must be discontinued at least 48 hours prior to initiation of the next cycle of chemotherapy.

Vitamin B6 (pyridoxine) should not be used as treatment/prophylaxis for hand-foot syndrome since impaired efficacy has been reported when used concomitantly with cisplatin [55].

Prevention of alopecia with *cold cap* or stomatitis with *iced mouth rinses* is not permitted due to the risk of triggering cold-related dysesthesias.

The use of prophylactic medication such as *Mg^{++}, Ca^{++} infusions* or others for prevention of oxaliplatin-induced neuropathy is at the discretion of the investigator, however, these

treatments are not recommended by the protocol as their benefits have not been clearly established.

4.4.2 Oral Coumarin-Derived Anticoagulants, Heparin, Aspirin and Non-Steroidal Anti-Inflammatory Drugs

The most severe toxicities seen with bevacizumab to date have been hemorrhage, thrombosis and gastro-intestinal perforation. For this reason, the use of the following agents is limited. However, administration of 5-FU was associated with hemorrhage and gastro-intestinal perforation as well (7.5.4).

Full-dose oral coumarin-derived anticoagulants (INR>1.5) or heparin, thrombolytic agents, or chronic, daily treatment with aspirin (>325 mg/day) or non-steroidal anti-inflammatory medications (those known to inhibit platelet function at doses used to treat chronic inflammatory diseases) are excluded *at entry into the study.*

The use of low-dose oral coumarin-derived anticoagulants, heparin or low-molecular heparins is permitted, as is low-dose aspirin (≤ 325 mg/day), occasional use of non-steroidal anti-inflammatory medication, or regular use of non-steroidal anti-inflammatory medication of the kind known not to inhibit platelet function. Note: Patients receiving concomitant 5-FU or capecitabine and oral coumarin-derivative anticoagulant therapy should have their anticoagulant response (INR or prothrombin time) monitored closely and the anticoagulant dose adjusted accordingly (for details please refer to 7.5.2, 7.5.4).

In patients who experience thromboembolic events during study treatment full dose anticoagulant are allowed and information on anticoagulant treatment (including doses) will be collected and recorded in the CRF.

INR will be assessed at baseline for all patients. In patients treated with oral coumarin-derived anticoagulants INR will be checked at least before start of every chemotherapy cycle.

In patients treated with full dose oral anticoagulants due to thromboembolic event during study treatment, INR must be checked at least every second day the first week of treatment, at least 2 times/week the following treatment weeks until a stable therapeutic level of INR has been achieved and at least once every 3rd week when the weekly dose has been established and INR is stable with this dose (see section 7.3.2.3, Table 12).

4.4.3 Phenytoin

Increased phenytoin plasma concentrations have been reported during concomitant use of capecitabine with phenytoin. Formal drug-drug interaction studies with phenytoin have not been conducted. Patients taking phenytoin concomitantly with capecitabine should be regularly monitored for increased phenytoin plasma concentrations and associated clinical symptoms [78].

4.4.4 Antivirals and Antiprotozoals

Capecitabine or 5-FU should not be administered together with the antiviral drug sorivudine or its chemically related analogues, such as brivudine. A clinically significant drug-drug interaction between sorivudine and 5-FU, resulting from the inhibition of DPD by sorivudine, has been described in the literature [79]. This interaction, which leads to increased fluoropyrimidine toxicity, is potentially fatal.

Metronidazole increased the toxicity of fluorouracil in patients with colorectal cancer, apparently by reducing the clearance of the antineoplastic [80]. As it has been described in the literature, caution should be exercised.

4.4.5 Gastro-intestinal Drugs

Pretreatment with cimetidine for 4 weeks led to increased plasma concentrations of fluorouracil following intravenous and oral administration in six patients. The effect was probably due to a combination of hepatic enzyme inhibition and reduced hepatic blood flow. No such effect was seen following single doses of cimetidine in five patients or pretreatment for just one week in six. Care is required in patients taking both drugs simultaneously [80].

4.4.6 Other Anticancer Therapies

The use of other cytotoxic agents, investigational drugs, active or passive immunotherapy for colon cancer is not allowed during the Study Treatment Phase nor while the patient is disease-free in the follow-up phase. Patients withdrawn from the study treatment due to toxicity who receive any other cancer therapies will enter the follow up phase and be followed for recurrence and survival.

Patients that have radiotherapy during the Study Treatment Phase or the Follow-up phase will be considered to have had disease recurrence.

5. SCHEDULE OF ASSESSMENTS AND PROCEDURES

Treatment details and assessments can be found in sections 5.2 and 6.1.

Study assessments and procedures will be performed as shown in Table 6 - Table 11 as follows:

Table 6: Arm A, Part 1 of Study Treatment Phase (Week 1-24)

Table 7: Arm B, Part 1 of Study Treatment Phase (Week 1-24)

Table 8: Arm C, Part 1 of Study Treatment Phase (Week 1-24)

Table 9: Arm A, Part 2 of Study Treatment Phase (Week 25-48), Observation Only

Table 10: Arm B & Arm C, Part 2 of Study Treatment Phase (Week 25-48), Bevacizumab Monotherapy Phase

Table 11: All (Follow up Phase)

Table 6 Schedule of Assessments: Week 1 - 24 Arm A: FOLFOX-4

	Pre-randomization (c)		Study Treatment Phase (p) (all visits within ± 3 days of schedule)											
**Cycle**	**Screen**	**BS**	**1**	**2**	**3**	**4**	**5**	**6**	**7**	**8**	**9**	**10**	**11**	**12**
**Study Week**	**- 2 to -1**	**- 1**	**1**	**3**	**5**	**7**	**9**	**11**	**13**	**15**	**17**	**19**	**21**	**23**
**Study Day**	**- 14 to -1**	**- 7 to -1**	**1**	**15**	**29**	**43**	**57**	**71**	**85**	**99**	**113**	**127**	**141**	**155**
Informed consent [a]	X													
Demographic data	X [b]													
Medical history	X [b]													
Colon cancer treatment history	X [b]													
Concomitant treatment	X [b]		X	X	X	X	X	X	X	X	X	X	X	X
Physical examination [f]	X [b]		X	X	X	X	X	X	X	X	X	X	X	X
Vital signs/ECOG performance status		X [b]	X	X	X	X	X	X	X	X	X	X	X	X
Chest X–ray [h]	X [b, l]		As clinically indicated											
ECG	X [b]		As clinically indicated											
CEA	X [b]		As clinically indicated											
Tumour assessment	X [b,d]		As clinically indicated [e]											
Hematology [m]		X [b]	X [g, n]	X [n]	X [n]	X [n]	X [n]	X [n]	X [n]	X [n]	X [n]	X [n]	X [n]	X [n]
Coagulation parameter [m]		X [b]	X [k,n]	X [k,n]	X [k,n]	X [k,n]	X [k,n]	X [k,n]	X [k,n]	X [k,n]	X [k,n]	X [k,n]	X [k,n]	X [k,n]
Specific coagulation parameter: D-dimer.		X												
Blood chemistry [m]		X [b]	X [g]	X	X	X	X	X	X	X	X	X	X	X
Urinalysis [m]		X [b,q]	X [g, i, n]	X [i, n]	X [i, n]	X [i, n]	X [i, n]	X [i, n]	X [i, n]	X [i, n]	X [i, n]	X [i, n]	X [i, n]	X [i, n]
Pregnancy test (if applicable)		X [b]	X [g]	As clinically indicated										
AEs [j]			X	X	X	X	X	X	X	X	X	X	X	X
Medical care utilization [j]			X	X	X	X	X	X	X	X	X	X	X	X
Chemotherapy Convenience and Satisfaction Questionnaire (CCSQ)		X [r]				X			X			X		
Study Treatment Administration [o]			X	X	X	X	X	X	X	X	X	X	X	X

**Table 6 Week 1 – 24, Arm A: FOLFOX-4 (footnotes)**
[(a)] Informed consent must be obtained before any study specific screening procedures are performed.<br>[(b)] Results/data available and reviewed by investigator before randomization.<br>[(c)] Up to 14 days before randomization. Treatment to start up to 7 days after randomization.<br>[(d)] Assessments to be performed include *abdominal & pelvic* CT/MRI AND *chest* CT/MRI or X-ray. Assessments are required to be performed up to 3 weeks before surgery, or after surgery up to randomization i.e. within 11 week before randomization.<br>[(e)] Assessments to be performed include *abdominal & pelvic* CT/MRI or US AND *chest* CT/MRI or X-ray. All assessments, i.e. also non-protocol specified, used to confirm a recurrence/new tumour should be recorded in the CRF. The use of oral and iv contrast etc should, as long as it is clinically possible, be kept constant. Tumour assessments should, as long as possible, be made by the same investigator/radiologist for all assessments for each patient during the study.<br>[(f)] Including a general neurological examination.<br>[(g)] If >7 days after baseline assessments, repeat tests. For repeat pregnancy test, urine is acceptable<br>[(h)] Chest X-ray is to be performed as a safety assessment.<br>[(i)] If ≥ 2+ proteinuria (dipstick): collect 24-hour urine for determination of total protein.The results of the 24-hour urine collection should not delay the administration of treatment<br>[(j)] Record throughout therapy and up to 28 days after end of study treatment phase.<br>[(k)] INR only, to be performed only for patients receiving oral coumarin-derivative anticoagulant therapy.<br>[(l)] To be done within 3 weeks before randomization. If chest CT/MRI or X-ray are performed within 3 weeks before randomization (e.g. as tumour assessment), no additional chest X-ray is necessary.<br>[(m)] For all laboratory assessments (haematology, biochemistry, coagulation and urinalysis) except screening/baseline, a window of - 2 days before treatment is acceptable.<br>[(n)] The investigator must review haematology, INR and urinalysis results before starting each cycle of treatment.<br>[(o)] In general, for non-medical reasons, deviations of ± 3 days from schedule are permitted.<br>[(p)] Patients who withdraw prematurely any time during study treatment should return for a safety follow up visit within 28 days post last dose. Assessments to be performed are according to Table 11, End of Treatment Safety Assessment. Additionally, patients withdrawing prior to week 25 should have tumour assessments (including CEA) performed at the scheduled week 25 time point (Table 9).<br>[(q)] Baseline proteinuria assessment 24-hour urine collection and dipstick.<br>[(r)] Baseline CCSQ question to be answered before randomisation.

Table 7 Schedule of Assessments: Week 1 - 24 Arm B: FOLFOX-4 + bevacizumab

	Pre-randomization (c)		Study Treatment Phase (p) (all visits within ± 3 days of schedule)											
**Cycle**	**Screen**	**BS**	**1**	**2**	**3**	**4**	**5**	**6**	**7**	**8**	**9**	**10**	**11**	**12**
**Study Week**	**- 2 to -1**	**- 1**	**1**	**3**	**5**	**7**	**9**	**11**	**13**	**15**	**17**	**19**	**21**	**23**
**Study Day**	**- 14 to -1**	**- 7 to -1**	**1**	**15**	**29**	**43**	**57**	**71**	**85**	**99**	**113**	**127**	**141**	**155**
Informed consent [a]	X													
Demographic data	X [b]													
Medical history	X [b]													
Colon cancer treatment history	X [b]													
Concomitant treatment	X [b]		X	X	X	X	X	X	X	X	X	X	X	X
Physical examination [f]	X [b]		X	X	X	X	X	X	X	X	X	X	X	X
Vital signs/ECOG performance status		X [b]	X	X	X	X	X	X	X	X	X	X	X	X
Chest X–ray [h]	X [b, l]		As clinically indicated											
ECG	X [b]		As clinically indicated											
CEA	X [b]		As clinically indicated											
Tumour assessment	X [b,d]		As clinically indicated [e]											
Hematology [m]		X [b]	X (g, n)	X [n]	X [n]	X [n]	X [n]	X [n]	X [n]	X [n]	X [n]	X [n]	X [n]	X [n]
Coagulation parameter [m]		X [b]	X (k,n)	X (k,n)	X (k,n)	X (k,n)	X (k,n)	X (k,n)	X (k,n)	X (k,n)	X (k,n)	X (k,n)	X (k,n)	X (k,n)
Specific coagulation parameter: D-dimer		X												
Blood chemistry [m]		X [b]	X[g]	X	X	X	X	X	X	X	X	X	X	X
Urinalysis [m]		X [b,r]	X (g, i,n)	X (i,n)	X (i,n)	X (i,n)	X (i,n)	X (i,n)	X (i,n)	X (i,n)	X (i,n)	X (i,n)	X (i,n)	X (i,n)
Pregnancy test (if applicable)		X [b]	X[g]	As clinically indicated										
Anti-bevacizumab antibodies (HAHA) [q]			X											
AEs [j]			X	X	X	X	X	X	X	X	X	X	X	X
Medical care utilization [j]			X	X	X	X	X	X	X	X	X	X	X	X
Chemotherapy Convenience and Satisfaction Questionnaire (CCSQ)		X[s]				X			X			X		
Study Treatment Administration [o]			X	X	X	X	X	X	X	X	X	X	X	X

Table 7 Week 1 – 24, Arm B: FOLFOX-4 + bevacizumab (footnotes)

(a) Informed consent must be obtained before any study specific screening procedures are performed.
(b) Results/data available and reviewed by investigator before randomization.
(c) Up to 14 days before randomization. Treatment to start up to 7 days after randomization.
(d) Assessments to be performed include *abdominal & pelvic* CT/MRI AND *chest* CT/MRI or X-ray. Assessments are required to be performed up to 3 weeks before surgery, or after surgery up to randomization i.e. within 11 week before randomization.
(e) Assessments to be performed include *abdominal & pelvic* CT/MRI or US AND *chest* CT/MRI or X-ray. All assessments, i.e. also non-protocol specified, used to confirm a recurrence/new tumour should be recorded in the CRF. The use of oral and iv contrast etc should, as long as it is clinically possible, be kept constant. Tumour assessments should, as long as possible, be made by the same investigator/radiologist for all assessments for each patient during the study.
(f) Including a general neurological examination.
(g) If >7 days after baseline assessments, repeat tests. For repeat pregnancy test, urine is acceptable
(h) Chest X-ray is to be performed as a safety assessment.
(i) If ≥ 2+ proteinuria (dipstick): collect 24-hour urine for determination of total protein. The results of the 24-hour urine collection should not delay the administration of treatment. For detailed guidance see 7.3.2.5 and Appendix 11.
(j) Record throughout therapy and up to 28 days after end of study treatment phase.
(k) INR only, to be performed only for patients receiving oral coumarin-derivative anticoagulant therapy.
(l) To be done within 3 weeks before randomization. If chest CT/MRI or X-ray are performed within 3 weeks before randomization (e.g. as tumour assessment), no additional chest X-ray is necessary.
(m) For all laboratory assessments (haematology, biochemistry, coagulation and urinalysis) except screening/baseline, a window of - 2 days before treatment is acceptable.
(n) The investigator must review hematology, INR and urinalysis results before starting each cycle of treatment.
(o) In general, for non-medical reasons, deviations of ± 3 days from schedule are permitted.
(p) Patients who withdraw prematurely any time during study treatment should return for a safety follow up visit within 28 days post last dose. Assessments to be performed are according to Table 11, End of Treatment Safety Assessment. Additionally, patients withdrawing prior to week 25 should have tumour assessments (including CEA) performed at the scheduled week 25 time point (Table 10).
(q) Mandatory.
(r) Baseline proteinuria assessment by 24-hour urine collection and dipstick.
(s) Baseline CCSQ question to be answered before randomisation.

Table 8 Schedule of Assessments: Week 1 - 24
Arm C, XELOX + bevacizumab

	Pre-randomization (c)		Study Treatment Phase (p) (all visits within ± 3 days of schedule)							
**Cycle**	**Screen**	**Baseline**	**1**	**2**	**3**	**4**	**5**	**6**	**7**	**8**
**Study Week**	**- 2 to -1**	**- 1**	**1**	**4**	**7**	**10**	**13**	**16**	**19**	**22**
**Study Day**	**- 14 to -1**	**- 7 to -1**	**1**	**22**	**43**	**64**	**85**	**106**	**127**	**148**
Informed consent (a)	X									
Demographic data	X (b)									
Medical history	X (b)									
Colon cancer treatment history	X (b)									
Concomitant treatment	X (b)		X	X	X	X	X	X	X	X
Physical examination (f)	X (b)		X	X	X	X	X	X	X	X
Vital signs/ECOG performance status		X (b)	X	X	X	X	X	X	X	X
Chest X–ray (h)	X (b, l)		As clinically indicated							
ECG	X (b)		As clinically indicated							
CEA	X (b)		As clinically indicated							
Tumour assessment	X (b, d)		As clinically indicated (e)							
Hematology (m)		X (b,)	X(g,n)	X (n)	X (n)	X (n)	X (n)	X (n)	X (n)	X (n)
Coagulation parameter (m)		X (b)	X (k,n)	X (k,n)	X (k,n)	X (k,n)	X (k,n)	X (k,n)	X (k,n)	X (k,n)
Specific coagulation parameter: D-dimer		X								
Blood chemistry (m)		X (b),	X(g)	X	X	X	X	X	X	X
Urinalysis (m)		X (b,r)	X(g, i,n)	X (i,n)	X (i,n)	X (i,n)	X (i,n)	X (i,n)	X (i,n)	X (i,n)
Anti-bevacizumab antibodies (HAHA) (q)			X							
Pregnancy test (if applicable)		X (b)	X (g)	As clinically indicated						
AEs (j)			X	X	X	X	X	X	X	X
Medical care utilization (j)			X	X	X	X	X	X	X	X
Chemotherapy Convenience and Satisfaction Questionnaire (CCSQ)		X(s)			X		X		X	
Study Treatment Administration (o)			X	X	X	X	X	X	X	X

Table 8 Week 1 – 24, Arm C: XELOX + bevacizumab (footnotes)

(a) Informed consent must be obtained before any study specific screening procedures are performed.
(b) Results/data available and reviewed by investigator before randomization.
(c) Up to 14 days before randomization. Treatment to start up to 7 days after randomization.
(d) Assessments to be performed include *abdominal & pelvic* CT/MRI AND *chest* CT/MRI or X-ray. Assessments are required to be performed up to 3 weeks before surgery, or after surgery up to randomization i.e. within 11 week before randomization.
(e) Assessments to be performed include *abdominal & pelvic* CT/MRI or US AND *chest* CT/MRI or X-ray. All assessments, i.e. also non-protocol specified, used to confirm a recurrence/new tumour should be recorded in the CRF. The use of oral and iv contrast etc should, as long as it is clinically possible, be kept constant. Tumour assessments should, as long as possible, be made by the same investigator/radiologist for all assessments for each patient during the study.
(f) Including a general neurological examination.
(g) If >7 days after baseline assessments, repeat tests. For repeat pregnancy test, urine is acceptable.
(h) Chest X-ray is to be performed as a safety assessment.
(i) If ≥ 2+ proteinuria (dipstick): collect 24-hour urine for determination of total protein. The results of the 24-hour urine collection should not delay the administration of treatment. For detailed guidance see 7.3.2.5 and Appendix 11.
(j) Record throughout therapy and up to 28 days after end of study treatment phase.
(k) INR only, to be performed only for patients receiving oral coumarin-derivative anticoagulant therapy.
(l) To be done within 3 weeks before randomization. If chest CT/MRI or X-ray are performed within 3 weeks before randomization (e.g. as tumour assessment), no additional chest X-ray is necessary.
(m) For all laboratory assessments (haematology, biochemistry, coagulation and urinalysis) except screening/baseline, a window of - 2 days before treatment is acceptable.
(n) The investigator must review hematology, INR and urinalysis results before starting each cycle of treatment.
(o) In general, for non-medical reasons, deviations of ± 3 days from schedule are permitted.
(p) Patients who withdraw prematurely any time during study treatment should return for a safety follow up visit within 28 days post last dose. Assessments to be performed are according to Table 11, End of Treatment Safety Assessment. Additionally, patients withdrawing prior to week 25 should have tumour assessments (including CEA) performed at the scheduled week 25 time point (Table 10).
(q) Mandatory.
(r) Baseline proteinuria assessment by 24-hour urine collection and dipstick.
(s) Baseline CCSQ question to be answered before randomisation.

Table 9 Schedule of Assessments: Week 25 - 48 Arm A: Observation Only

	Observation Only (all visits within ± 3 days of schedule)							
**Additional Cycle**	**1**	**2**	**3**	**4**	**5**	**6**	**7**	**8**
**Study Week**	**25**	**28 [h]**	**31**	**34**	**37**	**40**	**43**	**46**
**Study Day (n)**	**169**	**190**	**211**	**232**	**253**	**274**	**295**	**316**
Concomitant treatments	X	X	X	X	X	X	X	X
Physical examination [b]	X	X	X	X	X	X	X	X
Vital signs / ECOG performance status	X	X	X	X	X	X	X	X
Chest X–ray [c]	As clinically indicated							
ECG	(Aci)	X	As clinically indicated (Aci)					
CEA	X	As clinically indicated						
Tumour assessments [a]	X	As clinically indicated						
Hematology	X	X	As clinically indicated					
Coagulation parameter [e]	X	X	X	X	X	X	X	X
Blood chemistry	X	X	X	X	X	X	X	X
Urinalysis [d]	X	X	X	X	X	X	X	X
Pregnancy test (if applicable)	As clinically indicated							
AEs [f,g]	X	X	X	X	X	X	X	X
Medical care utilization [f]	X	X	X	X	X	X	X	X
Chemotherapy Convenience and Satisfaction Questionnaire	X							
Study medication	Not applicable							

[a] Assessments to be performed include *abdominal & pelvic* CT/MRI or US AND *chest* CT/MRI or X-ray. All assessments, i.e. also non-protocol specified, used to confirm a recurrence/new tumour should be recorded in the CRF. The use of oral and iv contrast etc should, as long as it is clinically possible, be kept constant. Tumour assessments should, as long as possible, be made by the same investigator/radiologist for all assessments for each patient during the study.
[b] Including a general neurological examination.
[c] Chest X-ray safety assessment is to be performed as clinically indicated.
[d] ≥ 2+ proteinuria (dipstick): collect 24-hour urine for determination of total protein.
[e] INR only, to be performed only for patients receiving oral coumarin-derivative anticoagulant therapy.
[f] Record throughout observation phase and up to 28 days after end of observation phase.
[g] For follow up and recording of targeted AEs refer protocol section 7.2.3.2.
[h] End of Treatment Safety Assessment for Arm A to be done at week 28.

Table 10 Schedule of Assessments: Week 25 - 48 Arm B & Arm C: Bevacizumab Monotherapy

	Bevacizumab Monotherapy Phase (j) (all visits within ± 3 days of schedule)							
**Additional Cycle**	**1**	**2**	**3**	**4**	**5**	**6**	**7**	**8**
**Study Week**	**25**	**28**	**31**	**34**	**37**	**40**	**43**	**46**
**Study Day**	**169**	**190**	**211**	**232**	**253**	**274**	**295**	**316**
Concomitant treatments	X	X	X	X	X	X	X	X
Physical examination (b)	X	X	X	X	X	X	X	X
Vital signs / ECOG performance status	X	X	X	X	X	X	X	X
Chest X–ray (c)	As clinically indicated							
ECG	As clinically indicated							
CEA	X	As clinically indicated						
Tumour assessment (a)	X	As clinically indicated						
Hematology (f)	X	As clinically indicated						
Coagulation parameter (e, f)	X (g)	X (g)	X (g)	X (g)	X (g)	X (g)	X (g)	X (g)
Blood chemistry (f)	X	X	X	X	X	X	X	X
Urinalysis (d, f)	X (g)	X (g)	X (g)	X (g)	X (g)	X (g)	X (g)	X (g)
Pregnancy test (if applicable)	As clinically indicated							
AEs (i,k)	X	X	X	X	X	X	X	X
Medical care utilization (i)	X	X	X	X	X	X	X	X
CCSQ	X							
Anti-bevacizumab anti-bodies (HAHA)	In the case of a withdrawal of the patient from treatment samples have to be drawn 3 and 6 months after last administration. Mandatory							
Study medication (h)	X	X	X	X	X	X	X	X

(a) Assessments to be performed include *abdominal & pelvic* CT/MRI or US AND *chest* CT/MRI or X-ray. All assessments, i.e. also non-protocol specified, used to confirm a recurrence/new tumour should be recorded in the CRF. The use of oral and iv contrast etc should, as long as it is clinically possible, be kept constant. Tumour assessments should, as long as possible, be made by the same investigator/radiologist for all assessments for each patient during the study.
(b) Including a general neurological examination.
(c) Chest X-ray safety assessment is to be performed as clinically indicated.
(d) ≥ 2+ proteinuria (dipstick): collect 24-hour urine for determination of total protein at the first incidence. The results of the 24-hour urine collection should not delay the administration of treatment. For detailed guidance see 7.3.2.5 and Appendix 11 .
(e) INR only, to be performed only for patients receiving oral coumarin-derivative anticoagulant therapy.
(f) For all laboratory assessments (haematology, biochemistry, coagulation and urinalysis), a window of - 2 days before treatment is acceptable.
(g) The investigator must review INR and urinalysis results before starting each cycle of treatment.
(h) In general, for non-medical reasons, deviations of ± 3 days from schedule are permitted.
(i) Record throughout therapy and up to 28 days after end of study treatment phase.
(j) Patients who withdraw prematurely any time during bevacizumab treatment should return for a safety follow up visit within 28 days post last dose. Assessments to be performed are according to Table 11, End of Treatment Safety Assessment.
(k) For follow up and recording of targeted AEs refer protocol section 7.2.3.2

Table 11 Schedule of Assessments: Follow - Up Phase All Treatment Arms

	Time After Randomization							
	**1 yr (d)**	**1.5 yrs (e)**	**2 yrs (e)**	**2.5 yrs (e)**	**3 yrs (e)**	**3.5 yrs (e)**	**4 yrs (e)**	**> 4 yrs (e, f)**
Anti-bevacizu-mab anti-bodies (HAHA) (a)	X	X						
CEA (c)	X	X	X	X	X	X	X	every 6 months
Targeted AEs	please refer to section 7.2.3.2							
Tumour assessments (b)	X	X	X	X	X	X	X	yearly
Survival	X	X	X	X	X	X	X	yearly
Additional cancer therapy	To be recorded as it occurs							
**End of Treatment Safety Assessment (g)**								
Physical examination (h)	X							
Vital signs / ECOG performance status	X							
AEs	X							
Blood chemistry	X							
Hematology	X							
Urinalysis(i)	X							
ECG	X							
Concomitant treatments	X							

(a) Arms B and C only.
(b) Assessments to be performed include *abdominal & pelvic* CT/MRI or US AND *chest* CT/MRI or X-ray. All assessments, i.e. also non-protocol specified, used to confirm a recurrence/new tumour should be recorded in the CRF (performed until a recurrence/new CRC). The use of oral and iv contrast etc should, as long as it is clinically possible, be kept constant. Tumour assessments should, as long as possible, be made by the same investigator/radiologist for all assessments for each patient during the study.
(c) All test i.e. also non-protocol specified, used to confirm recurrence/new tumour should be recorded in the CRF (performed until a recurrence/new CRC).
(d) Investigations can take place on the scheduled day ± 7 days.
(e) Investigations can take place on the scheduled day ± 14 days.
(f) Patients will be followed for recurrence and survival until the end of the study.
(g) Applicable to Arms B and C only. For Arm A the End of Treatment Safety Assessment is to be done at Week 28 (Table 9). For patients withdrawing prematurely see Section 5.2.4.1.
(h) Including a general neurological examination.
(i) ≥ 2+ proteinuria (dipstick): collect 24-hour urine for determination of total protein For detailed guidance see 7.3.2.5 and Appendix 11.

5.1 Screening Examination and Eligibility Screening Form

Written informed consent must be obtained prior to the patient undergoing any study-specific procedures.

An Eligibility Screening Form (ESF) documenting the patient's fulfilment of the entry criteria for all patients considered for the study is to be completed by the investigator/designee. Patients who are considered for study entry, but who fail to meet the eligibility requirements, should also have an ESF completed with the reason for lack of eligibility given, since this provides information on the selected trial population. These patients will not be entered on the clinical trials database. The ESFs for patients who fail to meet the eligibility requirements should be kept in the study files at the sites.

5.1.1 Screening Procedures for Study Entry

The screening procedure may be done in different stages. The first group of assessments can be done at any time within 2 weeks prior to randomization (day -14 to day -1) except for imaging studies (CT, MRI, X-ray), which may be obtained up to 3 weeks before surgery or after surgery. (Note: surgery should occur within 8 weeks before randomization, allowing a maximum of 11 weeks between scans and randomization). The last group of assessments must be done within 7 days prior to randomization (day -7 to day -1). Treatment should be started not more than 7 days after randomization. If pregnancy test, haematology, urinalysis and/or blood chemistry are done > 7 days before treatment start the tests need to be repeated before initiation of therapy.

1. Tumour assessments: *abdominal & pelvic* CT / MRI AND *chest* CT / MRI or X-ray may be obtained up to 3 weeks before surgery (i.e. up to 11 weeks prior to randomization), but may be done after surgery (up to randomization) as well.
2. Chest X-ray safety assessment must be done within 3 weeks before randomization. If chest CT/MRI or X-ray are performed within 3 weeks before randomization (e.g. as tumour assessment), no additional chest X-ray is necessary.
3. Assessments to be made up to 14 days before randomization include: demographic data, medical history, cancer/treatment history, concomitant disease/treatment, physical examination (including basic neurological exam by investigator), electrocardiogram (ECG) and CEA determination.
4. Assessments to be made within 7 days before randomization: height, weight, vital signs (body temperature, blood pressure, and pulse/heart rate), ECOG performance status, hematology, blood chemistry (including creatinine clearance calculation), INR, APTT and plasma D-dimer, serum pregnancy test (for all women less than 2 years amenorrheic), dipstick urinalysis for proteinuria, 24-hour urine collection for determination of total protein (see Appendix 11), and CCSQ baseline question.
5. Urine pregnancy test is required prior to first administration of study treatment if more than 7 days have elapsed from baseline serum pregnancy test.
6. Arms B and C: Serum will be collected prior to first administration of bevacizumab for central analysis of antibodies to rHuMAb VEGF.

5.1.2 Central Venous Access Device (CVAD)

Patients receiving FOLFOX-4 will require a CVAD in general. Since oxaliplatin will be given parenterally on all three arms of the trial, it is possible that patients on the XELOX arm will have a CVAD as well. However, the placement of a CVAD is not mandatory for patients assigned to arms A or B. Nevertheless, appropriate safety measures have to be in place to avoid extravasation of drugs (if a peripheral line is used, patients must be hospitalized). As a precautionary measurement, it is required to maintain an interval of 7 days between the insertion of a CVAD and the onset of bevacizumab treatment.

CVAD placement and complications will be monitored both as an assessment of treatment-related complications, and as part of the medical care utilization assessments. Date of placement of CVAD will be noted in the medical record and recorded in the CRF. Episodes of CVAD removal or replacement will be recorded. Episodes of CVAD-related thrombosis, infection, or dysfunction will be recorded.

For centers where it is not feasible to keep the recommended 7-day interval between placement of a central line and first bevacizumab administration, it is permitted to start the treatment earlier i.e. according to the common local practice, but no less than 2 days after placement of a central line. Besides local practice, the status of the wound has to be checked before onset of treatment.

5.2 Study Assessments

Study assessments are performed during the treatment phase and to a lesser extent in the follow-up phase. The treatment phase consists of two parts of 24 weeks for a total of 48 weeks (Figure 2). The first part consists of treatment with either FOLFOX-4, FOLFOX-4 in combination with bevacizumab, or XELOX in combination with bevacizumab. The 2nd part consists of an observation period for patients assigned to arm A or a treatment period with bevacizumab as a single agent in arms B and C.

During the treatment phase, for non-medical reasons deviations of ± 3 days from visit schedule are permitted (Table 6, Table 7, Table 8, Table 9, Table 10).

For details of the follow-up phase please refer to 5.2.4.

5.2.1 Clinical Assessments

5.2.1.1 Assessments of Efficacy and Recurrence

The primary efficacy parameter will be disease-free survival (DFS), based on assessment of tumour recurrence or appearance of a new colorectal cancer. All assessments, i.e. also non-protocol specified, used to confirm a recurrence/new tumour should be recorded in the Case Report Form. DFS is defined as the time from randomization to the time of occurrence of one of the events listed below. Tumour assessments (*abdominal & pelvic* CT / MRI or US AND *chest* X-ray or CT / MRI) and a CEA determination should be performed 6 and 12 months after randomization or if the patient shows signs of a recurrence/new colorectal cancer (e.g. clinical status). Note: suspicious lesions detected by US or chest X-ray must be confirmed by CT / MRI examination. Possible re-operation or/and further cancer therapy will be recorded.

For the purposes of the study, events that will be utilized to determine that a patient is no longer disease-free are defined as:

- Evidence that the patient has a recurrence of the original cancer,
- Evidence that the patient has developed a new colorectal cancer, and
- Death due to any cause.

Note: Any recurrence of the original cancer or appearance of a new colorectal cancer should be proven by cytology or histology when possible. An isolated event of increased CEA, or unexplained clinical deterioration are not considered to be evidence of recurrence without support of other objective measurements (e.g., radiology, histology/cytology). Date of recurrence is defined as the date of definitive assessment by objective measurements. Patients will be followed for survival as outlined in Table 11.

In case of a confirmed recurrence or the development of a new colorectal cancer during the Study Treatment Phase, the patient will be taken off study treatment and will be followed-up for survival (Section 5.2.3). In the case of recurrence or development of a new colorectal cancer, further treatment is at the discretion of the investigator.

Consistency of consecutive tumour assessments should be ensured for all assessments for all patients, with the same technique being used throughout the study. The use of oral and IV contrast is recommended and should, as long as it is clinically possible, be kept consistent. Evaluations should be made by the same investigator/radiologist for each patient during the study, to the extent that this is feasible.

5.2.1.2 Standard Clinical Assessments (Safety Assessments excluding laboratory exams)

Timing for assessments is given in Table 6 - Table 11.

- Physical examination (including basic neurological exam by investigator),
- ECOG performance status (see Appendix 13),
- Vital signs [weight, body temperature, blood pressure, pulse/ heart rate],
- On each (scheduled) visit, patients will be assessed for adverse events (grading by the common terminology criteria for adverse events - CTCAE version 3.0) [89],
- MCU,
- CCSQ (see Section 5.2.9),
- Concomitant treatments,
- Tumour assessments (6 months after randomization and as clinically required), see also Section 5.2.1.1.

Electrocardiogram (obligatory at week 28 in arm A) and chest X-rays as clinically required.

5.2.2 Laboratory Assessments

The local laboratory will perform the analyses.

Normal ranges for the study laboratory parameters must be provided to the Sponsor.

NOTE: If the site utilizes a laboratory which for CEA uses normal ranges with different values for smokers and non-smokers, normal ranges for smokers will be applied and must be provided to the sponsor.

All laboratory tests during treatment must be performed according to the Schedule of Assessments [Table 6, Table 7, Table 8, Table 9, Table 10] prior to the start of the next treatment cycle, and the results entered into the CRF. It is permitted to perform the blood tests up to 2 days before the scheduled treatment and reviewed by investigator before starting treatment.

The following laboratory tests are to be done:

- White blood cell count with differentials (lymphocytes, neutrophils), red blood cell count, hemoglobin, hematocrit and platelet count
- Bilirubin (total and direct), ASAT, ALAT, alkaline phosphatase, albumin, LDH
- Serum creatinine
- Glucose
- Electrolytes (sodium, potassium)
- Calcium
- INR (for patients receiving oral anticoagulant treatment)
- CEA (as clinically indicated)
- Urinalysis: Proteinuria by dipstick has to be assessed according to the SoA unless proteinuria has been determined by 24-hour urine collection.
- Serum pregnancy tests (if clinically indicated)

5.2.3 End of Treatment Safety Assessment

A safety assessment is mandatory for patients of all treatment groups after the end of treatment i.e. after week 23 for patients in arm A and after week 46 for patients in arms B and C. In order to avoid additional visits, for Arm A this should be performed during the second visit of the observation period (week 28) and for Arms B and C at the first visit of the follow-up phase (week 52 = 1 yr). For patients withdrawing prematurely the end of treatment safety visit is to be performed 28 days post last dose (Please refer to section 5.2.4.1).

The following assessments are to be done:

– Physical examination (including a general neurological examination),
– Vital signs / ECOG performance status,
– AEs / Concomitant treatments,
– Safety lab (Hematology, Blood chemistry, Urinalysis),
– ECG.

5.2.4 Follow-up Phase

The Follow-up Phase will begin when the patient completes or terminates the Study Treatment Phase (Note: the observation period in Arm A is part of study treatment phase). Investigations for the one year follow up visit should take place on the scheduled day ± 7 days. Subsequent visits should take place on the scheduled day ± 14 days.

During the Follow-up Phase, the following parameters will be recorded (see Table 11 in Section 5.2):

– During follow-up CEA determinations will be done every 6 months (performed until a confirmed recurrence/new CRC).
– HAHAs at 1 and 1.5 years (arms B and C only).
– Targeted adverse events (please refer to section 7.2.3.2).
– Tumour assessments with abdominal& pelvic CT / MRI or US AND chest X-ray or CT / MRI will be done after the end of the study treatment phase, then every 6 months for the first 4 years after randomization and then annually (performed until a confirmed recurrence/new CRC). Note: suspicious lesions detected by US or chest X-ray must be confirmed by CT / MRI.
– Additional tumour assessments including endoscopy may be required if the patient shows signs of a recurrence (e.g. clinical status or increase of CEA levels) or a new colorectal cancer. All assessments, i.e. also non-protocol specified, used to confirm a recurrence/new tumour should be recorded in the Case Report Form.
– Additional cancer therapy to be recorded as it occurs.
– Survival status should be assessed every 6 months in the first 4 years after randomization and then annually thereafter (may be by telephone contact rather than visit).

Patients will be followed for recurrence/appearance of new colorectal cancer and survival until the time of the primary analysis. Thereafter, patients will be followed for recurrence/appearance of new colorectal cancer and survival for further 2 years (end of the study follow up). In case of a confirmed recurrence/appearance of new colorectal cancer patients will be followed for survival until the end of study follow up period as well.

5.2.4.1 Follow up of Premature Withdrawals (see 7.4)

End of treatment safety visit to be performed 28 days post last dose (Assessments to be performed are according to SoA at 1 year, End of Treatment Safety Assessment in Table 11).

In addition, tumour assessments including CEA in patients withdrawing from treatment before week 25 will be performed at week 25. Subsequent follow up will be done according to Table 11.

5.2.5 Additional Assessments during Treatment Phase

Additional assessments will be required in the event of hypertension, proteinuria, thrombosis and haemorrhagic events as specified below. This enhanced safety monitoring process will be recorded in the CRF.

<u>Hypertension</u>

In the case of Grade 3/4 hypertension, additional blood pressure measurements should be performed on a weekly basis until resolution of the event. Further study treatment should be in line with the recommendations as defined in section 7.3.

<u>Proteinuria</u>

Proteinuria by dipstick has to be assessed according to the SoA unless proteinuria has been determined by 24-hour urine collection.

Proteinuria assessment and further bevacizumab treatment should be in line with recommendation as defined in sections 7.3.2.5, 7.3.6.1, and Appendix 11.

<u>Thrombosis/Embolism</u>

In the event of Grade 3/4 thrombosis a blood sample for the following laboratory parameters should be taken, preferentially prior to initiation of treatment for the event: INR, APTT, D-dimers. Further study treatment should be in line with the recommendations as defined in section 7.3.

<u>Hemorrhagic events</u>

In case of a hemorrhagic event Grade ≥ 2, a blood sample for the following laboratory values should be taken prior to treatment for the event: platelet count, INR, APTT, D-dimers. Further study treatment should be in line with the recommendations as defined in section 7.3.

5.2.6 Additional Assessments during Follow-up Phase

Additional assessments will be required in case of surgical procedures during the first six months of the follow-up phase. This enhanced safety monitoring will be recorded in the CRF.

Wound healing

To date there is limited information on the outcome of surgery after the end of the treatment with bevacizumab. Due to the long half-life of the IgG1 MAb the healing process could potentially be affected beyond the general 28-day follow-up period. Thus, full documentation of surgical procedures performed up to 6 months after the end of study treatment phase and their outcome is requested in order to assess the risk of wound healing disturbances potentially associated with bevacizumab.

5.2.7 Anti-bevacizumab Antibodies (HAHA)

In arms B and C, samples will be taken pre-bevacizumab on day 1 cycle 1, and 1 and 1.5 years after randomization (Appendix 14).

5.2.8 Pharmacoeconomic Assessments

Pharmacoeconomic analyses will be performed on medical care utilization (MCU). The objective will be to summarize and evaluate treatment group differences in total resource use, and more specifically, in resource use associated with (1) treatment of drug-related adverse events and in (2) resource use for the routine drug administration and monitoring associated with study drugs. For treatment of drug-related adverse events, MCU includes number of hospital admissions, total days of hospital use, use of intensive care, number of ambulatory encounters with a physician or other health professional, medications, and laboratory and diagnostic tests associated with these adverse events. For routine therapy, MCU data are similarly defined, but are associated with the routine administration of the treatment group medications. For ambulatory encounters, information on the type of providers seen, the number and duration of encounters as well as the location of the encounter will be recorded. MCU information associated with adverse events will be collected until 28 days after the end of the study treatment phase. MCU information associated with routine therapy will be collected according to the Schedule of Assessments. Analysis of pharmacoeconomic data will be analysed separately and will not be included in the CSR.

5.2.9 Chemotherapy Convenience and Satisfaction Assessment

An assessment of the chemotherapy convenience and satisfaction will be performed. The objective will be to summarize and evaluate treatment group differences in chemotherapy convenience and satisfaction. A chemotherapy convenience and satisfaction questionnaire (CCSQ) will be performed in all patients who can complete it in available languages. This information will be collected every 6 weeks until week 25 during the study treatment period. At baseline only one question on quality of life will be asked. Validation of the CCSQ is currently ongoing in phase III colorectal cancer studies.

The on-therapy CCSQ will take approximately 10 to 15 minutes to complete. A copy of the questionnaire to be used with the associated patient instructions is included in the Appendix 15.

6. INVESTIGATIONAL REGIMENS

In general, for non-medical reasons, deviations of ± 3 days from schedule are permitted.

6.1 Dose and Schedule of Study Medication

Arm A (FOLFOX-4):

Week 1 – 24 (FOLFOX-4): Oxaliplatin will be administered as an 85 mg/m^2 intravenous infusion over 2 hours concomitantly with leucovorin (LV), as a 200 mg/m^2 infusion over 2 hours, followed by 5-fluorouracil (5-FU), given as a 400 mg/m^2 bolus injection, and then as a 600 mg/m^2 continuous infusion over 22 hours. LV 200 mg/m^2 (alone), followed by 5-FU 400 mg/m^2 bolus injection, and 5-FU 600 mg/m^2 continuous infusion are repeated on day 2. Cycle length is 2 weeks comprising approximately 48 hours of infusion and 12 days of rest. Cycles to be repeated every second week for a total of 12 cycles (24 weeks).

Week 25 – 48 : Observation only.

Arm B (FOLFOX-4+Bev):

Week 1 – 24 (FOLFOX-4+Bev):

Bevacizumab (see 6.4.1.1) at 5 mg/kg will be administered as an intravenous infusion over 30 – 90 minutes followed by oxaliplatin, administered as an 85 mg/m^2 intravenous infusion over 2 hours (on day 1 only) concomitantly with leucovorin (LV), as a 200 mg/m^2 infusion over 2 hours, followed by 5-fluorouracil (5-FU), given as a 400 mg/m^2 bolus injection, and then as a 600 mg/m^2 continuous infusion over 22 hours. LV 200 mg/m^2 (alone), followed by 5-FU 400 mg/m^2 bolus injection, and 5-FU 600 mg/m^2 continuous infusion are repeated on day 2. Cycle length is 2 weeks comprising approximately 49 hours of infusion and 12 days of rest. Cycles to be repeated every second week for a total of 12 cycles (24 weeks).

Week 25 – 48 (Bev Monotherapy):

Bevacizumab (see 6.4.1.1) at 7.5 mg/kg will be administered as an intravenous infusion over 30 minutes. Cycle length is 3 weeks. Cycles to be repeated every 3 week for a total of 8 cycles (24 weeks).

Arm C (XELOX+Bev):

Week 1 – 24 (XELOX+Bev):

Bevacizumab (see 6.4.1.1) at 7.5 mg/kg will be administered as an intravenous infusion over 30 – 90 minutes followed by oxaliplatin administered as a 130 mg/m^2 intravenous infusion over 2 hours (day 1 every 3 weeks) in combination with capecitabine, which will be administered orally at a dose of 1000 mg/m^2 twice-daily (equivalent to a total daily dose of 2000 mg/m^2), with first dose the evening of day 1 and last dose the morning of day 15, given as intermittent treatment (3-week cycles consisting of 2 weeks of treatment followed by 1 week without treatment), for a total of 8 cycles (24 weeks).

Week 25 – 48 (Bev Monotherapy):

Bevacizumab (see 6.4.1.1) at 7.5 mg/kg will be administered as an intravenous infusion over 30 minutes. Cycle length is 3 weeks. Cycles to be repeated every 3 week for a total of 8 cycles (24 weeks).

6.1.1 Specification of Units

Doses for capecitabine, oxaliplatin, 5-fluorouracil and leucovorin will be administered on the basis of milligrams of each drug per square meter of body surface area (BSA) as measured at baseline (mg/m^2). A nomogram for the assessment of BSA from the patient's height and weight is provided in Appendix 7. [Doses of capecitabine will be identified from dosing tables Appendix 8]. As outlined in Appendix 8 capecitabine doses will be limited to a maximum BSA of 2.23 m^2. This applies to oxaliplatin as well. Bevacizumab will be administered according to mg/kg up to a maximum of 135 kg body weight. Though the weight of the patient may change throughout the study, BSA will be assumed to stay close to that measured at baseline (i.e. no dose adjustments for changes in body weight will be done unless weight loss alone is considered to be an adverse event of Grade 2 or more). Dose reductions during treatment should be based on adverse events (see Section 7.3).

6.1.2 Study Treatment Duration

The Study Treatment Phase is defined as 48 weeks divided into 2 parts of 24 weeks duration. However, part 2 of Arm A (weeks 25 to 48) consists of observation only. Furthermore, patients with clearly documented recurrence of disease or the diagnosis of a new colorectal cancer must be taken off treatment at the time of diagnosis.

In case one of the treatment combination components is discontinued due to toxicity or patient refusal, remaining study treatment may continue according to detailed guidance provided in Section 7.3.

6.2 Arm A: FOLFOX-4

6.2.1 Week 1 - 24

Cycles are 14 days in length.

Figure 3 Arm A: Week 1-24 FOLFOX-4 dosing schedule

Day 1		Day 2		Days 3-14
Oxaliplatin<br>85 mg/m^2<br>Leucovorin<br>200 mg/m^2		Leucovorin<br>200 mg/m^2		Rest period
H0 → H2	H2 → H24	H24 → H26	H26 → H48	
	5-FU bolus 400 mg/m^2 over 2-4 minutes<br>↓<br>5-FU infusion 600 mg/m^2 over 22 hours		5-FU bolus 400 mg/m^2 over 2-4 minutes<br>↓<br>5-FU infusion 600 mg/m^2 over 22 hours	
If one of the treatment combination components is discontinued due to toxicity, remaining study treatment may continue according to guidance provided in Section 7.3.				

6.2.1.1 Oxaliplatin Dosing Instructions

Oxaliplatin will be administered at the dose of 85 mg/m^2 given as a 2 hour intravenous infusion on day 1 of a two-week cycle. The investigator must calculate the dose using the body surface area (BSA) of the patient (Nomogram, Appendix 7). The dose of oxaliplatin administered should be as close as possible to the calculated dose of 85 mg per m^2 (for details see section 6.6).

Oxaliplatin should always be administered before fluoropyrimidines.

6.2.1.2 Dosing Procedures

Day 1:

- Oxaliplatin 85 mg/m^2 administered on day 1 (only) as a 2-hour IV infusion in 250 mL dextrose 5%, concurrently (via a Y-connector) with
- Leucovorin (LV) 200 mg/m^2 administered as an 2-hour IV infusion in 250 mL dextrose 5%, followed by
- 5-Fluorouracil (5-FU) 400 mg/m^2 as an IV bolus injection (IV push administered by hand), followed by
- 5-Fluorouracil (5-FU) 600 mg/m^2 as a 22-hour IV infusion.

Day 2:

- Leucovorin (LV) 200 mg/m^2 administered as an 2-hour IV infusion in 250 mL dextrose 5%, followed by
- 5-Fluorouracil (5-FU) 400 mg/m^2 as an IV bolus injection, followed by
- 5-Fluorouracil (5-FU) 600 mg/m^2 as a 22-hour IV infusion.

6.2.2 Week 25 - 48

Observation only

6.3 Arm B: FOLFOX-4+Bev

6.3.1 Week 1 - 24

Cycles are 14 days in length.

Figure 4 Arm B Week 1 - 24: FOLFOX-4 + Bev dosing schedule

**Day 1**			**Day 2**		**Days 3-14**
Bevacizumab 5 mg/kg	Oxaliplatin 85 mg/m^2<br>Leucovorin 200 mg/m^2		Leucovorin 200 mg/m^2		Rest period
H0 → H1	H1 → H3	H3 → H25	H25→H27	H27 → H49	
		5-FU bolus 400 mg/m^2 over 2-4 minutes<br>↓<br>5-FU infusion 600 mg/m^2 over 22 hours		5-FU bolus 400 mg/m^2 over 2-4 minutes<br>↓<br>5-FU infusion 600 mg/m^2 over 22 hours	
If one of the treatment combination components is discontinued due to toxicity study treatment may continue according to guidance provided in Section 7.3.					

6.3.1.1 Dosing instructions for Bevacizumab

Bevacizumab will be administered at the dose of 5 mg/kg as an intravenous infusion over a 30(±10) to 90(±15) minute period prior to oxaliplatin on day 1 of a two week cycle. Bevacizumab will be administered initially over 90 (±15) minutes. If the first infusion is tolerated without infusion-associated adverse events (fever and/or chills), the second infusion may be delivered over 60 (±10) minutes. If the 60-minute infusion is well tolerated, all subsequent infusions may be delivered over 30 (±10) minutes (for details see section 6.5).

6.3.1.2 Oxaliplatin Dosing Instructions

Oxaliplatin will be administered at the dose of 85 mg/m^2 given as a 2 hour intravenous infusion on day 1 of a two-week cycle, after bevacizumab. The investigator must calculate the dose using the body surface area (BSA) of the patient at baseline (Nomogram, Appendix 7). The dose of oxaliplatin administered should be as close as possible to the calculated dose of 85 mg per m^2 (for details see section 6.6).

Oxaliplatin should always be administered before fluoropyrimidines.

6.3.1.3 Dosing Procedures

Day 1:

- Bevacizumab 5 mg/kg administered on day 1 (only) as an infusion over 30±10 to 90±15 minutes followed by
- Oxaliplatin 85 mg/m² administered on day 1 (only) as a 2-hour IV infusion in 250 mL dextrose 5%, concurrently (via a Y-connector) with
- Leucovorin (LV) 200 mg/m^2 administered as an 2-hour IV infusion in 250 mL dextrose 5%, followed by
- 5-Fluorouracil (5-FU) 400 mg/m² as an IV bolus injection (IV push administered by hand), followed by
- 5-Fluorouracil (5-FU) 600 mg/m² as a 22-hour IV infusion.

Day 2:

- Leucovorin (LV) 200 mg/m^2 administered as an 2-hour IV infusion in 250 mL dextrose 5%, followed by
- 5-Fluorouracil (5-FU) 400 mg/m² as an IV bolus injection, followed by
- 5-Fluorouracil (5-FU) 600 mg/m² as a 22-hour IV infusion.

6.3.2 Week 25 - 48

Cycles are 21 days in length.

Figure 5 Arm B Week 25- 48: Bev dosing schedule

Day 1	Days 2-21
Bevacizumab 7.5 mg/kg	
H0 → H0.5	
	Rest Period

6.3.2.1 Dosing instructions for Bevacizumab

Bevacizumab will be administered at the dose of 7.5 mg/kg as an intravenous infusion over a 30(±10) minute period.

6.3.2.2 Dosing Procedures

Day 1:

Bevacizumab 7.5 mg/kg administered on day 1 (only) as an infusion over 30±10 minutes.

6.4 Arm C: XELOX+Bev

6.4.1 Week 1 - 24

Cycles are 21 days in length.

Figure 6 Arm C Week 1 - 24: XELOX + Bev dosing schedule

Day 1			Days 2-14	Day 15	Days 16-21
Bevacizumab 7.5 mg/kg	Oxaliplatin 130 mg/m^2				
H0 → H1	H1 → H3	PM	AM PM	AM	
		Capecitabine (once every 12 hours) ← →			Rest period
If one of the treatment combination components is discontinued due to toxicity study treatment may continue according to guidance provided in Section 7.3.					

6.4.1.1 Dosing instructions for Bevacizumab

Bevacizumab will be administered at the dose of 7.5 mg/kg as an intravenous infusion over a 30(±10) to 90(±15) minute period prior to oxaliplatin on day 1 of a three week cycle. Bevacizumab will be administered initially over 90(±15) minutes. If the first infusion is tolerated without infusion-associated adverse events (fever and/or chills), the second infusion may be delivered over 60(±10) minutes. If the 60-minute infusion is well tolerated, all subsequent infusions may be delivered over 30(±10) minutes (for details see section 6.5).

6.4.1.2 Oxaliplatin Dosing Instructions

Oxaliplatin will be administered at the dose of 130 mg/m^2 given as a 2 hour intravenous infusion on day 1 of a three week cycle, after bevacizumab, and prior to the first dose of capecitabine. The investigator must calculate the dose using the body surface area (BSA) of the patient at baseline (Nomogram, Appendix 7). The dose of oxaliplatin administered should be as close as possible to the calculated dose of 130 mg per m^2 (for details see section 6.6).

Oxaliplatin should always be administered before fluoropyrimidines.

6.4.1.3 Capecitabine Dosing Instructions

The appropriate daily dose of capecitabine is identified by the following steps:

1. Determine the Body Surface Area:
    - Measure the patient's actual height and weight
    - Derive the BSA using the nomogram found in Appendix 7.
2. Look up the specific dose:

Based on the derived BSA, find the dose to be used by looking it up in the capecitabine dosing tables in Appendix 8 (dose not to be calculated).

6.4.1.4 Route of Administration

Capecitabine is to be administered orally within 30 minutes after the end of a meal (breakfast, dinner). Tablets should be swallowed with approximately 200 mL water (not fruit juices). The first dose of each cycle will be administered as the evening dose on day 1 and the last dose of each cycle is scheduled the morning of day 15, followed by a 7 day rest period. This provides for a total of 28 single doses per cycle over 15 calendar days.

6.4.2 Week 25 - 48

Cycles are 21 days in length.

Figure 7 Arm C Week 25- 48: Bev dosing schedule

Day 1	Days 2-21
Bevacizumab 7.5 mg/kg	
H0 → H0.5	
	Rest Period

6.4.2.1 Dosing instructions for Bevacizumab

Bevacizumab will be administered at the dose of 7.5 mg/kg as an intravenous infusion over a 30(±10) minute period.

6.4.2.2 Dosing Procedures

Day 1:

Bevacizumab 7.5 mg/kg administered on day 1 (only) as an infusion over 30±10 minutes.

6.5 General Bevacizumab Dosing Instructions

Bevacizumab doses will be calculated for each patient in milligrams per kilogram (mg/kg). The patient's actual weight from the baseline visit will be the reference weight throughout the study (i.e., patients will receive the same dose at each treatment). Bevacizumab will be administered up to a maximum body weight of 135 kg. Doses of bevacizumab will be recalculated for patients who experience more than 10% change in body weight from baseline during the treatment period.

6.5.1 Permitted Treatment Interruptions

Note: To ensure the success of the therapy it is essential that patients comply with the treatment schedule. Every effort has to be made in order to avoid a situation that a patient misses more than

- 3 consecutive administrations of bevacizumab in arm B during part 1 of the study treatment phase (week 1-24)
- 2 consecutive administrations of bevacizumab in arm B during part 2 of study treatment phase (week 25-48)
- 2 consecutive administrations of bevacizumab in arm C.

6.5.2 Dosage and Method of Administration

Bevacizumab should be prepared by a healthcare professional using aseptic technique.

All patients will receive an infusion of study drug in a total volume of 100 mL of 0.9% Sodium Chloride Injection, USP. For example, a 50-kg patient receiving 5.0 mg/kg should receive 10.0 mL of study drug solution; this is added to 90.0 mL of 0.9% Sodium Chloride Injection, USP, resulting in an end volume of 100 mL. In contrast, a 50-kg patient receiving 7.5 mg/kg should receive 15.0 mL of study drug solution; this is added to 85.0 mL of 0.9% Sodium Chloride Injection, USP, resulting in an end volume of 100 mL. Only 0.9% Sodium Chloride Injection, USP, may be used for further dilution. Once bevaizumab has been added to a bag of sterile saline, the solution must be administered within 8 hours.

Bevacizumab infusions should not be administered or mixed with dextrose or glucose solutions [please refer to the *Investigators Brochure*].

The initial study drug dose will be delivered over 90 minutes as a continuous IV infusion. If the first infusion is tolerated without infusion-associated adverse events the second infusion may be delivered over 60 minutes. If the 60-minute infusion is tolerated, all

subsequent infusions may be delivered over 30 minutes. If a patient experiences infusion-associated adverse events with the 60-minute infusion, all subsequent doses will be given over 90 minutes. If a patient experiences infusion-associated adverse events with the 30-minute infusion, all subsequent doses will be given over 60 minutes. A rate-regulating device will be used for all study drug infusions. When the study drug IV bag is empty, 50 mL of 0.9% Sodium Chloride Injection, USP, will be added to the IV bag or an additional bag will be hung, and the infusion will be continued for a volume equal to that of the tubing to ensure complete delivery of the study drug. The total infusion time, therefore, should always be either 90, 60, or 30 minutes. If more saline is infused, the extent of saline infusion does not factor into the study drug infusion time.

Do not administer as an intravenous push or bolus.

Should *extravasation* of the study drug infusion occur, the following steps are to be taken:

1. Discontinue the IV.
2. If a significant volume of the study drug infusion remains, restart the IV at a more proximal site in the same limb.
3. Treat the infiltration according to institutional guidelines for infiltration of a noncaustic agent.

6.6 General Oxaliplatin Dosing Instructions

Oxaliplatin dose will be calculated using the body surface area (BSA) of the patient (Nomogram, Appendix 7). The dose of oxaliplatin administered should be as close as possible to the calculated dose. Oxaliplatin will be limited to a maximum BSA of 2.23 m^2.

Oxaliplatin administration does not require hyperhydration. In the event of extravasation, administration must be discontinued immediately.

For nausea and vomiting, 5-HT3 antagonists with or without dexamethasone are strongly recommended for oxaliplatin-based chemotherapy.

Oxaliplatin must be infused either by peripheral vein or central venous line over 2 hours. The infusion line must be adequately flushed with 5% dextrose solution (D5W) between oxaliplatin infusion and the administration of any other drug. If desired, oxaliplatin can be administered concurrently (via a Y-connector from separate bags and lines) with leucovorin, given as a 2-hour IV infusion in 250 mL dextrose 5%, but should not be administered concurrently with 5-FU. See section 6.7.2 for preparation details.

6.7 Preparation and Administration of Test Drug and Comparator(s)

Study drugs will be supplied as detailed below:

– Bevacizumab is supplied as 5-mL glass vials with 4-mL fill, containing 100 mg (25 mg/mL). Vials contain no preservative and are for single use only.

– Oxaliplatin (Eloxatin®) is supplied in vials containing 50 mg or 100 mg of oxaliplatin as a sterile, preservative-free lyophilized powder for reconstitution.

– 5-Fluorouracil (5-FU) will be obtained locally by prescription.

– Leucovorin (LV) will be obtained locally by prescription.

– Capecitabine (Xeloda®) is supplied as film-coated tablets of 150 mg and 500 mg, packed in polyethylene bottles containing 60 tablets of the 150 mg strength, and 120 tablets of the 500 mg strength. The tablets are not scored and should not be split.

The study drugs will be labeled with the standard text as described below in Figure 8:

Figure 8 Master Label for the Study Drugs

Protocol number BO17920<br>Generic Name<br>Roformis number<br>Quantity<br>Dosage/strength/potency<br>Dosage form<br>Retest date:<br>Lot number<br>Storage conditions<br>Direction for use<br>Caution statements<br><br>Sponsor

NOTE: Text might be in different order on the labels
Text will be adapted to the country specific local requirements

6.7.1 Preparation of Bevacizumab

Bevacizumab should be prepared by a healthcare professional using aseptic technique.

Vials are for single use only and once added to a bag of sterile saline, must be used within 8 hours. Bevacizumab infusions should not be administered or mixed with dextrose or glucose solutions. No incompatibilities between bevacizumab and polyvinyl chloride or polyolefin bags have been observed [please refer to the *Investigators Brochure*].

For administration, bevacizumab will be diluted in 0.9% Sodium Chloride Injection, USP to a total volume of 100 mL.

6.7.2 Preparation of Oxaliplatin

6.7.2.1 Reconstitution of Oxaliplatin

RECONSTITUTION OR FINAL DILUTION MUST NEVER BE PERFORMED WITH SODIUM CHLORIDE SOLUTION OR OTHER CHLORIDE-CONTAINING SOLUTIONS.

The lyophilized powder is reconstituted by adding 10 mL (for the 50 mg vial) or 20 mL (for the 100 mg vial) of Water for Injection, or dextrose 5% in water for injection (D5W). Do not administer the reconstituted solution without further dilution. The reconstituted solution must be further diluted in an infusion solution of 250 to 500 mL of D5W.

Dispose of any reconstituted solution that shows evidence of precipitation. Always use the recommended solvents.

6.7.2.2 Incompatibilities/Precautions

Oxaliplatin is incompatible in solution with alkaline medications or media (such as basic solutions of 5-FU) and must not be mixed with these or administered simultaneously through the same infusion line. The infusion line should be flushed with D5W prior to administration of any concomitant medication. Do not simultaneously administer other drugs by the same infusion line, except for leucovorin.

Needles or intravenous administration sets containing aluminum parts that may come in contact with oxaliplatin should not be used for the preparation or mixing of the drug. Aluminum has been reported to cause degradation of platinum compounds.

Flush line after oxaliplatin administration. Flush lines with a 5% dextrose solution after oxaliplatin is given and before administration of supportive care medications.

6.7.3 5-FU and Leucovorin

Fluorouracil solutions may be incompatible with synthetic elastomers; microscopic precipitation has been reported as soon as 4 hours after placement into polyisoprene reservoirs of elastomeric infusers and in polypropylene syringes with an elastomeric joint. 5-FU and leucovorin should be reconstituted according to local practice, following the guidelines in the local labels for the two products.

6.8 Storage Conditions

All study drugs must be stored in a locked facility in a dry place and out of the reach of children.

6.8.1 Capecitabine Storage

Tablets may be stored at room temperature 15° to 30° C. (59° to 86° F).

6.8.2 Bevacizumab Storage

Upon receipt of bevacizumab, vials are to be refrigerated at 2° to 8°C (36° to 46°F) and should remain refrigerated until just prior to use. DO NOT FREEZE. DO NOT SHAKE. Opened vials must be used within 8 hours. VIALS ARE FOR SINGLE USE ONLY.

Bevacizumab is not packaged patient specific. Unused vials from one box can be distributed to another patient.

6.8.3 Oxaliplatin Storage

- Unreconstituted vials may be stored at room temperature (25°C (77°F); excursions permitted from 15° to 30°C (59°-86°F).
- After reconstitution in the original vial, the solution may be stored up to 24 hours under refrigeration [2-8°C (36-46° F)].
- After final dilution with 250-500 mL of 5% Dextrose Injection, the shelf life is 6 hours at room temperature [20-25°C (68- 77°F)] or up to 24 hours under refrigeration [2-8°C (36-46°F)].
- Oxaliplatin is not light sensitive.

6.8.4 5-FU and Leucovorin Storage

A solution in 5% glucose is stable for at least 16 weeks when stored in polyvinyl chloride PVC at 5°C (41°F). When stored at room temperature in PVC, solutions of fluorouracil may be subject to evaporative loss of water, which slowly increases their concentration. Commercial solutions of fluorouracil for injection have been reported to be stable for 7 days at 37°C (98.6°F) in a portable infusion pump. Caution must be applied with regard to the possibility of precipitation or microbiological contamination. For stability of leucovorin please refer to the local package insert.

6.9 Blinding and Randomization

This is an open label study. Patients will be randomly assigned to one of the three treatment arms using a block design randomization procedure stratified according to AJCC/UICC stage [high-risk stage II (see 4.2) vs. stage III N1 vs. stage III N2 (Appendix 5)] and geographic region (United States, Central and South America, Australia/New Zealand/Canada, West Europe, Central East Europe, South East Europe, East Asia, South East Asia/ South Africa). Details are provided in the IVRS manual. It should be noted that, if necessary, new countries could be added to existing regions. Once 570 high-risk stage II patients have been randomized, recruitment for high-risk stage II patients will stop and only stage III patients are further allowed to enter the trial.

As this is an open label study there will be no blinding of treatment assignment.

Randomization numbers will be chronologically assigned as patients are enrolled in the study. All patient numbers across the study will be unique.

Randomization will be performed via a central randomization service (IVRS) available 24 hours a day, seven days a week. Dedicated telephone numbers for IVRS will be provided to each study site (refer to the *IVRS Manual*). During the process of randomization, stratification will be according to the geographic region and AJCC/UICC stage (high-risk stage II vs. stage III N1 vs. stage III N2). This information will be required from the investigators at the time of contacting the IVRS.

All patients must commence treatment within 7 days of randomization.

6.10 Drug Accountability

A Drug Dispensing Log for bevacizumab and oxaliplatin must be kept current and should contain the following information:

- the identification of the patient for whom the drug was administered / dispensed to.
- the date(s) and the quantity of the drug provided to the investigational site

This inventory must be available for inspection by the Roche Monitor. All supplies, including partially used or empty containers must be fully documented in the Drug Dispensing Log and will be verified by the Roche Monitor.

Drug supplies (partly used and unused medication) must be available for inspection, at every monitoring visit. After the monitor has reconciled supplies unused supplies will be destroyed according to site specific procedures. When requested in writing to the Sponsor – unused drug supplies (if not classified as controlled substances) may be destroyed by the investigator provided such disposition does not expose humans to risks from the drug. Records shall be maintained by the investigator of any such disposition of the test drug. These records must show the identification and quantity of each unit disposed of, the method of destruction (taking into account the requirements of the local law), and the person who disposed of the test substance. Such records shall be submitted to the Sponsor.

6.10.1 5-FU/LV accountability (arms A and B)

The clinic administration records will be used to monitor compliance with 5-FU and leucovorin.

6.11 Capecitabine Compliance (arm C)

A preprinted drug dispensing log is provided by the Sponsor. It must be kept current and should contain the following information:

– Identification of the patient to whom the drug was dispensed;

– Date(s) and quantity of the drug dispensed to the patient;

– Date(s) and quantity of the drug returned by the patient.

The inventory must be available for inspection by the Clinical Trial Monitor. All unused capecitabine medication must be returned by the patients to the investigator at the beginning of the next cycle.

Capecitabine medication supplies (empty containers, as well as partly used and unused medication) must be available for inspection, at every monitoring visit. After the monitor has reconciled supplies, arrangements may be made to destroy unneeded supplies to the Sponsor.

The investigator should verify compliance by counting the returned (if any) tablets and should encourage the patient to take the required doses of capecitabine according to the treatment plan. Therefore the investigator needs to encourage the patient to return all unused medication including all bottles dispensed (partly used or empty) to perform a tablet count.

6.11.1 Dispensing of capecitabine

Starting with the first day of cycle 1, and continuing with day 1 of every subsequent cycle, study staff should:

– Note the pill count of each strength tablet given to the patient for the administration of drug during the subsequent cycle,

– Record this information (i.e. the numbers of each strength tablet dispensed) in the CRF page for capecitabine study medication, as well as in the drug dispensing log,

Instruct the patient to follow the treatment schedule and return all unused tablets, along with all capecitabine bottles at every visit, regardless if the bottles are empty or containing tablets.

6.11.2 Accountability of capecitabine supply

The investigator or research nurse should verify compliance at every visit. Starting with the first day of the second cycle, and preceding with the first day of every subsequent cycle, study staff should:

– Discuss the number of capecitabine tablets taken each day by the patient over the previous cycle,

– Record this information in the CRF on the Capecitabine Treatment Page,

– Count the tablets of each strength returned by the patient,

– Enter the number of returned tablets in the Capecitabine Compliance section of the CRF,

– Determine if the patient took all planned study medication for the preceding cycle,

Document (and comment on) any discrepancy between the medication returned and what the patient took (as per Capecitabine Treatment Page) on the capecitabine returns CRF page for that visit. Further, if necessary make corrections to the treatment log in accordance with the determined capecitabine usage.

7. SAFETY INSTRUCTIONS AND GUIDANCE

7.1 Adverse Events and Laboratory Abnormalities

7.1.1 Clinical Adverse Events

An Adverse Event is any untoward medical occurrence in a patient or clinical investigation patient administered a pharmaceutical product and which does not necessarily have to have a causal relationship with this treatment. An Adverse Event can

therefore be any unfavorable and unintended sign (including an abnormal laboratory finding, for example, see Section 7.1.2), symptom, or disease temporarily associated with the use of a medicinal product, whether or not considered related to the medicinal product. Pre-existing conditions which worsen during a study are to be reported as Adverse Events.

All clinical adverse events (AEs) encountered during the clinical study will be reported on the AE page of the CRF. Intensity of adverse events will be graded using the National Cancer Institute Common Terminology Criteria for Adverse Events (version 3.0) (CTCAE v 3.0 (http://ctep.cancer.gov/reporting/ctc.html) [89]) and reported in detail as indicated on the CRF. If an adverse event occurs which is not contained in the CTCAE v 3.0, the five-point scale below will be used.

Mild	Discomfort noticed but no disruption of normal daily activity.
Moderate	Discomfort sufficient to reduce or affect daily activity.
Severe	Inability to work or perform normal daily activity
Life Threatening	Represents an immediate threat to life
Death	Related to AE

Relationship of the adverse event to treatment should also be assessed. Description of scales can be found in Appendix 1.

Targeted events as defined in this study include hypertension, proteinuria and wound healing disturbances. For details please refer to sections 7.2.3.2 and 7.5.1.

7.1.2 Laboratory Test Abnormalities

Laboratory test results will be recorded on the laboratory results pages of the CRF. Laboratory test value abnormalities as such should not be reported on the AE page of the CRF as adverse events, unless they are treatment-emergent and they satisfy one or more of the following conditions of clinical significance:

- Accompanied by clinical symptoms,
- Lead to a change in study medication (e.g. dose modification, interruption or permanent discontinuation),
- Require a change in concomitant therapy (e.g. addition, interruption, discontinuation or any other change of concomitant therapy or treatment).

Please Note: any abnormal laboratory result fulfilling the criteria for a Serious Adverse Event (SAE) should be reported as such, in addition to being recorded as an adverse event in the CRF.

7.2 Handling of Safety Parameters

7.2.1 Serious Adverse Events

7.2.1.1 Immediately Reportable to Roche

Any clinical adverse event or abnormal laboratory test value that is *serious* occurring after the patient signed the informed consent form up to 28 days after the end of the study treatment phase, irrespective of the treatment received by the patient, must be reported to Roche within *one* working day of occurrence.

All related SAEs or related serious lab abnormalities that occurred at any time following study discontinuation or completion are immediately reportable to Roche.

The definition and reporting requirements of ICH Guideline for Clinical Safety Data Management, Definitions and Standards for Expedited Reporting, Topic E2 and the EU Clinical Trial Directive will be adhered to. Complete information can be found in Appendix 3 and Appendix 4.

7.2.1.2 Project Specific Adverse Event Definition

Progression or deterioration of the malignancy under study (including new metastatic lesions and death due to disease progression) will be part of the efficacy assessment and should NOT be reported as AE/SAE.

Signs and *symptoms* of the malignancy under study should only be reported if:

- Newly emergent (i.e. not present at baseline) and the association with the underlying malignancy and old/new metastatic lesions is unclear and/or
- The investigator attributed deterioration of malignancy-associated signs and symptoms directly to the study drug.

Should there be any uncertainty regarding the attribution of the malignancy under study to an AE, it should be reported as an AE or a SAE accordingly.

7.2.2 Targeted Events

Proteinuria and hypertension have been observed in all bevacizumab clinical trials conducted to date. In addition, post-operative wound healing complications have been observed in both animal studies and in patients who underwent major surgeries during the treatment with bevacizumab in various clinical trials.

In order to get a better understanding of the underlying pathogenesis, and to define the at risk patient population, specific enhanced safety reporting processes for these targeted events occurring during the study have been implemented. This procedure is intended to improve the management of these targeted events (see Section 7.2.3.2).

7.2.3 Reporting and Follow-up of Adverse Events after the End of Study Treatment Phase

7.2.3.1 Adverse Events in general

All new adverse events encountered up till 28 days after the last dose of study treatment/end of the observation phase (arm A) should be recorded in the AE page of the CRF.

Related non-serious new AEs occurring up to 6 months after the last dose of study drug/end of the observation phase (arm A) should be reported.

Related serious adverse events (SAEs) should be reported indefinitely.

Adverse events, especially those for which the relationship to test drug is “related”, should be followed up until they have returned to baseline status or stabilized or the causal relationship has been changed.

All unrelated, mild or moderate events must be followed up to 28 days after the last dose of the study treatment/end of the observation phase (arm A).

All severe, life-threatening or related events must be followed up until resolution or the causal relationship has been changed or the patient’s death.

7.2.3.2 Targeted Events – Reporting and Follow-up Procedure

New hypertension events, irrespective of severity and causal relationship, should be reported up till 6 months after the end of the study treatment phase or up till 6 months after the last dose of study drug in case of early termination. Patients who have ongoing *hypertension* at the termination or early termination visit or who experience a new hypertensive event up to 6 months after the end of study treatment phase will have their blood pressure and use of anti-hypertensives monitored every 3 months for up to one year or until blood pressure returns to within normal range (systolic BP $\leq$ 140 mmHg and diastolic BP $\leq$ 90 mmHg).

New proteinuria events, irrespective of severity and causal relationship, should be reported up till 6 months after the end of the study treatment phase or up till 6 months after the last dose of study drug in case of early termination. Patients who have ongoing *proteinuria* events at the termination or early termination visit or who experience a new proteinuria event up to 6 months after the end of study treatment phase will be monitored for 24-hour urine collection every 3 months for up to one year or until total 24-hour urine protein improves to $\leq$ 1g.

All surgical procedures that occurred within 6 months of the end of the study treatment phase or within 6 months after the last dose of study drug in case of early termination, with or without wound healing complications, should be documented in the CRF. If associated post-operative wound healing complications occurred, these should be reported as adverse events. Patients who have ongoing *wound healing complications* at the termination or early termination visit or who experience a new wound healing related event up to 6 months after the end of study treatment phase will have their wound healing complications monitored every 3 weeks until the events have resolved.

7.2.3.3 Treatment of Adverse Events

Adverse events should be treated according to local practice. Diagnostic and interventional procedures and concomitant medication have to be recorded on the CRF.

7.2.4 Follow-up of Abnormal Laboratory Test Values

In the event of unexplained abnormal laboratory test values, the tests should be repeated and followed up until they have returned to the normal range or to the baseline value, and/or an adequate explanation of the abnormality is found. If a clear explanation is established it should be recorded on the CRF. For the follow up of proteinuria please refer to 7.2.3.2.

7.2.5 Pregnancy

Bevacizumab has been shown to be embryotoxic and teratogenic when administered to rabbits. Observed effects included decreases in maternal and foetal body weights, an increased number of foetal resorptions and an increased incidence of specific gross and skeletal foetal alterations. Adverse foetal outcomes were observed at all tested doses of 10–100 mg/kg.

Angiogenesis has been shown to be critically important to foetal development. The inhibition of angiogenesis following administration of bevacizumab could result in an adverse outcome of pregnancy.

There are no adequate and well-controlled studies in pregnant women. IgGs are known to cross the placental barrier, and bevacizumab may inhibit angiogenesis in the foetus. Therefore, bevacizumab should not be used during pregnancy. In women with childbearing potential, appropriate contraceptive measures are recommended during bevacizumab therapy. Based on pharmacokinetic considerations, contraceptive measures are recommended for at least 6 months following the last dose of bevacizumab.

A female patient must be instructed to stop taking any of the test drugs and immediately inform the investigator if she becomes pregnant during the study. Pregnancies occurring up to 6 months after the last dose of the study treatments must also be reported to the investigator. The investigator should report all pregnancies within 1 working day to the sponsor. The investigator should counsel the patient; discuss the risks of continuing with the pregnancy and the possible effects on the fetus. Monitoring of the patient should continue until conclusion of the pregnancy. The patient should be withdrawn from treatment and enter the follow-up phase of the study.

A female partner of patient must be instructed to immediately inform the investigator if a pregnancy is detected. Pregnancies occurring up to 90 days after the last dose of the study treatments must also be reported to the investigator. The investigator should report all pregnancies to the sponsor within 1 working day after written consent is obtained from the pregnant partner. The investigator should counsel the patient and his partner; discuss the risks of continuing with the pregnancy and the possible effects on the fetus. Monitoring of the patient's partner should continue until conclusion of the pregnancy.

7.3 Dose Modifications for Toxicity

7.3.1 General Notes Regarding Dose Modifications

Descriptions of the expected toxicities and dose-reduction tables are contained in each section by treatment arm. Toxicity will be graded according to the CTCAE Version 3.0 [89].

Guidelines to be followed in the case of a deviation from treatment schedule (i.e. treatment interruption or dose modification due to an adverse event) are specific for each treatment regimen, and are described in the following subsections. Reasons for deviation from treatment schedule, the supportive measures taken, and the outcome will be documented in the patient's chart and recorded in the CRF.

– For any adverse event already apparent at baseline, the dose modifications will apply according to the corresponding shift in toxicity grade, if the investigator feels it is appropriate. For example, if a patient has Grade 1 asthenia at baseline which increases to Grade 2 during treatment, this will be considered a shift of one Grade and treated as Grade 1 toxicity for dose modification purposes.

– For toxicities which are considered by the investigator unlikely to develop into serious or life-threatening events (e.g. alopecia, altered taste etc.), treatment will be continued at the same dose without reduction or interruption. In addition, no dose reductions or interruptions will be required for anemia (non-hemolytic) as it can be satisfactorily managed by transfusions.

– Where several toxicities with different grades or severity occur at the same time, the dose modifications applied should be the greatest reduction applicable.

– If, in the opinion of the investigator, a toxicity is considered to be due solely to one drug (e.g. hand-foot syndrome secondary to capecitabine, neurotoxicity due to oxaliplatin, hypertension and proteinuria due to bevacizumab), the dose of the other drugs does not require modification.

– There will be no dose modification of bevacizumab during this study unless the patient's weight changes by more than 10%, in which case the dose will be recalculated. However, the dosing schedule of bevacizumab will be interrupted in the event of certain grades of haemorrhage, thromboembolic events, hypertension, proteinuria, gastro-intestinal perforations, wound healing complications, fistula or intra-abdominal abscess, and infusion-related or allergic reactions, as summarized in Table 12.

– If an extended rest period (i.e. delay, see Appendix 9) is related to either oxaliplatin, capecitabine or 5-FU/LV treatment alone is required, the other study drugs should be delayed as well (except in the case of oxaliplatin-related neurological adverse events; see Section 7.3.6.2). For arms B and C, bevacizumab should be restarted only when the requirements for restarting chemotherapy (capecitabine plus oxaliplatin, or 5-FU/LV plus oxaliplatin, or capecitabine or 5-FU/LV if oxaliplatin was previously discontinued) are met.

- Dose modifications for isolated abnormal hematologic lab values will be based on hematological parameters *at start of a treatment cycle*. There is no scheduled sampling during a treatment cycle and thus, no scheduled collection of nadir values.
- If toxicity requires an extended rest period of all study drugs of more than three additional weeks, the patient will be withdrawn from the study treatment phase for toxicity reasons.
- If capecitabine or 5-FU/LV must be discontinued permanently due to toxicity, the patient will be allowed to continue with bevacizumab but not with oxaliplatin. Patients may not continue on oxaliplatin monotherapy or oxaliplatin plus bevacizumab, at any stage of the study.
- If bevacizumab must be discontinued permanently due to toxicity, the patient will be allowed to continue with the previous regimen of capecitabine plus oxaliplatin, or 5-FU/LV plus oxaliplatin, or capecitabine or 5-FU/LV if oxaliplatin was previously discontinued.

7.3.2 Bevacizumab Toxicities (applicable to arms B and C)

Life threatening toxicities seen with bevacizumab to date have been hemorrhage, thromboembolic events and gastro-intestinal perforation.

Less severe toxicities include proteinuria, hypertension, wound healing complications, diarrhoea, nausea, pain, asthenia and epistaxis. Because of the long terminal half-life of bevacizumab (20 days) the discontinuation of treatment in case of an adverse events is not expected to influence its short-term clinical evolution and therefore, the management of adverse events is based on institution of adequate treatment.

This section provides guidance for treatment modifications due to toxicity. For details about specific adverse event please refer to Section *Bevacizumab Precautions* [7.5.1].

7.3.2.1 Treatment modifications in case of events attributable to bevacizumab

No dose reduction of bevacizumab is foreseen for an individual patient.

In general toxicity attributable to bevacizumab [see below for specific instructions regarding grading and management of haemorrhage (section 7.3.2.2), thromboembolic events (section 7.3.2.3), hypertension (section 7.3.2.4), proteinuria (section 7.3.2.5), gastro-intestinal perforations (section 7.3.2.6), wound healing complications (section 7.3.2.7), fistula or intra-abdominal abscess (section 7.3.2.8), and infusion-related or allergic reactions (section 7.3.2.9)] will require bevacizumab treatment to be held or withdrawal of the patient from study treatment. Section 7.3.2.10 provides a summary of schedule modification of bevacizumab.

Missed doses of bevacizumab will not be made up.

In order to continue with the treatment with bevacizumab after an interruption of more than

- 3 consecutive administrations of bevacizumab in arm B during part 1 of the study treatment phase (week 1-24)
- 2 consecutive administrations of bevacizumab in arm B during part 2 of study treatment phase (week 25-48)
- 2 consecutive administrations of bevacizumab in arm C,

the investigator has to provide a rational to the medical monitor (i.e. reason for extended rest and patient's interest to resume treatment) before approval of continuation.

Any patient who develops any one of the following toxicities attributable to bevacizumab should not receive further bevacizumab:

– Gastrointestinal perforation,

– Arterial thromboembolic events,

– Grade 3/4 haemorrhagic events,

– Symptomatic Grade 4 venous thromboembolic events,

– Grade 4 hypertension (hypertensive crisis),

– Grade 4 proteinuria (nephrotic syndrome).

7.3.2.2 Hemorrhage

All toxicity will be graded according to CTCAE v 3.0 guidelines. If a Grade 3/4 bleeding occurs, appropriate treatment should be instituted and bevacizumab treatment will be discontinued permanently.

7.3.2.3 Thrombosis/Embolism

All toxicity will be graded according to CTCAE v 3.0 guidelines. Patients who develop the following Grades of thrombosis/embolism must discontinue bevacizumab and the following action is recommended:

- Bevacizumab should be permanently discontinued in patients who develop any Grade of arterial thromboembolic event.
- Venous thromboembolic event – Grade 3 or incidentally discovered pulmonary embolus (first occurrence): hold bevacizumab for 2 weeks. Bevacizumab may be resumed after initiation of therapeutic-dose anticoagulant therapy as soon as all of the following criteria are met:

– The patient must be on a stable dose of anticoagulant and, if on an oral coumarin-derivative, have an INR within the target range (usually between 2 and 3) prior to restarting study drug treatment,

– The patient must not have had a Grade 3 or 4 haemorrhagic event since entering the study.

- Symptomatic Grade 4 venous thromboembolic event (first occurrence) - permanently discontinue bevacizumab.

7.3.2.4 Hypertension

Patients should be monitored for the development or worsening of hypertension via frequent blood pressure measurement. Blood pressure measurements should be taken after the patient has been in a resting position for ≥ 5 minutes. Repeated measurement of blood pressure for verification should be undertaken if the initial reading is ≥ 140 mmHg systolic and/or ≥ 90 mmHg diastolic blood pressure. All toxicity will be graded according to CTCAE v 3.0 guidelines:

- Grade 1 hypertension: Asymptomatic, transient (< 24 hrs) increase by > 20 mmHg (diastolic) or to > 150/100 mmHg if previously within normal limits.
  Intervention not indicated.
- Grade 2 hypertension: Recurrent or persistent (> 24 hr) or symptomatic increase by > 20 mmHg (diastolic) or to > 150/100 mmHg if previously within normal limits.
  Monotherapy of anti-hypertensive may be indicated. Once controlled to <150/100 mmHg, patients may continue bevacizumab therapy.
- Grade 3 hypertension: Requiring more than one anti-hypertensive or more intensive therapy than previously.
  Bevacizumab should be withheld for persistent or symptomatic hypertension and should be permanently discontinued if hypertension is not controlled.
- Grade 4 hypertension: Life threatening consequence (e.g. hypertensive crisis).
  Occurrence of Grade 4 hypertension should lead to permanent discontinuation of bevacizumab. All doses of anti-hypertensive medicines should be recorded at all visits.

7.3.2.5 Proteinuria

Patients in all study arms will have a dipstick urinalysis according to the SoA unless proteinuria has been determined by 24-hour urine collection.

All toxicity will be graded according to CTCAE v 3.0 guidelines.

Proteinuria assessment and adjustment of bevacizumab administration for proteinuria should be in line with recommendation as defined below, in Appendix 11 and in Section 7.3.6.1.

- First occurrence of proteinuria:
  - 1+ (dipstick): Administer bevacizumab as scheduled, NO additional evaluation is required.
  - 2+ , 3+ and 4+ proteinuria (dipstick): Administer bevacizumab as scheduled and collect 24-hour urine to determine the total protein within 3 days prior to the next scheduled dose is required:
    - 24-hour proteinuria ≤ 2 g: Administer bevacizumab as scheduled.
    - 24-hour proteinuria > 2 g: Bevacizumab treatment should be withheld pending next 24 hour total protein.

- Repeat 24-hour urine protein ≤ 2 g: Administer bevacizumab as schedule. 24-hour protein should be further monitored prior to each administration of bevacizumab until it has decreased to ≤ 1 g/24-hour.
- Repeat 24-hour urine protein > 2 g: Bevacizumab dose should be withheld until 24-hour protein has decreased to ≤ 2 g. 24-hour protein should be further monitored prior to each administration of bevacizumab until it has decreased to ≤ 1 g/24-hour.

▪ Second and subsequent occurrence of ≥ 2+ proteinuria (dipstick):

  o 2+ (dipstick): Administer bevacizumab as scheduled, NO additional evaluation is required.

  o 3+ and 4+ proteinuria (dipstick): Administer bevacizumab as scheduled and collect 24-hour urine to determine the total protein within 3 days prior to the next scheduled dose is required:

    * 24-hour proteinuria ≤ 2 g: Administer bevacizumab as scheduled.

    * 24-hour proteinuria > 2 g: Bevacizumab treatment should be withheld pending next 24 hour total protein.

      - Repeat 24-hour urine protein ≤ 2 g: Administer bevacizumab as schedule. 24-hour protein should be further monitored prior to each administration of bevacizumab until it has decreased to ≤ 1 g/24-hour.
      - Repeat 24-hour urine protein > 2 g: Bevacizumab dose should be withheld until 24-hour protein has decreased to ≤ 2 g. 24-hour protein should be further monitored prior to each administration of bevacizumab until it has decreased to ≤ 1 g/24-hour.

▪ *Nephrotic syndrome* (Grade 4, CTCAE v 3.0): Discontinue bevacizumab treatment

Algorithm of Proteinuria (dipstick) and Bevacizumab Dose Interruption – please see Appendix 11.

7.3.2.6 Gastro-intestinal Perforations

All toxicity will be graded according to CTCAE v 3.0 guidelines. If a gastro-intestinal perforation occurs, appropriate treatment should be instituted and bevacizumab treatment will be discontinued permanently.

7.3.2.7 Wound Healing Complications

Bevacizumab therapy should not be initiated earlier than 28 days following major surgery or until the surgical wound is fully healed. In patients who experience wound healing

complications during bevacizumab treatment, bevacizumab should be withheld until the wound is fully healed. Bevacizumab therapy should be withheld for elective surgery.

7.3.2.8 Fistula or intra-abdominal abscess

Patients who develop a fistula or intra-abdominal abscess should discontinue bevacizumab. However, it is at investigator's discretion to continue after resolution of the findings in selected patients.

7.3.2.9 Infusion-related or allergic reactions

In the unlikely event of a suspected anaphylactic reaction during infusion of bevacizumab stop bevacizumab infusion and proceed as described in Appendix 12.

If other infusion-related (e.g., fever, chills, headache, nausea) or allergic reactions (e.g., fever, rash, urticaria, bronchospasm) occur, pre-medications should be given with the next dose, but the infusion time may not be reduced for the subsequent infusion. If the next dose is well tolerated with premedication, the subsequent infusion time may be reduced by 30 ± 10 min. as long as premedication continue to be used. If infusion-related AEs occur with the 60-min. infusion, all subsequent doses should be given over 90 ± 15 min. (with premedication). Similarly, if infusion-related AEs occur with the 30-min. infusion, all subsequent doses should be given over 60 ± 10 min. (with premedication).

For patients with grade 3 reactions, the bevacizumab infusion should be stopped and not restarted on that day. At the physician's discretion, bevacizumab may be permanently discontinued or re-instituted with pre-medications and at a rate of 90 ± 15 minutes. If the reaction occurred at the 90-minute rate, initially challenge at a slower infusion rate and gradually increase to 90 minutes. When bevacizumab is re-instituted, the patient should be monitored, per physician's usual practice, for a duration comparable to duration of reaction.

Note: If a hypersensitivity reaction occurs beyond the first administration, a serum sample has to be collected to allow for potential later investigation (for description of sampling please refer to HAHA sampling, i.e. Appendix 14).

7.3.2.10 Summary of Bevacizumab Schedule and Dosage Modification

The schedule of study drug administration will be modified in the event of certain grades of thrombotic, hemorrhagic, proteinuric, gastro-intestinal perforation, liver toxicity, wound healing complications, fistula or intra-abdominal abscess, hypertensive adverse events and infusion-related or allergic reactions, as summarized in Table 12.

Table 12 Bevacizumab Schedule Modification Due to Adverse Events

Event	Action to Be Taken
Thrombosis/Embolism	
Venous thromboembolic event – Grade 3 or incidentally discovered pulmonary embolus (first occurrence)	Hold bevacizumab for 2 weeks. Bevacizumab may be resumed after initiation of therapeutic-dose anticoagulant therapy as soon as all of the following criteria are met:<br>– The patient must be on a stable dose of anticoagulant and, if on an oral coumarin-derivative, have an INR within the target range (usually between 2 and 3) prior to restarting study drug treatment,<br>– The patient must not have had a Grade 3 or 4 haemorrhagic event since entering the study.<br>–
Arterial thromboembolic event – any grade or symptomatic Grade 4 venous thromboembolic event (first occurrence)	Permanently discontinue bevacizumab.
Hemorrhage	
Grade 1 and 2	• No schedule modification
Grade 3 or 4 (first occurrence)	• Discontinue bevacizumab.
Proteinuria (see Appendix 11)	
≤ 2 g protein/ 24 hr	• No schedule modification<br>• Repeat 24 hr urine collection until proteinuria improves to ≤ 1 g of protein/24 hr.
>2 g protein/ 24 hr	• Hold bevacizumab until proteinuria improves to ≤ 2 g of protein/24 hr.
Grade 4 proteinuria (nephrotic syndrome)	• Discontinue bevacizumab.
Gastro-intestinal perforation	
Gastro-intestinal perforation or dehiscence	Discontinue bevacizumab.
Wound healing complications	

Table 12 Bevacizumab Schedule Modification Due to Adverse Events (Cont.)

Prevention of wound healing complications	Bevacizumab therapy should not be initiated for at least 28 days following major surgery or until the surgical wound is fully healed. Bevacizumab therapy should be withheld for at least 28 days before elective surgery.
Wound healing complications	In patients who experience wound healing complications during bevacizumab treatment, bevacizumab should be withheld until the wound is fully healed.
Fistula or intra-abdominal abscess	
Fistula or intra-abdominal abscess	Patients who develop a fistula or intra-abdominal abscess should discontinue bevacizumab
Hypertension	
Grade 3	• Bevacizumab should be withheld for persistent or symptomatic hypertension<br>• Discontinue bevacizumab if hypertension is not controlled with medication
Grade 4	• Discontinue bevacizumab
Infusion-related or allergic reactions	
Grade 4	• In the event of a suspected anaphylactic reaction during infusion of bevacizumab stop bevacizumab infusion and proceed as described in Appendix 12.
Grade 3	• Stop bevacizumab infusion and do not restart on that day. At the physician's discretion, bevacizumab may be permanently discontinued or re-instituted with pre-medications.
[b]Liver toxicity: Applies only in cases when Grade 3 or 4 liver function test (ALAT, ASAT, ALP) increase is due to bevacizumab. If this is not the case, the guidances provided in Sections 7.3.5.3, 7.3.5.2 and Table 13 should be followed.	
Grade 3 or 4 first occurrence	• hold bevacizumab until toxicity has improved to Grade ≤ 1 and then resume treatment.
Grade 3 or 4 second occurrence	• discontinue bevacizumab

Note:

[a] A therapeutic dose of anticoagulant therapy is defined as a dose titrated to maintain an INR of at least 1.5 (usually within range of 2-3) for oral coumarin-derived anticoagulants or its equivalent for other anticoagulant medications administered to treat thromboembolic events. The oral coumarin-derived anticoagulant dose will be collected and INR will be assessed at baseline for all patients and throughout the treatment period in all patients receiving low dose or full dose oral coumarin-derived anticoagulants.

[b] Liver toxicity is not known with bevacizumab treatment.

7.3.3 Oxaliplatin Toxicities: General Considerations

7.3.3.1 Peripheral neuropathy

Oxaliplatin is consistently associated with two types of peripheral neuropathy: paresthesias and dysesthesias of the hands *and* feet *(chronic)*, and peri-oral region *(early onset)*. Patients treated with oxaliplatin in this study will be counseled to avoid cold drinks and exposure to cold water or air, especially for 3 to 5 days following oxaliplatin administration. For peripheral neuropathy, the scale in Table 15 (arms A and B) or Table 21 (arm C) will be used to determine dose adjustments.

7.3.3.2 Laryngopharyngeal dysesthesias

An unusual laryngopharyngeal dysesthesia, a sensation of loss of breathing (acute respiratory distress) without any objective evidence of respiratory distress (hypoxia, laryngospasm, or bronchospasm), also has been observed. This neurotoxicity may be induced or exacerbated upon exposure to cold.

If a patient develops laryngopharyngeal dysesthesia, the patient's oxygen saturation should be evaluated via a pulse oximeter and, if normal, reassurance, a benzodiazepine or other anxiolytic agent should be considered and the patient should be observed in the clinic until the episode has resolved. The oxaliplatin infusion may then be continued at 1/3 of the previous rate. Because this syndrome may be associated with the rapidity of oxaliplatin infusion, subsequent doses of oxaliplatin should be administered as 6-hour infusions (instead of the normal 2-hour infusion). Note for arms A & B: Administration of 5-FU must be postponed until the end of oxaliplatin infusion.

Patients on oxaliplatin should not receive cold drinks or ice chips on Day 1 of each cycle as this may exacerbate oral or throat dysesthesias, as well as laryngopharyngeal dysesthesia.

Administration of prophylactic medication such as Mg^{++}, Ca^{++} infusions or others is at the discretion of the investigator. However, their benefit has not been clearly established.

7.3.3.3 Allergic reactions

For Grade 1 or 2 acute hypersensitivity reactions, no dose modification of oxaliplatin is required if, in the investigator’s opinion, it is in the patient’s best interest to continue. Pre-medication with dexamethasone 20 mg IV, diphenhydramine 50 mg IV, and one of the following: cimetidine 300 mg IV, ranitidine 50 mg IV, or famotidine 20 mg IV 30 minutes prior to study drug administration is suggested. If an allergic reaction persists into the next cycle, administer 50 mg dexamethasone PO 12 hours and 6 hours prior to administration of oxaliplatin.

For Grade 3 or 4 acute hypersensitivity reactions, treatment with oxaliplatin should be discontinued.

7.3.3.4 Pulmonary fibrosis

In the case of respiratory symptoms indicative of pulmonary fibrosis such as nonproductive cough, dyspnea, crackles, rales, hypoxia, tachypnea or radiological pulmonary infiltrates, oxaliplatin should be interrupted pending further investigation. If interstitial pulmonary fibrosis is confirmed, permanently discontinue oxaliplatin.

7.3.3.5 Nausea/vomiting

The administration of 5-HT3 antagonists (granisetron, ondansetron or variants) with corticosteroids (e.g. dexamethasone) is recommended for prevention and treatment of oxaliplatin-induced emesis.

7.3.4 FOLFOX-4 & FOLFOX-4+Bev (arms A & B): Dose Modifications

For both treatment arms, if the next administration of chemotherapy requires an extended rest period for toxicity reason, bevacizumab should be held as well and will be resumed with the chemotherapy. This applies even if the toxicity is not related to bevacizumab.

Table 13 and Table 14 provide guidance for dose reductions for the first appearance of the specified toxicities excluding neurotoxicity (Table 15). At the second appearance of the toxicities, despite a prior dose reduction, the 5-FU dose could be further adjusted, if the investigator considers this to be in the best interest of the patient (otherwise the treatment should be discontinued). The second step dose adjustment of 5-FU bolus is to 200 mg/m^2/day, and of 5-FU infusion to 400 mg/m^2/day. This is not applicable to the second appearance of Grade 4 stomatitis or Grade 3/4 skin toxicity, in which case the treatment should be discontinued. Note: when reducing the dose of 5-FU, the dose of leucovorin should remain the same.

Hypertension and proteinuria are unlikely to be considered related to oxaliplatin plus 5-FU/LV. Guidance for dose modifications in these cases is provided in Sections 7.3.2.4, 7.3.2.5 and Table 12. If these adverse events would occur in the absence of bevacizumab (i.e. arm A or after bevacizumab has been discontinued in arm B due to adverse events), then guidance provided in section 7.3.4.3.3 should apply.

7.3.4.1 Hematological Toxicity in FOLFOX containing regimen

Table 13 Hematologic Toxicities: Oxaliplatin + 5-FU Dose Reductions for FOLFOX regimen

Toxicity	Grade	5-FU Bolus	5-FU Infusion	Oxaliplatin
Hemoglobin	All	no dose reduction	no dose reduction	no dose reduction
Neutropenia	3	no dose reduction	no dose reduction	no dose reduction
Neutropenia	4	300 mg/m^2	500 mg/m^2	75 mg/m^2
Febrile neutropenia [a]	3 or 4	300 mg/m^2	500 mg/m^2	75 mg/m^2
Thrombocytopenia [b]	3 or 4	300 mg/m^2	500 mg/m^2	75 mg/m^2

[a] Febrile neutropenia Gr. 3=ANC<1.0x10^9/L with fever ≥38.5°C,
Febrile neutropenia Gr. 4 =ANC<1.0x10^9/L with fever ≥38.5°C and life threatening sepsis

[b] Dose reduction to be applied at the second occurrence of persisting Grade 2 hematological toxicity leading to an extended rest period of ≥ 2 weeks. For details refer to Section 7.3.6.

The dose of leucovorin should remain the same.

Based on the most severe toxicity experienced since the last treatment, the following dose modifications should be used for non-hematological toxicities (see Table 14). The rest period should be extended until all non-hematological toxicities have subsided to Grade 1 or less, except increased bilirubin and ALAT which must recover to Grade 1 or baseline Grade, whichever is higher.

7.3.4.2 Non-Hematological Toxicity in FOLFOX containing regimen

Nausea and vomiting

For Grade 3 nausea and/or vomiting that occurs with suboptimal antiemetic therapy, treatment should be continued for the next course with an effective anti-emetic treatment and without dose modification.

Diarrhea

If Grade 3 or 4 diarrhea occurs at any time, the doses should be reduced according to Table 14. After Grade 3 or 4 diarrhea, the patient must have recovered to Grade 1 or less before treatment can be re-initiated.

Stomatitis

After Grade 3 or 4 stomatitis, the doses should be reduced according to Table 14. The patient must have recovered to Grade 1 or less before treatment can be re-initiated.

Cardiac toxicity

For Grade ≥ 2 cardiac toxicity which is attributable to 5-FU, patients will be permanently discontinued from 5-FU/LV therapy (Table 14).

Gastro-intestinal ulceration and bleeding

For Grade ≥ 2 gastro-intestinal toxicity which is attributable to 5-FU, treatment will be held until recovery to Grade ≤ 1.

Skin toxicity

Treatment will be held for Grade 3 or 4 toxicity until recovery to Grade ≤ 1 (Table 14).

Treatment may be withheld to allow for recovery. The extended rest period should not exceed 3 weeks from the scheduled administration. If the patient does not recover to Grade ≤ 1 in this timeframe, he/she will be taken off treatment.

Table 14 Non-Hematological Toxicities: Oxaliplatin + 5-FU/LV Dose Reductions for FOLFOX regimen

Toxicity	Grade	5-FU Bolus	5-FU Infusion	Oxaliplatin
* Allergic reactions	3 or 4	No dose reduction	No dose reduction	Stop treatment permanently
* Respiratory symptoms indicative of pulmonary fibrosis	any	No dose reduction	No dose reduction	Interrupt treatment and investigate cause of symptoms
* Interstitial pulmonary fibrosis not present at baseline	any	No dose reduction	No dose reduction	Stop treatment permanently
Nausea and/or vomiting despite premedication with an effective antiemetic therapy	3	No dose reduction	No dose reduction	No dose reduction
Nausea and/or Vomiting	4	300 mg/m^2	500 mg/m^2	75 mg/m^2
Diarrhea	3 or 4	300 mg/m^2	500 mg/m^2	75 mg/m^2
Stomatitis	3	300 mg/m^2	500 mg/m^2	no dose reduction
	4	200 mg/m^2	400 mg/m^2	75 mg/m^2
Cardiac toxicity (attributed to 5-FU)	≥2	Stop treatment permanently	Stop treatment permanently	Stop treatment permanently
Skin toxicity (extended rest period until recovery to Grade ≤1) (see 7.3.4.2.6)	3 or 4	200 mg/m^2	300 mg/m^2	no dose reduction
Note: the dose of leucovorin should remain the same.				

7.3.4.3 *Neurologic toxicity*

In case of neurological toxicity dose reductions should be done according to Table 15.

Table 15 Neurologic Toxicity Scale: Oxaliplatin Dose Adjustments for FOLFOX regimen

Toxicity	Grade	Duration of Toxicity		
		1 - 7 Days	>7 Days	Persistent between cycles [a]
Paresthesias/dysesthesias[b] that do not interfere with function	1	No dose reduction	No dose reduction	No dose reduction
Paresthesias/dysesthesias[b], interfering with function, but not activities of daily living (ADL)	2	No dose reduction	No dose reduction	75 mg/m^2
Paresthesias/dysesthesias[b] with pain or with functional impairment that also interfere with ADL	3	No dose reduction	75 mg/m^2	Stop treatment permanently
Persistent paresthesias/dysesthesias that are disabling or life-threatening	4	Stop treatment permanently	Stop treatment permanently	Stop treatment permanently
ACUTE: (during or after the 2 hour infusion) laryngopharyngeal dysesthesias[b]		Increase duration of next infusion to 6 hours[c]	N/A	N/A

a Not resolved by the beginning of the next cycle.
b May be cold-induced.
c May also be pre-treated with benzodiazepines.

7.3.4.4 *Other Toxicities or Miscellaneous Events*

Other toxicities or miscellaneous events not listed above (which could include i.e., auditory, ocular, metabolic, hepatic, central nervous system, renal, or pulmonary AEs) should be managed symptomatically if possible, if these are Grade < 3 AEs. For Grade ≥ 3 AEs the extended rest period to allow for recovery from toxicity to Grade 1 or less until readministration of chemotherapy should not exceed 3 additional weeks from the schedule. If medically appropriate, treatment can be reinstituted at a lower dose. After recovery from toxicity Grade 3 to Grade 2 or less, a dose reduction of all drugs in subsequent cycles should be performed as follows: oxaliplatin to 65 mg/m^2, 5-FU bolus to 300 mg/m^2 and 5-FU infusion to 500 mg/m^2. In case of no resolution to Grade 1 or less after a maximum of 3 weeks from the planned date of next cycle, the patient should be discontinued from treatment. In case of Grade 4 toxicity and otherwise not specified

above, the patient will be removed from treatment permanently and followed until resolution of toxicity according to the protocol.

7.3.5 XELOX+Bev (arm C): Dose Modifications

If the next administration of chemotherapy requires an extended rest period for toxicity reason, bevacizumab should be held as well and will be resumed with the chemotherapy. This applies even if the toxicity is not related to bevacizumab.

Hypertension and proteinuria are unlikely to be considered related to oxaliplatin plus capecitabine. Guidance for bevacizumab dose modifications in these cases is provided in Sections 7.3.2.4, 7.3.2.5 and Table 12. If these adverse events would occur in the absence of bevacizumab (after bevacizumab has been discontinued due to adverse events), then guidance provided in 7.3.5.2 and 7.3.5.3 should apply.

7.3.5.1 Dose Modifications for Hematologic Toxicity

In the XELOX regimen capecitabine and oxaliplatin will be modified simultaneously according to guidance in Table 16, Table 17, and Table 18.

Capecitabine is not expected to worsen or unduly prolong episodes of neutropenia/ granulocytopenia. Therefore, in case of unscheduled laboratory assessments during a treatment cycle showing Grade ≤ 2 hematologic toxicity capecitabine will be continued. Furthermore, administration of capecitabine should be interrupted during a treatment cycle if Grade 3 or 4 hematologic toxicity develops. The next treatment cycle can only start if hematologic toxicity has recovered to Grade ≤ 1. No dose reductions or interruptions will be required for anemia (non-hemolytic) as it can be satisfactorily managed by transfusions.

Table 16 Febrile Neutropenia (occurring anytime during treatment): Dose Modifications for XELOX regimen

	**Grade 3**<br>*ANC < 1.0x10^9/L with fever ≥ 38.5°C*	**Grade 4**<br>*ANC < 1.0x10^9/L with fever ≥ 38.5°C and life threatening sepsis*
1st occurrence	Capecitabine 75% of original dose + oxaliplatin 85 mg/m^2	Stop treatment permanently unless it is in the best interest of the patient to treat with capecitabine at 50% of original dose + oxaliplatin 85 mg/m^2
2nd occurrence	Stop treatment permanently unless it is in the best interest of the patient to treat with capecitabine at 50% of original dose + oxaliplatin 85 mg/m^2	Stop treatment permanently
Treatment *(including bevacizumab)* cannot start unless toxicity (except anemia) is resolved to Grade ≤ 1 (e.g. ANC ≥ 1.5 x10^9/L, Platelets ≥ 75 x10^9/L), see Section 7.3.*6*.		

Table 17 Neutropenia: Dose Modifications for XELOX regimen

	Grade 2<br>*$1.0 \leq ANC < 1.5 \times 10^9/L$*	Grade 3<br>*$0.5 \leq ANC < 1.0 \times 10^9/L$*	Grade 4<br>*$ANC < 0.5 \times 10^9/L$*
Laboratory value at start of a treatment cycle: extend rest period *(including bevacizumab)* until ANC ≥ 1.5 $\times 10^9$/L, platelets ≥ 75 $\times 10^9$/L, and recovery from non-haematologic toxicity to baseline or Grade ≤ 1 (see Section 7.3.5), then start treatment with doses indicated below.			
1st occurrence	No dose adjustment	Capecitabine 75% of original dose + oxaliplatin 100 mg/m^2	Capecitabine 50% of original dose + oxaliplatin 85 mg/m^2
2nd occurrence	No dose adjustment	capecitabine 75% of original dose + oxaliplatin 85 mg/m^2	stop treatment permanently
3rd occurrence	No dose adjustment	Stop treatment permanently unless it is in the best interest of the patient to treat with capecitabine monotherapy at 75% of original dose	Not applicable

Table 18 Thrombocytopenia and Anemia: Dose Modifications for XELOX regimen

**Thrombocytopenia**	***Platelets***<br>*$\geq 50 - < 75 \times 10^9/L$*	***Platelets***<br>*$\geq 25 - < 50 \times 10^9/L$*	***Platelets***<br>*$< 25 \times 10^9/L$*
Laboratory value at start of a treatment cycle: extend rest period *(including bevacizumab)* until ANC ≥ 1.5 $\times 10^9$/L, platelets ≥ 75 $\times 10^9$/L, and recovery from non-haematologic toxicity to baseline or Grade ≤ 1 (see Section 7.3.*6*), then start treatment with doses indicated below.			
1st occurrence [a]	No dose adjustment	Capecitabine 75% of original dose + oxaliplatin 100 mg/m^2	Capecitabine 50% of original dose + oxaliplatin 85 mg/m^2
2nd occurrence	No dose adjustment	Capecitabine 75% of original dose + oxaliplatin 85 mg/m^2	Stop treatment permanently unless it is in the best interest of the patient to treat with capecitabine monotherapy at 50% of original dose
3rd occurrence	No dose adjustment	Capecitabine 50% of original dose + oxaliplatin 85 mg/m^2	Stop treatment permanently
**Anemia (non-hemolytic) anytime during treatment**	**Hemoglobin 8.0 - < 10.0 g/dL**	**Hemoglobin 6.5 - < 8.0 g/dL**	**Hemoglobin < 6.5 g/dL**
any occurrence	No dose adjustment (can be managed by transfusion)	No dose adjustment (can be managed by transfusion)	No dose adjustment (can be managed by transfusion)
Treatment cannot start unless toxicity (except anemia) is resolved to Grade ≤ 1 (e.g. ANC ≥ 1.5 $\times 10^9$/L, Platelets ≥ 75 $\times 10^9$/L), see Section 7.3.6.<br>[a] Dose reduction to be applied at the second occurrence of persisting Grade 2 hematological toxicity leading to an extended rest period of ≥ 2 weeks. For details refer to Section 7.3.6.			

7.3.5.2 Capecitabine: Dose Modifications for Non-hematological Toxicity

If Grade 2, 3 or 4 non-hematological toxicity occurs, INTERRUPT CAPECITABINE IMMEDIATELY (see also Table 19) (except for toxicity solely related to oxaliplatin) then follow instructions below for further actions. Also refer to Section Capecitabine Precautions (7.5.2).

The recommendations found in this section for dose adjustments for capecitabine should be followed for those toxicities usually considered to be related to capecitabine treatment. Thus, neurosensory toxicities do not result in a dose reduction for capecitabine. Instead, one should follow the instructions for dose adjustments of oxaliplatin found in Table 21. Hypertension and proteinuria are unlikely to be considered related to oxaliplatin plus capecitabine. For dose modification in these cases reference should first be made to guidance in Sections 7.3.2.4, 7.3.2.5 and Table 12.

If these adverse events would occur in the absence of bevacizumab (after bevacizumab has been discontinued due to adverse events), then guidance provided in 7.3.5.2 and Table 19 should apply.

If the calculated creatinine clearance decreases during treatment to a value <30 mL/min, treatment should be discontinued.

The specific dose reductions (i.e. the number of tablets to be taken at various dose levels) can be found in Appendix 8. Once the dose has been reduced it should not be increased at a later time, except if oxaliplatin is permanently discontinued (see Section 7.3.7).

Hyperbilirubinemia will be managed as outlined in Section 7.5.2: Administration of capecitabine should be interrupted if treatment-related elevations in bilirubin of >3.0 x ULN (Grade 3) occur. Treatment may be resumed when bilirubin decreases to ≤3.0 x ULN (Grade 2 or less).

For treatment-related elevations in hepatic aminotransferases (ALT, AST) and alkaline phosphatase (ALP) the guidance in [Table 19] is consistent with the management outlined in Section 7.5.2.

Note: capecitabine treatment interruptions are regarded as lost treatment days and the planned treatment schedule should be maintained. Missed doses due to treatment interruptions must not be replaced (see Appendix 9).

Table 19 Non-hematologic AEs: Dose Adjustments for Capecitabine

Note: Treatment must be interrupted for toxicities Grade > 2 and cannot continue unless toxicities resolves to Grade < 1.

	Grade 2	Grade 3	Grade 4
1st occurrence	no dose reduction; prophylaxis where possible	75% of original dose with prophylaxis where possible	Discontinue treatment permanently- unless investigator considers it to be in the best interest of the patient to continue at 50% of original dose
2nd occurrence	75% of original dose	50% of original dose	
3rd occurrence	50% of original dose	Stop treatment permanently- unless it is considered by the investigator to be in the best interest of the patient to stay on treatment	
4th occurrence	Stop treatment permanently - unless it is considered by the investigator to be in the best interest of the patient to stay on treatment		

Grade ≥ 2 Diarrhea

Capecitabine can induce diarrhea, which can sometimes be severe. Patients with severe diarrhea should be carefully monitored and, if they become dehydrated, should be given fluid and electrolyte replacement. If Grade 2, 3 or 4 diarrhea occurs, administration of capecitabine should be immediately interrupted until the diarrhea resolves or decreases in intensity to Grade ≤ 1. Following the second occurrence of Grade 2 or higher toxicity, subsequent doses of capecitabine should be decreased (see Table 19). Standard anti-diarrhea treatments (e.g. loperamide) should be initiated, as medically appropriate, as early as possible. Capecitabine can not be re-started until diarrhea has resolved to Grade 0 or 1, and no loperamide has been given for 24 hours.

Grade ≥ 2 Nausea/Vomiting

Capecitabine can induce nausea or vomiting. If Grade 2, 3 or 4 nausea and/or vomiting occurs, administration of capecitabine should be immediately interrupted until these symptoms resolve or decrease in intensity to Grade ≤ 1. Treat symptomatically. For prophylaxis, the patients must be supplied with oral anti-emetics in order to treat themselves in case nausea or vomiting occurs at home. The administration of oral metoclopramide is recommended for capecitabine-induced nausea (the use of 5-HT3 antagonists is at the discretion of the investigator). Adequate secondary therapeutic and prophylactic treatment has to be initiated once nausea or vomiting has occurred. If nausea/vomiting recurs despite adequate prophylaxis, then dose modifications should also be made according to Table 19.

Grade ≥ 2 Hand/Foot Syndrome

Hand-and-foot syndrome (palmar-plantar erythrodysesthesia or chemotherapy-induced acral erythema) is a cutaneous toxicity with a severity range of Grades 1 to 3 as shown:

Grade 1: skin changes or dermatitis without pain (e.g. erythema, peeling).

Grade 2: skin changes with pain, not interfering with function.

Grade 3: skin changes with pain, interfering with function.

If Grade 2 or 3 hand-foot syndrome occurs, administration of capecitabine should be immediately interrupted until the event resolves or decreases in intensity to Grade ≤ 1. Subsequent doses of capecitabine should be administered as per [Table 19].

Hand-foot syndrome should be treated symptomatically (i.e. use of emollients is recommended). The use of vitamin B_6 is not permitted for symptomatic or secondary prophylactic treatment of hand-foot syndrome; impaired efficacy has been reported with concomitant use of vitamin B_6 and cisplatin [55].

Grade ≥ 2 Stomatitis

If Grade 2 or 3 stomatitis occurs, administration of capecitabine should be immediately interrupted until the event resolves or decreases in intensity to Grade ≤ 1. Treat symptomatically. Subsequent doses of capecitabine should be administered as per Table 19.

Cardiac toxicity

For Grade ≥ 2 cardiac toxicity which is attributable to capecitabine, patients will be permanently discontinued from capecitabine therapy.

7.3.5.3 Oxaliplatin: Dose Modifications for Non-hematological Toxicity

For toxicities or miscellaneous events (including, but not limited to auditory, ocular, metabolic, hepatic, central nervous system, renal, or pulmonary AEs) not listed below in Table 20 and Table 21, management should be symptomatic, if possible, if these are Grade < 3 AEs. For Grade ≥ 3 AEs, extended rest period to allow for recovery from toxicity to Grade 1 or less until readministration of chemotherapy should not exceed 3 weeks from the schedule. Then oxaliplatin treatment should be reinstituted at a lower dose, if medically appropriate. After recovery from toxicity Grade 3 to Grade 2 or less, a dose reduction of oxaliplatin to 100 mg/m^2 in subsequent cycles should be made. In case of no resolution to Grade 2 or less after a maximum of 3 weeks from the planned date of next cycle, the patient should be permanently discontinued from oxaliplatin treatment. In case of Grade 4, the patient will be removed from oxaliplatin treatment permanently and followed until resolution of toxicity according to the protocol. If the investigator considers it to be in the best interest of the patient to continue capecitabine and/or bevacizumab after resolution of Grade 4 toxicity, it must be discussed with and approved by the Roche study team.

Hypertension and proteinuria are unlikely to be considered related to oxaliplatin. For dose modification in these cases reference should first be made to guidance in Sections 7.3.2.4, 7.3.2.5 and Table 12.

If these adverse events would occur in the absence of bevacizumab (after bevacizumab has been discontinued due to adverse events), then guidance provided in section 7.3.5.3 should apply.

Table 20 Oxaliplatin Dose Adjustments for Non- hematologic AEs (Arm C)

Toxicity	Grade	Dose Adjustment
* Allergic reactions	3 or 4	Stop treatment permanently
* Respiratory symptoms indicative of pulmonary fibrosis	any	Interrupt treatment and investigate cause of symptoms
* Interstitial pulmonary fibrosis not present at baseline	any	Stop treatment permanently
Nausea and/or vomiting despite premedication with an effective antiemetic therapy	3	100 mg/m^2
Nausea and/or Vomiting	4	100 mg/m^2
Diarrhea	3 or 4	100 mg/m^2
Stomatitis	3	no dose reduction
	4	100 mg/m^2
Skin toxicity (extend rest period until recovery to Grade ≤1)	3 or 4	no dose reduction
* No dose adjustment for capecitabine (if in the best interest of the patient).		

Table 21 Neurologic Toxicity Scale for Oxaliplatin Dose Adjustments (Arm C)

Toxicity	Grade	Duration of Toxicity		
		1 - 7 Days	>7 Days	Persistent between cycles [a]
Paresthesias/dysesthesias[b] that do not interfere with function	1	No dose reduction	No dose reduction	No dose reduction
Paresthesias/dysesthesias[b], interfering with function, but not activities of daily living (ADL)	2	No dose reduction	No dose reduction	100 mg/m^2
Paresthesias/dysesthesias[b] with pain or with functional impairment that also interfere with ADL	3	No dose reduction	100 mg/m^2	Stop treatment permanently
Persistent paresthesias/dysesthesias that are disabling or life-threatening	4	Stop treatment permanently	Stop treatment permanently	Stop treatment permanently
ACUTE: (during or after the 2 hour infusion) laryngopharyngeal dysesthesias[b]		Increase duration of next infusion to 6 hours[c]	N/A	N/A

a Not resolved by the beginning of the next cycle.
b May be cold-induced.
c May also be pre-treated with benzodiazepines.

7.3.6 Treatment schedule modifications

Extended rest periods are required until (Appendix 9):

- Neutrophil count is ≥ 1.5x 10^9/L and platelet count is ≥ 75x 10^9/L.

  According to clinical practice, sustained recovery from hematological toxicity may be observed requiring an extended rest period. In case that criteria for a dose reduction after the last administration of FOLFOX-4 or XELOX were not fulfilled according to the criteria mentioned in Table 13 or 7.3.5.1 a dose reduction has to be performed in order to avoid increasing extended rest periods during weeks 1 to 24. Thus, at the second occurrence of persisting Grade 2 hematological toxicity leading to an extended rest period of ≥ 2 weeks please refer to the dose reduction described for Grade 3/4 thrombocytopenia observed during treatment with FOLFOX-4 (Table 13, footnote b) or Grade 3 thrombocytopenia for XELOX (Table 18, 1st occurrence, footnote a), respectively.

– Recovery from any treatment-related non-hematological toxicity (except e.g. alopecia) to baseline grade or Grade ≤1

– Prolonged toxicity: if toxicity requires an extend rest period of all study drugs exceeding three weeks, the patient will be withdrawn from the study for toxicity.

7.3.6.1 Bevacizumab-related specific treatment modifications

Administration of bevacizumab will be modified in the event of certain grades of thrombosis/embolism, hemorrhage, proteinuria, gastro-intestinal perforation, wound healing complications, fistula or intra-abdominal abscess, hypertensive adverse events, infusion-related or allergic reactions and liver toxicity, believed to be associated with bevacizumab, as summarized in Table 12. Provided that these are the only bevacizumab - related events and that there are no reasons to modify capecitabine, 5-FU/LV or oxaliplatin, patients may continue to receive capecitabine, 5-FU/LV or oxaliplatin on the usual schedule and resume bevacizumab at the next protocol-specified scheduled visit.

In order to continue with the treatment with bevacizumab after an interruption of more than

- 3 consecutive administrations of bevacizumab in arm B during part 1 of the study treatment phase (week 1-24)
- 2 consecutive administrations of bevacizumab in arm B during part 2 of study treatment phase (week 25-48)
- 2 consecutive administrations of bevacizumab in arm C.

– the investigator has to provide a rational to the medical monitor (i.e. reason for extended rest and patient's interest to resume treatment) before approval of continuation.

7.3.6.2 Oxaliplatin-related specific treatment modifications:

Presence of paresthesias with pain or with persistent functional impairment [see Table 15 (arms A & B) or Table 21 (arm C)] as the only toxicity at the time of the next planned administration of oxaliplatin will result in an interruption of oxaliplatin treatment only, but continuation of capecitabine or 5-FU/LV and bevacizumab. If the neurological toxicity is still present at the time of the next planned treatment cycle, oxaliplatin will be discontinued permanently. Capecitabine or 5-FU/LV with bevacizumab will then be continued at the discretion of the investigator.

7.3.7 Dose Escalations for Capecitabine or 5-FU/LV after Discontinuation of Oxaliplatin

If capecitabine or 5-FU/LV with bevacizumab is continued after discontinuation of oxaliplatin and in the absence of disease recurrence/new occurrence, capecitabine or 5-FU/LV should be continued without dose escalation for at least one more cycle. Thereafter, in the absence of Grade 2, 3 or 4 toxicities, capecitabine or 5-FU doses that

have been previously reduced can subsequently be escalated once per cycle in a stepwise manner.

– For capecitabine: 50% of the baseline dose to 75% of the baseline dose, then 75% of the baseline dose to 100% of the baseline dose. Specific doses of capecitabine can be determined by referring to the dosing tables in Table 7.

– For 5-FU: bolus 200mg/m^2 to 300mg/m^2, then 300mg/m^2 to 400mg/m^2; infusion 300mg/m^2 or 400mg/m^2 to 500mg/m^2, then 500mg/m^2 to 600mg/m^2. (Leucovorin dose should remain the same.)

Doses of capecitabine or 5-FU should not be escalated beyond 100% of the baseline dose.

7.4 Criteria for Premature Withdrawal from Study Treatment

Patients have the right to withdraw from the study at any time for any reason. The investigator also has the right to withdraw patients from the study treatment in the event of intercurrent illness, adverse events and treatment failure including failure after a prescribed procedure, protocol violations, administrative reasons or other reasons.

An excessive rate of withdrawals can render the study uninterpretable; therefore, unnecessary withdrawal of patients should be avoided. Should a patient decide to withdraw, all efforts will be made to complete and report the observations as thoroughly as possible.

The investigator should contact the patient either by telephone or through a personal visit, or a responsible relative must be contacted to determine as completely as possible the reason for the withdrawal. A complete final evaluation at the time of the patient's withdrawal should be made with an explanation of why the patient is withdrawing from the study. If the reason for removal of a patient from study treatment is an adverse event or an abnormal laboratory test result, the principal specific event or test will be recorded on the CRF. After withdrawal, patients will be followed for survival as outlined in Table 11 unless they have withdrawn consent. For scheduled follow up assessments please refer to 5.2.4.1.

7.5 Warnings and Precautions

Patients receiving therapy with bevacizumab, capecitabine, oxaliplatin, and 5-FU/LV should be monitored by a physician experienced in the use of cancer chemotherapy agents. Use of these agents in pregnant or breast-feeding women is contraindicated.

7.5.1 Bevacizumab Precautions

Hypertension

(see also Section 7.3.2.4):

An increased incidence of hypertension was observed in patients treated with bevacizumab. Hypertension was generally treated with oral anti-hypertensives such as angiotensin-converting enzyme inhibitors, diuretics and calcium-channel blockers. It rarely resulted in discontinuation (0.7% of all patients treated with bevacizumab) or

hospitalisation, and resulted in hypertensive encephalopathy in one case (0.1%). The risk of bevacizumab associated hypertension did not correlate with the patients' baseline characteristics, underlying disease or concomitant therapy.

In the phase III, randomised, double-blind, active-controlled study in metastatic carcinoma of the colon or rectum (AVF2107g), hypertension of any grade (NCI-CTC, version 2.0) occurred in 22.4% of patients receiving IFL (Irinotecan/5-FU/LV) + bevacizumab compared with 8.3% of patients receiving IFL alone. Grade 3 hypertension (requiring oral anti-hypertensive medication) was reported in 11.0% of patients receiving IFL+ bevacizumab compared with 2.3% of patients receiving IFL alone. At week 24 of treatment, the mean change of blood pressure (BP) from baseline was diastolic BP +4.1 mmHg and systolic BP +5.5 mmHg in patients treated with bevacizumab.

In Study AVF2192g, hypertension of any grade occurred in 32.0% of patients treated with 5-FU/LV plus bevacizumab (Arm 2) compared to 4.8% of patients treated with 5-FU/LV plus placebo (Arm 1). Grade 3 hypertension was observed in 16.0% of patients in Arm 2 compared to 2.9% of patients in Arm 1. At week 24 of treatment, the mean change of BP from baseline was diastolic BP +5.4 mmHg and systolic +8.4 mmHg in bevacizumab-treated patients. Hypertension did not lead to death or study drug discontinuation in this study. No hypertensive crisis (Grade 4) was reported.

There is no information on the effect of bevacizumab in patients with uncontrolled hypertension at the time of initiating bevacizumab therapy. Therefore, caution should be exercised before initiated bevacizumab therapy in these patients. Monitoring of blood pressure is recommended during bevacizumab therapy.

Proteinuria

(see also Section 7.3.2.5 and Appendix 11*):*

In study AVF2107g, proteinuria was reported as an adverse event in 21.7% of patients receiving IFL alone and 26.5% of patients receiving IFL + bevacizumab. There was no Grade 4 (NCI-CTC, version 2.0) proteinuria, and incidences of Grade 2 and 3 proteinuria were similar in both arms.

Proteinuria, reported as adverse event, was observed in 23.3% of all patients treated with bevacizumab. It ranged in severity from clinically asymptomatic, transient, trace proteinuria to nephrotic syndrome, with the great majority as Grade 1 proteinuria. The proteinuria seen in bevacizumab clinical trials was not associated with renal dysfunction and rarely required permanent discontinuation of bevacizumab therapy.

In Study AVF2192g, proteinuria was reported as adverse event in 38.0% of patients receiving 5-FU/LV plus bevacizumab (Arm 2) and 19.2% of patients receiving 5-FU/LV plus placebo (Arm 1). The majority of these events was Grade 1 (30.0% vs. 15.4%). There was no Grade 4 proteinuria (nephrotic syndrome) and only one case of Grade 3 proteinuria was reported in Arm 2. No proteinuria resulted in death or study drug discontinuation.

Patients with a history of hypertension may be at increased risk for the development of proteinuria when treated with bevacizumab. There is evidence suggesting that Grade 1

proteinuria may be related to bevacizumab dose. Monitoring of proteinuria by dipstick urinalysis is recommended prior to starting and during bevacizumab therapy. Bevacizumab should be discontinued in patients who develop CTCAE (version 3.0) Grade 4 proteinuria (nephrotic syndrome).

Gastrointestinal Perforation

Bevacizumab has been associated with serious cases of gastrointestinal perforation in patients with metastatic carcinoma of the colon or rectum. In study AVF2107g in patients with metastatic colorectal cancer, there were six reports of gastrointestinal perforation in the IFL plus bevacizumab arm and one report in the 5-FU/LV plus bevacizumab arm compared with none events in the IFL plus placebo arm. In two patients this event had a fatal outcome; the remaining five recovered but three patients resumed bevacizumab therapy. The presentation of these events varied in type and severity, ranging from free air seen only on the plain abdominal X-ray, which resolved without treatment, to a colonic perforation with abdominal abscess and fatal outcome. The common feature among these cases was intra-abdominal inflammation, either from gastric ulcer disease, tumour necrosis, diverticulitis or chemotherapy-associated colitis. Nevertheless, a causal association of an intra-abdominal inflammatory process and gastrointestinal perforation to treatment with bevacizumab has not been established. However, caution should be exercised when treating patients with intra-abdominal inflammatory process with bevacizumab.

In study AVF2192g, two cases of gastrointestinal perforation were observed in patients metastatic colorectal cancer treated with 5-FU/LV plus bevacizumab arm compared to none in 5-FU/LV plus placebo arm. One case had fatal outcome whereas the other resolved but study treatment was discontinued due to the event. In both cases, perforation occurred at the site of sigmoid colon diverticulum.

Patients with metastatic carcinoma of the colon or rectum may be at increased risk for the development of gastrointestinal perforation when treated with bevacizumab and chemotherapy.

No gastrointestinal perforation has been observed in any other bevacizumab clinical trials.

Wound Healing

As bevacizumab may adversely impact wound healing, patients who had major surgery within the last 28 days were excluded from participation in study AVF2107g. In this study, 173 patients in IFL + bevacizumab arm underwent cancer-related surgery between 28 and 60 days prior to starting bevacizumab therapy. There was no increased risk of post-operative bleeding or wound healing complications observed in these patients.

Forty patients in the IFL + bevacizumab arm underwent major surgery while receiving bevacizumab, of which 4 patients experienced an adverse event consistent with post-operative bleeding or wound healing complications. There were no similar complications observed in the 25 patients from the IFL + placebo arm who also underwent major surgery.

In Study AVF2192g, 39 patients in 5-FU/LV plus placebo arm (Arm 1) and 43 patients in 5-FU/LV plus bevacizumab arm (Arm 2) underwent cancer-related surgery between 28 and 60 days prior to starting study drug. No patients experienced Grade 3/4 wound healing and bleeding complications within 60 days after prior major surgery.

Fifteen patients in Arm 2 underwent major surgery while receiving bevacizumab, of which 3 experienced Grade 3/4 wound healing or bleeding complications within 60 days of surgery. Three patients in Arm 1 underwent major surgery during study treatment and none experienced Grade 3/4 wound healing or bleeding complications.

Thus, it is recommended that bevacizumab therapy not be initiated within 28 days following major surgery as such patients were excluded from clinical trials. Patients undergoing major surgery during bevacizumab therapy may be at increased risk for post-operative bleeding and/or wound healing complications during bevacizumab therapy. Therefore, caution should be exercised in these patients.

Haemorrhage

(see also section 7.3.2.2)

Overall, 4.0% of NCI-CTC (version 2.0) Grade 3 and 4 bleeding events were observed in all patients treated with bevacizumab. In Study AVF2107g, there was no significant difference in the incidence of Grade 3 and 4 bleeding events observed in IFL + bevacizumab arm (3.1%) and IFL + placebo arm (2.5%). A similar observation was noted in study AVF2192g; the overall incidence of Grade 3 and 4 bleeding events was 5.0% in 5-FU/LV plus bevacizumab arm (5.0%) and 2.9% in 5-FU/LV plus placebo arm.

The haemorrhagic events that have been observed in bevacizumab clinical studies were predominantly tumour-associated haemorrhage (see below) and minor mucocutaneous haemorrhage.

Tumour-associated haemorrhage was observed in phase I and phase II bevacizumab studies. Six serious events, of which 4 had fatal outcome, were observed in patients with non-small cell lung cancer receiving bevacizumab. These events occurred suddenly and presented as major or massive haemoptysis in patients with either squamous cell histology and/or tumours located in the centre of the chest in close proximity to major blood vessels. In five of these cases, these haemorrhages were preceded by cavitation and/or necrosis of the tumour.

Tumour-associated haemorrhage was also seen rarely in other tumour types and locations, including central nervous system (CNS) bleeding in a patient with hepatoma with occult CNS metastases and continuous oozing of blood from a thigh sarcoma with necrosis.

In Study AVF2107g, five haemorrhagic events in IFL + bevacizumab arm (three rectal haemorrhages, one gastrointestinal haemorrhage and one melaena) were assessed as tumour-associated haemorrhages. The addition of bevacizumab did not result in significant increase in the incidence or severity of Grade 3 or 4 haemorrhagic events in this study.

In study AVF2192g three patients in 5-FU/LV + Avastin arm (Arm 2) experienced Grade 3 and 4 gastrointestinal haemorrhages that were assessed as tumour-associated.

Across all bevacizumab clinical trials, *mucocutaneous haemorrhage* has been seen in 20% - 40% of patients treated with bevacizumab. These were most commonly Grade 1 epistaxis that lasted less than 5 minutes, resolved without medical intervention and did not require any changes in bevacizumab treatment regimen. In Study AVF2107g, epistaxis was reported in 35.3% of patients receiving IFL+bevacizumab compared with 10.2% of patients receiving IFL alone.

In study AVF2192g, epistaxis (all Grade 1) was observed in 22.0% of patients receiving 5-FU/LV + Avastin arm (Arm 2) compared to 16.3% of patients receiving 5-FU/LV + placebo (Arm 1).

There have also been less common events of minor mucocutaneous haemorrhage in other locations, such as gingival bleeding and vaginal bleeding.

The risk of CNS haemorrhage in patients with CNS metastases receiving bevacizumab could not be evaluated, as patients with history or evidence upon physical examination of central nervous system (CNS) metastases were excluded from all clinical trials. The use of bevacizumab is contraindicated in patients with untreated CNS metastases.

Patients with metastatic cancer of the colon or rectum might have an increased risk of tumour-associated haemorrhage.

There is no information on the safety profile of bevacizumab in patients with congenital bleeding diathesis, acquired coagulopathy or in patients receiving full dose of anticoagulants for the treatment of thromboembolism prior to starting bevacizumab treatment, as such patients were excluded from clinical trials. However, patients who developed venous thrombosis while receiving bevacizumab therapy did not appear to have increased rate of serious bleeding when treated with full dose of warfarin and bevacizumab concomitantly.

Thrombosis/Embolism

(see also section 7.3.2.3)

In Study AVF2107g, 16.2% of patients receiving IFL + placebo (Arm 1) and 19.4% of patients receiving IFL+bevacizumab (Arm 2) experienced thromboembolic events. In study AVF2192g, the overall incidence of thromboembolic events was 18.0% in 5-FU/LV plus bevacizumab arm (Arm 2) and 18.3% in 5-FU/LV + placebo arm (Arm 1).

Arterial Thromboembolism

In study AVF2107g, the incidence of arterial thromboembolic events (including CVAs, MIs, TIAs, and other arterial thromboembolic events) was higher in patients receiving IFL plus bevacizumab (3.3%) compared to patients receiving IFL plus placebo (1.3%). In study AVF2192g the incidence of arterial thromboembolic events was also reported to be higher in the 5-FU/LV plus bevacizumab arm (10.0%) compared to the 5FU/LV arm (4.8%).

In five randomised trials including AVF2107g and AVF 2192g (N=1745), arterial thromboembolic events including CVAs, MIs, TIAs, and other thromboembolic events occurred in 4.9% (49/1004) of patients treated with bevacizumab in combination with chemotherapy compared to 2.3% (17/741) of patients treated with chemotherapy alone. In patients treated with bevacizumab plus chemotherapy, arterial thromboembolic events led to a fatal outcome in 1.1% (11/1004). In patients treated with chemotherapy alone, a fatal outcome from arterial thromboembolic events was reported in 0.8% (6/741). CVAs (including TIAs) occurred in 2.2% of patients treated with bevacizumab in combination with chemotherapy and 0.5% of patients treated with chemotherapy alone. MI occurred in 2.2% of patients treated with bevacizumab in combination with chemotherapy compared to 1.3% of patients treated with chemotherapy alone.

Bevacizumab should be permanently discontinued in patients who develop arterial thromboembolic events.

A history of arterial thromboembolic events or age greater than 65 years was associated with an increased risk of arterial thromboembolic events during bevacizumab therapy. Patients receiving Avastin plus chemotherapy with a history of arterial thromboembolism and age greater than 65 years have a higher risk. Caution should be taken when treating these patients with bevacizumab.

Venous Thromboembolism

In Study AVF2107g, venous thromboembolic events, including deep venous thrombosis, pulmonary embolism and thrombophlebitis, occurred in 15.2% and 16.4% of patients in Arms 1 and 2, respectively. It could not be determined if these events were due to the patients' underlying cancer, their cytotoxic chemotherapy, bevacizumab or other risk factors.

In study AVF2192g, the incidence of venous thromboembolic events was lower in the 5-FU/LV plus bevacizumab arm compared to that in control (9.0% vs. 13.5%).

Congestive Heart Failure

In the phase III controlled clinical trial of metastatic breast cancer, there were 7 reports (3%) of congestive heart failure (CHF) in patients treated with bevacizumab compared with two (1%) seen in the controlled group. These events varied in severity from asymptomatic declines in left ventricular ejection fraction to symptomatic CHF requiring hospitalisation and treatment. All the patients treated with bevacizumab were previously treated with anthracyclines (doxorubicin cumulative dose range 240 – 360mg/m^2). Many of these patients also had prior radiotherapy to the left chest wall. Most of these patients showed improved symptoms and/or left ventricular function following appropriate medical therapy. There was no information on patients with pre-existing CHF of NYHA II – IV at the time of initiating bevacizumab therapy, as these patients were excluded from clinical trials.

Prior anthracyclines exposure and/or prior radiation to the chest wall may be possible risk factors for the development of CHF. Caution should be exercised before initiating bevacizumab therapy in patients with these risk factors.

In patients with metastatic cancer of the colon or rectum, there was no increased incidence of CHF in patients treated with bevacizumab.

Hypersensitivity reaction

The incidence of hypersensitivity reactions observed in patients treated with bevacizumab in clinical trials was similar to that in controls. In case a Grade 3/4 hypersensitivity reaction attributable to bevacizumab occurs, discontinue bevacizumab treatment and treat the patient according to guidance in Appendix 12. If a hypersensitivity reaction occurs beyond the first administration, a serum sample has to be collected to allow for potential later investigation (for description of sampling please refer to HAHA sampling, i.e. Appendix 14). In case of recovery a re-exposure under appropriate premedication should be attempted with a reduced infusion rate, if it is judged to be in the interest of the patient.

7.5.2 Capecitabine Precautions

Capecitabine is foreseen as an outpatient treatment, and in certain circumstances adverse events that could occur, such as diarrhea, can rapidly become serious. In the case where a patient experiences any toxicity in between scheduled visits, the patient should be encouraged to contact the clinic as soon as is practical, for further directions or for treatment. It is essential that the patients are informed to interrupt capecitabine treatment as soon as a Grade 2 toxicity occurs, therefore the patients will need specific explanations what to do in the case of the occurrence of the most frequent toxicities (diarrhea, hand-foot syndrome, and stomatitis).

Renal Impairment: Capecitabine is contraindicated in patients with severe renal impairment [creatinine clearance below 30 mL/min (Cockroft and Gault, see Appendix 6)]; treatment should not be started or continued in patients with severe renal impairment. Reduced clearance of 5'-DFUR has been reported in moderately renally impaired patients, which correlated with an increased incidence of Grade 3 or 4 adverse events in patients with moderate renal impairment (creatinine clearance 30-50 mL/min). There is no evidence of a direct nephrotoxic effect of capecitabine. However, patients with moderate renal impairment (creatinine clearance 30-50 mL/min) are not eligible for this study.

Coagulopathy: Patients receiving concomitant capecitabine and oral coumarin-derived anticoagulants should have their anticoagulant response (INR or prothrombin time) monitored frequently in order to adjust the anticoagulant dose accordingly. A clinically important capecitabine-warfarin drug interaction was demonstrated in a clinical pharmacology trial. Altered coagulation parameters and/or bleeding, including death, have been reported in patients taking capecitabine concomitantly with coumarin-derived anticoagulants such as warfarin and phenprocoumon. Postmarketing reports have shown clinically significant increases in prothrombin time (PT) and INR in patients who were stabilized on anticoagulants at the time capecitabine was introduced. These events occurred within several days and up to several months after initiating capecitabine therapy and, in a few cases, within one month after stopping capecitabine. These events occurred in patients with and without liver metastases. Age greater than 60 and a

diagnosis of cancer independently predispose patients to an increased risk of coagulopathy (see also [54, 81]).

Diarrhea: Capecitabine can induce diarrhea. Patients with severe diarrhea should be carefully monitored and given fluid and electrolyte replacement. If Grade 2, 3 or 4 diarrhea occurs, administration of capecitabine should be immediately interrupted until the diarrhea resolves or decreases in intensity to Grade 1.

Hand-Foot Syndrome: Capecitabine has been shown to cause hand-foot syndrome (palmar-plantar erythrodysesthesia or chemotherapy-induced acral erythema-erythema). If Grade 2 or 3 hand-foot syndrome occurs, administration of capecitabine should be interrupted until the event resolves or decreases in intensity to < Grade 1. Subsequent doses of capecitabine should be administered as per Table 19.

Cardiotoxicity: There has been cardiotoxicity associated with fluorinated pyrimidine therapy (including capecitabine) including myocardial infarction, angina, dysrhythmias, cardiogenic shock, sudden death and electrocardiograph changes. These adverse events may be more common in patients with a prior history of coronary artery disease.

Hepatic Insufficiency: In patients with mild to moderate hepatic dysfunction due to liver metastases, caution should be exercised when capecitabine is administered but no dose reduction is necessary. The effect of severe hepatic dysfunction on capecitabine is not known.

Hyperbilirubinemia: Administration of capecitabine should be interrupted if treatment-related elevations in bilirubin of >3.0 x ULN (Grade 3) or treatment-related elevations in hepatic aminotransferases (ALT, AST) of >2.5 x ULN occur. Treatment may be resumed when bilirubin decreases to ≤3.0 x ULN (Grade 2) or hepatic aminotransferases decrease to ≤2.5 x ULN.

Geriatric patients: Patients > 80 years old may experience a greater incidence of Grade 3 or 4 adverse events, in particular diarrhea, nausea, hand-foot syndrome and vomiting.

Pregnancy (see also Section 7.2.5): Female patients must be instructed to stop taking capecitabine if they become pregnant during the study, and immediately inform the investigator. The Investigator should counsel the patient, and discuss the risk of continuing with the pregnancy and the possible effects on the fetus. Monitoring of the patient should continue until conclusion of the pregnancy. The investigator should report all pregnancies in the study to the Sponsor.

Contraindications: Capecitabine is contraindicated in patients with known hypersensitivity to capecitabine 5-fluorouracil or to any of the excipients, in patients who have a history of severe and unexpected reactions to fluoropyrimidine therapy, and in patients with known DPD deficiency. Capecitabine is contraindicated in patients with severe leucopenia, neutropenia, or thrombocytopenia, severe hepatic impairment, or severe renal impairment (creatinine clearance below 30 ml/min). Use of capecitabine is contraindicated during pregnancy and lactation, and concomitantly with sorivudine or its chemically related analogues, such as brivudine.

7.5.3 Oxaliplatin Precautions

Anaphylactic/anaphylactoid reactions: As is the case for other platinum compounds, hypersensitivity and anaphylactic/anaphylactoid reactions have been reported. These allergic reactions were similar in nature and severity to those reported with other platinum-containing compounds, i.e., rash, urticaria, erythema, pruritis, and, rarely, bronchospasm and hypotension. These reactions occur within minutes of administration (can also occur at other than the first cycle of treatment) and should be managed with appropriate supportive therapy (e.g. standard epinephrine, corticosteroid, and antihistamine therapy). Drug-related deaths associated with platinum compounds from this reaction have been reported.

Neurological: An acute, reversible primarily peripheral sensory neuropathy of early onset, occurring within hours or one to two days of dosing, resolves within 14 days, and frequently recurs with further dosing. The symptoms may be precipitated or exacerbated by exposure to cold temperature or cold objects and they usually present as transient paresthesia, dysesthesia and hypoesthesia in the hands, feet, perioral area, or throat. Jaw spasm, abnormal tongue sensation, dysarthria, eye pain, and a feeling of chest pressure have also been observed. The acute, reversible pattern of sensory neuropathy was observed in about 56% of study patients who received oxaliplatin with infusional 5-FU/LV. In any individual cycle acute neurotoxicity was observed in approximately 30% of patients. Ice (stomatitis prophylaxis) should be avoided during the infusion of oxaliplatin because cold temperature can exacerbate acute neurological symptoms.

An acute syndrome of pharyngolaryngeal dysesthesia seen in 1-2% of patients is characterized by subjective sensations of dysphagia or dyspnea, without any laryngospasm or bronchospasm (no stridor or wheezing).

A persistent (>14 days), primarily peripheral, sensory neuropathy that is usually characterized by paresthesias, dysethesias, hypoesthesias, but may also include deficits in proprioception that can interfere with daily activities (e.g., writing, buttoning, swallowing, and proprioception-related ambulatory impairment). These forms of neuropathy occurred in 48% of the study patients receiving oxaliplatin with infusional 5-FU/LV. Persistent neuropathy can occur without any prior acute neuropathy event. The majority of the patients (80%) who developed Grade 3 persistent neuropathy progressed from prior Grade 1 or 2 events. These symptoms may improve in some patients upon discontinuation of oxaliplatin.

Pulmonary: Oxaliplatin has been associated with pulmonary fibrosis (0.7% of study patients), which may be fatal. In case of unexplained respiratory symptoms such as non-productive cough, dyspnea, crackles, or radiological pulmonary infiltrates, oxaliplatin should be discontinued until further pulmonary investigation excludes interstitial lung disease or pulmonary fibrosis.

Pregnancy and Lactation (see also Section 7.2.5): To date there is no available information on safety of use in pregnant women. Based on pre-clinical findings, oxaliplatin is likely to be lethal and/or teratogenic to the human fetus at the recommended therapeutic dose, and is therefore not recommended during pregnancy. Patients becoming pregnant during the study should be withdrawn from the study treatment. Excretion in

breast milk has not been studied. Breast-feeding is contraindicated during oxaliplatin therapy.

7.5.4 5-FU/LV Precautions

Fluorouracil should be given with care to weak or malnourished patients, to those with a history of heart disease, or to those with hepatic or renal insufficiency.

The main adverse effects of fluorouracil are on the bone marrow and the gastro-intestinal tract, and may be dose-limiting. Toxicity is schedule dependent: reducing the rate of injection to a slow infusion is associated with less haematological toxicity but does not decrease gastro-intestinal toxicity. With protracted continuous infusion in particular, the hand-foot syndrome may occur. Gastro-intestinal toxicity may be exacerbated if fluorouracil is given with folinic acid.

Hematological: Depending upon schedule used, the nadir of the white cell count may occur from 7 to 20 days after a dose. Thrombocytopenia is usually at a maximum 7 to 17 days after a dose. Anemia may also occur.

Gastro-intestinal: Stomatitis, gastro-intestinal ulceration and bleeding, diarrhoea, or haemorrhage from any site are signs that treatment should be stopped if appropriate (see Section 7.3.5). Nausea and vomiting are common.

Coagulopathy: Caution should be taken with the concomitant use of coumarin-derived anticoagulants, as warfarin interaction has been documented in the literature [81].

Cardiovascular: Life-threatening cardiotoxicity (arrhythmias, ventricular tachycardia, and cardiac arrest, secondary to transmural ischaemia) has been reported to occur in 0.55% of patients given fluorouracil, although the incidence of angina and less severe cardiotoxicity associated with coronary artery spasm may be higher. Myocardial ischaemia has occurred. Possible risk factors include pre-existing heart disease or mediastinal radiotherapy and administration by prolonged infusion, but symptoms can also occur in patients without these risk factors.

Neurological: Central neurotoxicity, including cerebellar ataxia, confusion, disorientation, and emotional lability is reported to occur rarely in patients receiving fluorouracil.

Skin: Hand-foot syndrome (palmar-plantar erythrodysesthesia) has been reported. Although particularly associated with administration by protracted infusion, the syndrome may also occur following bolus doses. Symptoms generally resolve upon discontinuation of the drug. In addition, local inflammatory and photosensitivity reactions have occurred following topical use. Dermatitis and rarely, erythema multiforme have been reported.

Ocular: Systemic fluorouracil therapy has been associated with various types of ocular toxicity including several cases of excessive lacrimation, watering of the eyes or corneal epithelial erosion.

8. STATISTICAL CONSIDERATIONS AND ANALYTICAL PLAN

8.1 Primary and Secondary Study Variables

8.1.1 Primary Variable

The primary endpoint of the study is disease-free survival (DFS). This is defined as the time from the date of randomization to the time of a recurrence, a new occurrence of colorectal cancer or death due to any cause. Patients without an event will be censored at the last date the patient was known to be disease-free. Recurrence and a new occurrence of colorectal cancer will be based on tumour assessments made by the investigators.

8.1.2 Secondary Variable

Overall survival is defined as the time between date of randomization and date of death due to any cause. Patients who were not reported as having died at the time of the analysis will be censored at the date they were last known to be alive.

8.1.3 Safety Variables

- Incidence of adverse events and severe adverse events,
- Time to onset of adverse events,
- Long-term safety in terms of targeted events,
- Laboratory parameters,
- Premature withdrawals,
- Vital signs,
- Total cumulative dose, received versus planned dose,
- Incidence and timing of dose reductions.

8.1.4 Tertiary Variables

- Medical care utilization,
- CCSQ (Chemotherapy Convenience and Satisfaction Questionnaire).

8.2 Statistical and Analytical Methods

8.2.1 Primary Analysis

Disease-free survival (DFS) is the primary endpoint of the study and will be used to assess superiority of bevacizumab in combination with FOLFOX-4 over FOLFOX-4 alone as well as to assess superiority of bevacizumab in combination with XELOX over FOLFOX-4 alone.

The evaluation of disease-free survival will be performed using a two-sided log rank test (not stratified) based on patients with stage III disease.

Median disease-free survival time and the 95% confidence limits will be estimated using Kaplan-Meier survival methodology. Plots of the Kaplan-Meier estimates of disease-free survival for each treatment group will also be produced together with corresponding three-year disease-free survival rates. Estimates of the treatment effects will be expressed as hazard ratios including 95% confidence intervals. The assumptions of proportional hazard will be checked. All these analyses will be based on patients with stage III disease.

8.2.2 Secondary Analyses

For overall survival, the same analyses will be performed as for the primary parameter disease-free survival.

An exploratory Cox regression analysis will be performed to explore XELOX+bevacizumab versus FOLFOX-4+bevacizumab. Hazard ratio and 95% confidence intervals for the hazard ratio will be calculated to investigate whether the combination of bevacizumab and XELOX is at least as efficacious as the combination of bevacizumab and FOLFOX-4 in terms of disease-free survival and overall survival.

8.2.3 Exploratory Analyses

DFS will be analyzed separately for the pool of patients (high-risk stage II and stage III disease patients combined) and for patients with high-risk stage II disease only. Median disease-free survival time and the 95% confidence limits will be estimated using Kaplan-Meier survival methodology. Plots of the Kaplan-Meier estimates of disease-free survival for each treatment group will also be produced together with corresponding three-year disease-free survival rates. Estimates of the treatment effects will be expressed as hazard ratios including 95% confidence intervals. These analyses are regarded to be of exploratory manner only.

For DFS, the retained effect of bevacizumab + XELOX versus bevacizumab + FOLFOX-4 given the effect of bevacizumab + FOLFOX-4 over FOLFOX-4 alone will also be reported for exploratory purposes.

Further subgroup analyses on DFS will be performed by prognostic factors to assess internal consistency. Factors include albumin, alkaline phosphatase, gender, age, race.

Disease-free survival as well as overall survival will be analyzed using the Cox regression model adjusted for treatment and baseline prognostic factors (e.g. influence of early versus late initiation of adjuvant treatment).

An additional analysis will be performed on relapse-free survival (RFS). Therein, deaths unrelated to colorectal cancer will not be regarded as events.

Incomplete follow-up will be summarized per treatment group for all patients as well as only for patients with an event and only those without an event to contrast the non-compliance in terms of follow-up visits. A sensitivity analysis will be performed on compliant patients only. In case of imbalances between the treatment groups the following imputation algorithm will be applied: for patients with missing follow-up visits and subsequent events the date of event will be imputed to be the scheduled date of the

missed follow-up visit in case there was a missing visit directly before the event. This sensitivity analysis will be performed if the between-treatment group difference in the proportion of patients with a DFS event who meet the definition of a missed evaluation is > 5% or if the overall proportion is > 10%.

The extent of follow-up information for disease-free survival in each treatment group will be investigated by analyzing the time to censoring. This analysis follows the algorithm of disease-free survival with the difference that patients who had an event will be censored in this analysis at the date of their event and patients without an event will be regarded as having had an event at the censoring date.

8.2.4 Safety Variables

All safety parameters will be analyzed and presented in terms of listings and summary tables. The analyses will be based on the safety population.

8.2.4.1 Adverse Events, Laboratory Data, Premature Withdrawals

All adverse events occurring up to 28 days after end of study treatment phase will be recorded in the case report form. Targeted adverse events (i.e. proteinuria, hypertension and wound healing complications) will be reported and followed as outlined in section 7.2.3.2. Adverse events and laboratory parameters will be assessed according to the CTCAE v 3.0 [89]. Adverse events will be reported as listings and summarized as frequency tables. Additional presentations will include summaries by severity and relationship to study treatment. Where applicable, similar summaries will be presented for targeted adverse events as well as for gastrointestinal perforations, bleeding and thromboembolic events. In addition, the incidence of surgical procedures associated with wound healing disturbances as well as the incidence and outcome of hypertension and proteinuria will be analyzed. Exploratory analyses of the impact of risk factors on the development of specific adverse events will also be performed using Cox regression models.

Additional analyses will investigate the time to the first onset of any of the targeted adverse events. Hazard ratios, medians and associated 95% confidence intervals will be reported for each study arm. The time to the first onset of adverse events will be measured as the time from treatment start to the date of the first occurrence of any of the adverse events under investigation. Patients without any of these adverse events will be censored at the date that corresponds to 28 days after end of study treatment phase. Time to the first onset analyses consider the frequency as well as the timing of adverse events and are therefore deemed more powerful than analyses of frequency rates alone.

For laboratory parameters, descriptive summary tables of change from baseline over time will be produced. Withdrawals of patients from study medication will be reported as listings and summary tables.

8.2.4.2 Vital Signs

Descriptive summary tables of change from baseline over time will be provided for vital signs.

8.2.4.3 Cumulative Dose, Received versus Planned Dose

Listings showing the drug intake for each patient will be provided. The mean total cumulative dose will be summarized for each treatment arm (separately for each agent).

In addition, for each study arm the received dose will be compared to the planned dose. The planned dose is defined as the dose that would be given if no doses were missed and/or no dose reductions were made for the number of cycles started.

8.2.5 Other Analyses

8.2.5.1 Pharmacoeconomic Analysis

Information obtained from the collection of medical care utilization data in this study will be reported separately from the clinical study report of this study. An analysis plan for the analysis of these pharmacoeconomic data will also be generated separately. Pharmacoeconomic data may be combined with other data such as cost data or other clinical parameters in the production of a final pharmacoeconomic report.

Medical care utilization – the analysis and reporting of resource use data will be handled separately from the clinical study report.

8.2.5.2 Chemotherapy Convenience and Satisfaction Assessment

Analyses are considered to be of exploratory manner only.

8.2.6 Hypothesis testing

The primary analysis is a two-sided log rank test at the 5% alpha level for the following hypotheses, that the survival distribution of disease-free survival of the bevacizumab-FOLFOX-4 treatment group is the same as for the FOLFOX-4 treatment group versus the alternative that the two distributions are different:

H_0: SDD (bevacizumab + FOLFOX-4) = SDD (FOLFOX-4)
versus
H_1: SDD (bevacizumab + FOLFOX-4) $\neq$ SDD (FOLFOX-4)
where SDD denotes the survival distribution of the parameter disease-free survival.

In addition, the same hypotheses for the bevacizumab-XELOX treatment group versus the FOLFOX-4 treatment group will be tested in the same way:
H_0: SDD (bevacizumab + XELOX) = SDD (FOLFOX-4)
versus
H_1: SDD (bevacizumab + XELOX) $\neq$ SDD (FOLFOX-4)
where SDD denotes the survival distribution of the parameter disease-free survival.
Adjustments for multiplicity will be done using a closed test procedure which tests for differences between all three treatment groups at the 5% alpha level first and only in case of a significant result tests further the two hypotheses defined above, again at the 5% alpha level:

1. The closed family of null hypotheses related to the elementary null hypotheses H_{0i}=[1i]: SDD(1) = SDD(i) (i=2,3) where SDD denotes the survival distribution of disease-free survival is generated by first intersecting all pairs of elementary hypotheses. Then the resulting hypotheses are again intersected. The intersection procedure is repeated until only one hypothesis (the global hypothesis) is generated.
2. For every member of the closed family the p-value of the two-sided log rank test is calculated.
3. According to the closure principle of Marcus, Peritz and Gabriel [87] a hypothesis of the closed family can only be rejected if the corresponding test is significant at a given level α and any other hypothesis in the family that implies it has also been rejected by its corresponding α-level test. We will use the significance level α=0.05. The closure principle will be followed by calculating for every hypothesis the adjusted p-value as the maximum of the p-values of all hypotheses implying it.

The adjusted p-values p* are computed as follows:

Hypothesis	Adjusted p-value
[123] : SDD(1) = SDD(2) = SDD(3)	$p^*_{[123]} = \max(p_{[123]})$
[12] : SDD(1) = SDD(2)	$p^*_{[12]} = \max(p_{[123]}, p_{[12]})$
[13] : SDD(1) = SDD(3)	$p^*_{[13]} = \max(p_{[123]}, p_{[13]})$

A hypothesis will only be rejected if the adjusted p-value is smaller than α=0.05.

For completeness reasons a two-sided comparison of bevacizumab + XELOX vs. bevacizumab + FOLFOX-4 will be performed as part of the closed testing procedure:

H_0: SDD (bevacizumab + XELOX) = SDD (bevacizumab + FOLFOX-4)
versus
H_1: SDD (bevacizumab + XELOX) ≠ SDD (bevacizumab + FOLFOX-4)
where SDD denotes the survival distribution of the parameter disease-free survival. The adjusted p-value will be calculated as $p^*_{[23]}$ = max ($p_{[123]}$, $p_{[23]}$). The hypothesis will be rejected if the adjusted p-value is smaller than α=0.05.

This procedure controls the multiple α or family-wise error rate [88] and will ensure an overall alpha level of 5%.

8.2.7 Types of Analyses

Assignment of patients to any of the three populations mentioned below will be performed prior to database closure.

The primary efficacy analysis will be performed both on all randomized patients (intent-to-treat population) and on the per protocol population.

Secondary and exploratory efficacy analyses will be performed on the intent-to-treat population. Non-inferiority analyses will be repeated on the per protocol population.

8.2.7.1 Intent-to-Treat Population

All patients who were randomized to one of the three study arms will be included in the intent-to-treat population (all randomized patients) and presented according to the therapy that they were randomized to receive.

8.2.7.2 Per-Protocol Population

The per-protocol population excludes patients randomized who did not receive at least one dose of capecitabine, 5-FU, oxaliplatin, or bevacizumab or who had a major violation of protocol inclusion or exclusion criteria. Patients will be presented according to the therapy that they were randomized to receive.

8.2.7.3 Safety Population

All patients who received at least one dose of capecitabine, 5-FU, oxaliplatin or bevacizumab will be included in the safety population. The safety population will be used for the analysis of all safety parameters. Patients are assigned to treatment groups based on what they actually received.

8.2.8 Exclusion of Data from Analysis

No data will be excluded.

8.2.9 Interim Analysis

No interim analysis of efficacy endpoints is planned. An independent Data & Safety Monitoring Board (DSMB) will be set up to monitor the safety data throughout the study.

8.3 Sample Size

This is an event driven trial. The study will continue until approximately 836 events have occurred in patients with stage III disease.

The primary endpoint of disease-free survival was used to assess the sample size. The sample size calculation is based on stage III patients due to the fact that the primary analysis will be performed on patients with stage III disease only. Assuming for patients with stage III disease a 23% reduction in the hazard rate of the bevacizumab-FOLFOX-4 treatment arm over the FOLFOX-4 treatment group or a 23% reduction in the hazard rate of the bevacizumab-XELOX treatment arm over the FOLFOX-4 treatment arm 2880 patients (960 in each treatment arm) will provide 836 events, which should be sufficient to yield 80% power for a two-sided log rank test at an alpha level of 0.025. This will at the same time also guarantee 80% power for a two-sided log rank test at an alpha level of 5% using a closed test procedure for the adjustment for multiplicity. In addition to patients with stage III disease, patients with high-risk stage II disease will be recruited for exploratory analyses. The number of these patients will be restricted to approximately 16% of all patients. In total, 3450 patients will be randomized.

To calculate the number of events required in this study, the following assumptions were made:

- Primary analysis will be performed on stage III patients only.
- Two-sided log rank test.
- 80% power.
- 2.5% significance level.
- Hazard ratio of bevacizumab in combination with FOLFOX-4 versus FOLFOX-4 alone of 0.77 corresponding to an improvement in the 3 year disease-free survival rate from 72.2% to 77.8%.
- Drop-out rate of 10% of all patients after 36 months.
- Exponential distribution.

Assuming a recruitment rate of 150 patients per month, a recruitment period of 23 months and a minimal follow-up period of 36 months, 3450 patients will provide 836 events in stage III patients, which should be sufficient to yield 80% power by the primary closed testing procedure described above.

The assumption of a 3 year disease-free survival rate for the FOLFOX-4 treatment arm of 72.2% for stage III patients is based on the information from the MOSAIC trial [23].

The required number of events and the number of patients was determined using nQuery Advisor Version 5.0.

8.4 Replacement Policy (Ensuring Adequate Numbers of Evaluable Patients)

8.4.1 For Patients

No patient prematurely discontinued from the study for any reason will be replaced.

8.4.2 For Centres

A centre may be replaced for the following administrative reasons:

- Excessively slow recruitment.
- Poor protocol adherence.

9. DATA QUALITY ASSURANCE

The overall procedures for quality assurance of clinical study data are described in the applicable Roche Standard Operating Procedures (SOPs).

Accurate and reliable data collection will be assured by verification and cross–check of the CRFs against the investigator’s records by the study monitor (source document verification), and the maintenance of a drug–dispensing log by the investigator.

The data collected will be entered into the study database from the working copy of the CRF sent from the site.

A comprehensive validation check program will verify the data and discrepancy reports will be generated accordingly for resolution by the investigator. As patients complete the

study (or prematurely withdraw) and their signed CRFs become available, a second data entry will be performed from the original, signed CRF. A comparison check will be run to identify and resolve any discrepancies between the first and second data entry.

Throughout the study the Study Management Team will review data according to the SMT Data Review Plan as described in the Data Quality Plan.

10. INDEPENDENT DATA AND SAFETY MONITORING BOARD

A group of independent experts will form an independent DSMB. The committee will be responsible for monitoring on an approximately semi annual basis the safety data of the clinical trial and the safety of the patients. The drug safety committee will provide recommendations on the progress of the trial and the safety of the patients after each meeting. This review will continue until the last patient has finished treatment. This group will consist of independent experts not involved in the trial, including 1 statistician, 2 practicing oncologists, 1 surgeon with experience in colon cancer surgery and an expert in clinical research ethics. Furthermore, representatives from Hoffmann La-Roche may also participate in the open session of DSMB meetings as non-voting members in order to discuss any question that might arise; however, the representatives from Hoffmann La-Roche will not be allowed to participate at the closed DSMB sessions. The sponsor representatives shall also not review and discuss the data provided to DSMB.

For the safety review, the independent DSMB will be provided with summary tables on demographic data, adverse events, serious adverse events, laboratory abnormalities, shifts in laboratory values, withdrawals from study medication and deaths by treatment arm.

Further information (e.g., summaries of baseline characteristics by treatment arm) may be given to the DSMB upon request. The necessary safety analyses will be carried out by a study independent statistician.

The first 300 patients to be enrolled on the study will be monitored for safety in real time by an independent DSMB. The independent DSMB will review safety listings until the 300th patient has completed 6 weeks of treatment i.e. 3 or 2 cycles completed. In case the safety profile is confirmed after approx. 100 patients on each of the three arms have completed six weeks of treatment, the independent DSMB will follow all patients on a six-monthly basis.

Real time safety monitoring by the independent DSMB will include the following:

- CTCAE Grade 3 and 4 AEs
- SAEs
- Any AE requiring dose interruption, reduction or discontinuation of study drugs
- Demographic data
- Death

Details on the roles and responsibilities of the independent Data and Safety Monitoring Board will be given in a separate charter.

11. REFERENCES

1. SEER Cancer Statistics Review 1973-1999. http://seer.cancer.gov/csr/1973_1999/colorect.pdf
2. Verschraegen CF, Pazdur R. Medical management of colorectal carcinomas. Tumouri 1994; 80: 1-11.
3. Kemeny N. Current approaches to metastatic colorectal cancer. Semin Oncol 1994; 21(4 suppl 7): 67-75.
4. Wolmark N, Rockette H, Mamounas E, et al. Clinical trial to assess the relative efficacy of fluorouracil and leucovorin, fluorouracil and levamisole, and fluorouracil, leucovorin, and levamisole in patients with Dukes' B and C carcinoma of the colon: results from National Surgical Adjuvant Breast and Bowel Project C-04. J Clin Oncol 1999, 17: 3553-3559.
5. Porschen R, Bermann A, Loffler T, et al. Fluorouracil plus leucovorin as effective adjuvant chemotherapy in curatively resected stage III colon cancer: results of the trial adjCCA-01. J Clin Oncol 2001, 19: 1787-1794..
6. Graf W, Pahlman L, Bergstrom R, Glimelius B. The relationship between an objective response to chemotherapy and survival in advanced colorectal cancer. Br J Cancer 1994; 70: 559-563.
7. Lokich JJ, Ahlgren JD, Gullo JJ, et al. A prospective randomized comparison of continuous infusion fluorouracil with a conventional bolus schedule in metastatic colorectal carcinoma: a Mid-Atlantic Oncology Program Study. J Clin Oncol 1989; 7: 425-432.
8. Leichman CG, Leichman L, Spears CP, Rosen PJ, Jeffers S, Groshen S. Prolonged continuous infusion of fluorouracil with weekly bolus leucovorin: a phase II study in patients with disseminated colorectal cancer. J Natl Cancer Inst 1993; 85: 41-44.
9. Hansen RM. 5-Fluorouracil by protracted venous infusion: a review of recent clinical studies. Cancer Investigation 1991; 9: 637-642.
10. Mori A, Bertoglio S, Guglielmi A, et al. Activity of continuous-infusion 5-fluorouracil in patients with advanced colorectal cancer clinically resistant to bolus 5-fluorouracil. Cancer Chemother Pharmacol 1993; 33: 179-180.
11. Saltz LB, Cox JV, Blanke C, et al. Irinotecan plus fluorouracil and leucovorin for metastatic colorectal cancer. Irinotecan Study Group. N Engl J Med 2000; 343(13): 905-914.
12. Douillard JY, Cunningham D, Roth AD, et al. Irinotecan combined with fluorouracil compared with fluorouracil alone as first-line treatment for metastatic colorectal cancer: a multicentre randomized trial. Lancet 2000; 355 (9209): 1041-1047.

13. de Gramont A, Figer A, Seymour M, et al. Leucovorin and fluorouracil with or without oxaliplatin as first-line treatment in advanced colorectal cancer. J Clin Oncol 2000; 18: 2938-2947.

14. Moertel CG, Fleming TR, Macdonald JS et al. Levimasol and fluorouracil for adjuvant therapy of resected colon carcinoma. N Engl J Med 1990; 322: 352-358.

15. Moertel CG, Fleming TR, Macdonald JS et al. Intergroup study of fluorouracil plus levimasol as adjuvant therapy for stage II/Dukes' B2 colon cancer. J Clin Oncol 1995; 13: 2936-2943.

16. International Multicentre Pooled Analysis of Colorectal Cancer Trials (IMPACT). Efficacy of adjuvant fluorouracil and folinic acid in colon cancer. Lancet 1995; 345: 939-944.

17. O'Connell MJ, Mailliard JA, Kahn MJ et al. Controlled trial of fluorouracil and low-dose leucovorin given for 6 months as postoperative adjuvant therapy for colon cancer. J Clin Oncol 1997; 15:246-50.

18. O'Connell MJ, Laurie JA, Kahn M et al. Prospectively randomized trial of postoperative adjuvant chemotherapy in patients with high-risk colon cancer. J Clin Oncol 1998; 16: 295-300.

19. Haller D, Catalano P, MacDonald J, Mayer R. Fluorouracil (FU), leucovorin (LV) and levimasol (LEV) adjuvant therapy for colon cancer: five-year final report of INT-0089. Proc Am Soc Clin Oncol 1998; 17: 256a (abstract 982).

20. Van Cutsem E, Dicato M, Wils J, Cunningham D, Diaz-Rubio E, Glimelius B, Haller D, Johnston P, Kerr D, Koehne CH, Labianca R, Minsky B, Nordlinger B, Roth A, Rougier P, and Schmoll HJ. Adjuvant treatment of colorectal cancer (current expert opinion derived from the Third International Conference: Perspectives in Colorectal Cancer, Dublin, 2001). Eur J Cancer 2002; 38: 1429-1436.

21. de Gramont A, Bosset JF, Milan C, et al. Randomized trial comparing low-dose leucovorin and fluorouracil bolus with bimonthly high-dose leucovorin and fluorouracil bolus plus continuous infusion for advanced colorectal cancer: a French intergroup study. J Clin Oncol 1997; 15: 808-815.

22. Andre T, Colin P, Louvet C, Gamelin E, Bouche O, Achille E, Colbert N, Boaziz C, Piedbois P, Tubiana-Mathieu N, Boutan-Laroze A, Flesch M, Buyse M, de Gramont A.Semimonthly versus monthly regimen of fluorouracil and leucovorin administered for 24 or 36 weeks as adjuvant therapy in stage II and III colon cancer: results of a randomized trial. J Clin Oncol. 2003; 21: 2896-903.

23. Andre T, Boni C, Mounedji-Boudiaf L, Navarro M, Tabernero J, Hickish T, Topham C, Zaninelli M, Clingan P, Bridgewater J, Tabah-Fisch I, de

Gramont A. Oxaliplatin, Fluorouracil, and Leucovorin as Adjuvant Treatment for Colon Cancer. N Engl J Med 2004;350:2343-51.

24. Borgström P, Gold DP, Hillan KJ, Ferrara N. Importance of VEGF for breast cancer angiogenesis in vivo: implications from intravital microscopy of combination treatments with an anti-VEGF neutralizing monoclonal antibody and doxorubicin. Anticancer Res 1999; 19:4203–14.

25. Christian B. 26-Week intravenous toxicity study with rhuMAb VEGF in cynomolgus monkeys with a 12-week recovery. South San Francisco (CA): Genentech; 1999 Report No.: 97-194-1751.

26. Dvorak HF, Brown LF, Detmar M, Dvorak AM. Vascular permeability factor/vascular endothelial growth factor, microvascular hyperpermeability, and angiogenesis. Am J Pathol 1995; 146: 1029–39.

27. Ferrara N, Davis-Smyth T. The biology of vascular endothelial growth factor. Endocr Rev 1997;18:4–25.

28. Ferrara N, Chen H, Davis-Smyth T, Gerber HP, Nguyen TN, Peers D, et al. Vascular endothelial growth factor is essential for corpus luteum angiogenesis. Nat Med 1998; 4: 336–40.

29. Hurwitz H, Fehrenbacher L, Cartwright T, et al Bevacizumab (a monoclonal antibody to vascular endothelial growth factor) prolongs survival in first-line colorectal cancer (CRC): Results of a phase III trial of bevacizumab in combination with bolus IFL (irinotecan, 5-fluorouracil, leucovorin) as first-line therapy in patients with metastatic CRC ASCO 2003 Abstract No: 3646.

30. Giantonio BJ, Catalano PJ, Meropol NJ, et al. The addition of bevacizumab (anti-VEGF) to FOLFOX4 in previously treated advanced colorectal cancer (advCRC): An updated interim toxicity analysis of the Eastern Cooperative Oncology Group (ECOG) study E3200. Proc ASCO GI 2004 Abstract No: 241

31. Hurwitz H, Fehrenbacher L, Cartwright T, et al. A phase III trial of bevacizumab (Avastin) in combination with bolus IFL (irinotecan, 5-fluorouracil, leucovorin) (5-FU/LV/BV) and other exploratory analyses [abstract]. American Association for Cancer Research July 2003 [online].

32. Data on file

33. Leach W, Shopp G. Safety evaluation of rhuMAb VEGF in a rabbit dermal wound model. South San Francisco (CA): Genentech; 1997 Report No.: 96-407-1751.

34. Population Pharmacokinetics of Bevacizumab following Multiple Dosing of Bevacizumab in Phase I-III Studies. Report No. 03-0324-1751.

35. Barron H, Fyfe G. A phase III, multicenter, randomized, active controlled clinical trial to evaluate the efficacy and safety of rhuMAb VEGF

(Bevacizumab) in combination with standard chemotherapy in subjects with metastatic colorectal cancer. Genentech report AVF2107g, August 2003.

36. Lieberman G, Gaudreault J, Novotny William. A phase III, multidose, randomized, multicenter clinical trial to evaluate the efficacy, safety, and pharmacokinetics of recombinant humanized monoclonal anti-VEGF antibody (rhuMAb VEGF) combined with 5-fluorouracil and leucovorin chemotherapy in patients with metastatic colorectal cancer. Genentech report AVF0780g, March 2002.

37. Presta LG, Chen H, O'Connor SJ, Chisholm V, Meng YG, Krummen L, et al. Humanization of an anti-vascular endothelial growth factor monoclonal antibody for the therapy of solid tumours and other disorders. Cancer Res 1997; 57: 4593–9.

38. Radinsky R, Ellis LM. Molecular determinants in the biology of liver metastasis. Surg Oncol Clin N Am 1996; 5: 215–29.

39. Ryan AM, Eppler DB, Hagler KE, Bruner RH, Thomford PJ, Hall RL, et al. Preclinical safety evaluation of rhuMAbVEGF, an antiangiogenic humanized monoclonal antibody. Toxicol Pathol 1999; 27: 78–86.

40. Takahashi Y, Tucker SL, Kitadai Y, Koura AN, Bucana CD, Cleary KR, et al. Vessel counts and expression of vascular endothelial growth factor as prognostic factors in node-negative colon cancer. Arch Surg 1997; 132: 541–6.

41. Takahashi Y, Kitadai Y, Bucana CD, Cleary KR, Ellis LM. Expression of vascular endothelial growth factor and its receptor, KDR, correlates with vascularity, metastasis, and proliferation of human colon cancer. Cancer Res 1995; 55: 3964-8.

42. Tokunaga T, Oshika Y, Abe Y, Ozeki Y, Sadahiro S, Kijima H, et al. Vascular endothelial growth factor (VEGF) mRNA isoform expression pattern is correlated with liver metastasis and poor prognosis in colon cancer. Br J Cancer 1998;77:998–1002.

43. Warren RS, Yuan H, Matli MR, Gillett NA, Ferrara N. Regulation by vascular endothelial growth factor of human colon cancer tumourigenesis in a mouse model of experimental liver metastasis. J Clin Invest 1995; 95: 1789–97.

44. Zebrowski BK, Liu W, Ramirez K, Akagi Y, Mills GB, Ellis LM. Markedly elevated levels of vascular endothelial growth factor in malignant ascites. Ann Surg Oncol 1999; 6: 373–8.

45. Howdieshell TR, Riegner C, Gupta V, Callaway D, Grembowicz K, Sathyanarayana, McNeil PL. Normoxic wound fluid contains high levels of vascular endothelial growth factor. Ann Surg. 1998; 228: 707-15.

46. Nissen NN, Polverini PJ, Koch AE, Volin MV, Gamelli RL, DiPietro LA. Vascular endothelial growth factor mediates angiogenic activity during the proliferative phase of wound healing. Am J Pathol. 1998; 152: 1445-52.

47. Tonnesen MG, Feng X, Clark RA.Angiogenesis in wound healing. J Investig Dermatol Symp Proc. 2000; 5: 40-6.

48. Ferrara N. Molecular and biological properties of vascular endothelial growth factor. J Mol Med. 1999; 77: 527-543.

49. Gordon MS. Margolin K. Talpaz M. Sledge GW Jr. Holmgren E. Benjamin R. Phase I safety and pharmacokinetic study of recombinant human anti-vascular endothelial growth factor in patients with advanced cancer. J Clin Oncol 2001; 19: 843–50.

50. Margolin K, Gordon MS, Holmgren E, Gaudreault J, Novotny W, Fyfe G, et al. Phase Ib trial of intravenous recombinant humanized monoclonal antibody to vascular endothelial growth factor in combination with chemotherapy in patients with advanced cancer: pharmacologic and long-term safety data. J Clin Oncol 2001; 19: 851–6.

51. Recombinant Humanized Monoclonal Anti-VEGF Antibody (rhuMab VEGF, Bevacizumab) Investigators' Brochure, Genentech, Inc., 16 September 2002.

52. Kabbinavar F, Hurwitz HI, Fehrenbacher L, Meropol NJ, Novotny WF, Lieberman G, et al. Phase II, randomized trial comparing bevacizumab plus fluorouracil (FU)/leucovorin (LV) with FU/LV alone in patients with metastatic colorectal cancer. J Clin Oncol. 2003; 21: 60-5.

53. Miwa M, Ura M, Nishida M, et al. Design of a novel oral fluoropyrimidine carbamate, capecitabine, which generates 5-fluorouracil selectively in tumours by enzymes concentrated in human liver and cancer tissue. Eur J Cancer 1998; 34(8):1274–1281.

54. Reigner B, Blesch K, Weidekamm E: Capecitabine; a review of its clinical pharmacokinetics. Clin Pharmacokinet 2001; 40:85–104.

55. Wiernik PH, Yeap B, Vogl SE, Kaplan BH, Comis RL, Falkson G, Davis TE, Fazzini E, Cheuvart B, Horton J. Hexamethylmelamine and low or moderate dose cisplatin with or without pyridoxine for treatment of advanced ovarian carcinoma: a study of the Eastern Cooperative Oncology Group.Cancer Invest. 1992;10: 1-9.

56. Gieschke R, Reigner B, Blesch KS, Steimer JL. Population pharmacokinetic analysis of the major metabolites of capecitabine. J Pharmacokinet Pharmacodyn. 2002; 29: 25-47.

57. Twelves C, Glynne-Jones R, Cassidy J et al. Effect of hepatic dysfunction due to liver metastases on the pharmacokinetics of capecitabine and its metabolites. Clin Cancer Res 1999; 5:1696–1702.

58. Poole C et al. Effect of renal impairment on the pharmacokinetics and tolerability of capecitabine (Xeloda) in cancer patients. Cancer Chemother Pharmacol 2002; 49: 225-234.

59. Van Cutsem E, Findlay M, Osterwalder B, et al. Capecitabine, an oral fluoropyrimidine carbamate with substantial activity in advanced colorectal cancer: results of a randomized phase II study. J Clin Oncol 2000; 18: 1337-1345.

60. Hoff PM, Ansari R, Batist G, et al. Comparison of oral capecitabine versus intravenous Fluorouracil plus Leucovorin as first line treatment in 605 patients with Metastatic colorectal cancer: results of a randomized phase III study. J Clin Oncol 2001; 19: 2282-2292.

61. Van Cutsem E, Twelves C, Cassidy J, et al. Oral capecitabine compared with intravenous Fluorouracil plus Leucovorin in patients with Metastatic colorectal cancer: results of a large phase III study. J Clin Oncol 2001, 19, 4097-4106.

62. Van Cutsem E, Hoff PM, Harper P, et al. Oral capecitabine vs intravenous 5-FU and leucovorin: integrated efficacy data and novel analyses from two large randomized, phase III trials. Br J Cancer 2004; 90: 1190–1197.

63. Cassidy J, Twelves C, Van Cutsem E, et al. First-line oral capecitabine therapy in metastatic colorectal cancer: a favorable safety profile compared with intravenous 5-fluourouracil/leucovorin. Ann Oncol 2002; 13: 566-575.

64. Scheithauer W, McKendrick J, Begbie S, Borner M, et al.; X-ACT Study Group. Oral capecitabine as an alternative to i.v. 5-fluorouracil-based adjuvant therapy for colon cancer: safety results of a randomized, phase III trial. Ann Oncol. 2003; 14: 1735-43.

65. Sanofi-Synthelabo. Eloxatin: Summary of product characteristics. France; August 2001.

66. Raymond E, Chaney SG, Taama A, et al. Oxaliplatin a review of preclinical and clinical studies. Ann Oncol 1998, 9: 1053-1071.

67. Machover D, Diaz-Rubio E, et al. Two consecutive phase II studies of Eloxatin (L-OHP) for treatment of patients with advanced colorectal carcinoma who were resistant to previous treatment with fluoropyrimidines. Ann Oncol 1996; 7: 95-98.

68. Garufi D, Lévi F, Misset JL. Activity of Eloxatin in colonic cancer: a report of 25 demonstrative cases. Debiopharm Internal Report, October 1994.

69. Lévi F, Zidani R, Misset JL. Randomized, multicenter trial of chronotherapy with Eloxatin, fluorouracil and folinic acid in metastatic colorectal cancer. Lancet 1997; 350: 681-686.

70. Andre T, Bensmaine MA, Louvet C, et al. Multicenter Phase II Study of Bimonthly High-Dose Leucovorin, Fluorouracil Infusion, and Oxaliplatin for Metastatic Colorectal Cancer Resistant to the Same Leucovorin and Fluorouracil Regimen; J Clin Oncol 1999, 17: 3560-3568.

71. Grothey A, Deschler B, Kroening H, et al. Phase III study of bolus 5-fluorouracil (5-FU)/ folinic acid (FA) (Mayo) vs weekly high-dose 24h 5-FU infusion/ FA + oxaliplatin (OXA) (FUFOX) in advanced colorectal cancer (ACRC). Proc Am Soc Clin Oncol 2002; 21: 128a, abstract 512 (oral presentation slides).

72. Goldberg RM, Sargent DJ, Morton RF, Fuchs CS, Ramanathan RK, Williamson SK, Findlay BP, Pitot HC, Alberts SR. A randomized controlled trial of fluorouracil plus leucovorin, irinotecan, and oxaliplatin combinations in patients with previously untreated metastatic colorectal cancer. J Clin Oncol. 2004; 22: 23-30.

73. Lévi F, Misset JL, Brienza D, Adam R, Metzger G, Itzhaki M, et al. A chronopharmacologic phase II clinical trial with 5-fluorouracil, folinic acid, and Eloxatin using an ambulatory multichannel programmable pump. Cancer 1992; 69: 893-900.

74. Eloxatin® US package insert, August 2002.

75. Wolmark N, Colangelo L, Wieand S. National Surgical Adjuvant Breast and Bowel Project trials in colon cancer. Semin Oncol 2001; 28 (Suppl 1): 9-13.

76. Cassidy J, Tabernero J, Twelves C, Brunet R, et al. XELOX (Capecitabine plus Oxaliplatin): Active First-Line Therapy for Patients with Metastatic Colorectal Cancer. Accepted J Clin Oncol 2004.

77. Shields A, Zalupski M, Marshall J, Meropol NJ. Treatment of Advanced Colorectal Carcinoma with Oxaliplatin and Capecitabine. A Phase II Trial. Cancer 2004; 100: 531-7.

78. Xeloda® US package insert.

79. Diasio, RB. Sorivudine and 5-fluorouracil: a clinically significant drug-drug interaction due to inhibition of dihydropyrimidine dehydrogenase. Br J Clin Pharmacol 1998, 46:1-4.

80. Martindale - The Complete Drug Reference - Monographs © 1982-2002. The Pharmaceutical Press.

81. Kolesar J, Johnson C, Freeberg B et al. Warfarin-5-FU interaction- a consecutive case series. Pharmacotherapy 1999; 19:1445-1449.

82. Willett CG, Boucher Y, Di Tomaso E, et al. Direct evidence that the VEGF-specific antibody bevacizumab has antivascular effects in human rectal cancer. Nat Med. 2004; 10: 145-7.

83. Kuenen BC, Levi M, Meijers JC, Kakkar AK, van Hinsbergh VW, Kostense PJ, Pinedo HM, Hoekman K. Analysis of coagulation cascade and endothelial cell activation during inhibition of vascular endothelial growth factor/vascular endothelial growth factor receptor pathway in cancer patients. Arterioscler Thromb Vasc Biol. 2002; 22:1500-5

84. Carmeliet P, Jain RK.Angiogenesis in cancer and other diseases. Nature 2000; 14; 407: 249-57

85. Yuan F, Chen Y, Dellian M, Safabakhsh N, Ferrara N, Jain RK. Time-dependent vascular regression and permeability changes in established human tumour xenografts induced by an anti-vascular endothelial growth factor/vascular permeability factor antibody. Proc Natl Acad Sci U.S.A. 1996; 93: 14765-70.

86. Lee CG, Heijn M, di Tomaso E, Griffon-Etienne G, Ancukiewicz M, Koike C, et al. Anti-vascular endothelial growth factor treatment augments tumour radiation response under normoxic or hypoxic conditions. Cancer Res 2000; 60: 5565-70.

87. Marcus R, Peritz E. Gabriel KR. On closed testing procedures with spezial reference to ordered analysis of variance. Biometrika 1976; 63: 655-660.

88. Westfall PH, Tobias RD, Rom D, Wolfinger RD, Hochberg Y. (1999): Multiple Comparison and Multiple Testing Using the SAS® System, Cary, NC: SAS Institute Inc.

89. http://ctep.cancer.gov/reporting/ctc.html

90. Folkman J. Seminars in Medicine of the Beth Israel Hospital, Boston. Clinical applications of research on angiogenesis. N Engl J Med. 1995;333: 1757-63.

91. Cassidy J, Scheithauer W, McKendrick J, et al. Capecitabine (X) vs bolus 5-FU/leucovorin as adjuvant treatment for colon cancer (the X-Act Study): positive efficacy results of a phase III trial. J Clin Oncol 2004; 22 (suppl): 247s, abstr 3509.

92. Goldberg RM.Current approaches to first-line treatment of advanced colorectal cancer. Clinical Colorectal Cancer 2004; 4: Suppl. 1, S9-S15.

93. Gill S, Goldberg RM. First-line treatment strategies to improve survival in patients with advanced colorectal cancer. Drugs 2004; 64 (1): 27-44.

PART II: ETHICS AND GENERAL STUDY ADMINISTRATION

12. ETHICAL ASPECTS

12.1 Local Regulations/Declaration of Helsinki

The investigator will ensure that this study is conducted in full conformance with the principles of the "Declaration of Helsinki" or with the laws and regulations of the country in which the research is conducted, whichever affords the greater protection to the individual. The study must fully adhere to the principles outlined in "Guideline for Good Clinical Practice" ICH Tripartite Guideline (January 1997) or with local law if it affords greater protection to the patient. For studies conducted in the USA or under US IND, the investigator will additionally ensure that the basic principles of "Good Clinical Practice" as outlined in the current version of 21 CFR, subchapter D, part 312, "Responsibilities of Sponsors and investigators", part 50, "Protection of Human Subjects", and part 56, "Institutional Review Boards", are adhered to.

In other countries where "Guideline for Good Clinical Practice" exist Roche and the investigators will strictly ensure adherence to the stated provisions.

12.2 Informed Consent

It is the responsibility of the investigator, or a person designated by the investigator (if acceptable by local regulations), to obtain written informed consent from each patient participating in this study, after adequate explanation of the aims, methods, anticipated benefits, and potential hazards of the study. For patients not qualified or incapable of giving legal consent, written consent must be obtained from the legally acceptable representative. In the case where both the patient and his/her legally acceptable representative are unable to read, an impartial witness should be present during the entire informed consent discussion. After the patient and representative have orally consented to participation in the trial, the witness' signature on the form will attest that the information in the consent form was accurately explained and understood. The investigator or designee must also explain that the patients are completely free to refuse to enter the study or to withdraw from it at any time, for any reason. The Case Report Forms for this study contain a section for documenting informed patient consent, and this must be completed appropriately. If new safety information results in significant changes in the risk/benefit assessment, the consent form should be reviewed and updated if necessary. All patients (including those already being treated) should be informed of the new information, given a copy of the revised form and give their consent to continue in the study.

12.3 Independent Ethics Committees/Institutional Review Board

Non-US sites: This protocol and any accompanying material provided to the patient (such as patient information sheets or descriptions of the study used to obtain informed consent) as well as any advertising or compensation given to the patient, will be submitted by the investigator to an Independent Ethics Committee. Approval from the committee must be obtained before starting the study, and should be documented in a

letter to the investigator specifying the date on which the committee met and granted the approval.

Any modifications made to the protocol after receipt of the Independent Ethics Committee approval must also be submitted by the investigator to the Committee in accordance with local procedures and regulatory requirements.

When no local review board exists, the investigator is expected to submit the protocol to a regional committee. If no regional committee exists, Roche will assist the investigator in submitting the protocol to the European Ethics Review Committee.

The *European Clinical Trials Directive* will be followed where applicable.

US sites: It is the understanding of the sponsor that this protocol (and any modifications) as well as appropriate consent procedures, will be reviewed and approved by an Institutional Review Board. This board must operate in accordance with the current US Federal Regulations. A letter or certificate of approval will be sent by the investigator to the sponsor prior to initiation of the study, and also whenever subsequent modifications to the protocol are made.

13. CONDITIONS FOR MODIFYING THE PROTOCOL

Protocol modifications to ongoing studies must be made only after consultation between an appropriate representative of the sponsor and the investigator. Protocol modifications must be prepared by a representative of the sponsor and initially reviewed and approved by the Clinical Science Leader and Biostatistician.

All protocol modifications must be submitted to the appropriate Independent Ethics Committee or Institutional Review Board for information and approval in accordance with local requirements, and to Regulatory Agencies if required. Approval must be awaited before any changes can be implemented, except for changes necessary to eliminate an immediate hazard to trial patients, or when the change(s) involves only logistical or administrative aspects of the trial [e.g. change in monitor(s), change of telephone number(s)].

14. CONDITIONS FOR TERMINATING THE STUDY

Both the sponsor and the investigator reserve the right to terminate the study at any time. Should this be necessary, both parties will arrange the procedures on an individual study basis after review and consultation. In terminating the study, Roche and the investigator will assure that adequate consideration is given to the protection of the patient's interests.

15. STUDY DOCUMENTATION, CRFS AND RECORD KEEPING

15.1 Investigator's Files / Retention of Documents

The investigator must maintain adequate and accurate records to enable the conduct of the study to be fully documented and the study data to be subsequently verified. These documents should be classified into two different separate categories (1) Investigator's Study File, and (2) patient clinical source documents.

The Investigator's Study File will contain the protocol/amendments, Case Report and Query Forms, Independent Ethics Committee/Institutional Review Board and governmental approval with correspondence, sample informed consent, drug records, staff curriculum vitae and authorization forms and other appropriate documents/correspondence etc.

Subject clinical source documents (usually defined by the project in advance to record key efficacy/safety parameters independent of the CRFs) would include patient hospital/clinic records, physician's and nurse's notes, appointment book, original laboratory reports, ECG, EEG, X-ray, pathology and special assessment reports, signed informed consent forms, consultant letters, and subject screening and enrollment logs. The investigator must keep these two categories of documents on file for at least 15 years after completion or discontinuation of the study. After that period of time the documents may be destroyed, subject to local regulations.

Should the investigator wish to assign the study records to another party or move them to another location, Roche must be notified in advance.

If the investigator can not guarantee this archiving requirement at the investigational site for any or all of the documents, special arrangements must be made between the investigator and Roche to store these in a sealed container(s) outside of the site so that they can be returned sealed to the investigator in case of a regulatory audit. Where source documents are required for the continued care of the patient, appropriate copies should be made for storing outside of the site.

15.2 Source Documents and Background Data

The investigator shall supply the sponsor on request with any required background data from the study documentation or clinic records. This is particularly important when Case Report Forms are illegible or when errors in data transcription are suspected. In case of special problems and/or governmental queries or requests for audit inspections, it is also necessary to have access to the complete study records, provided that patient confidentiality is protected.

15.3 Audits and Inspections

The investigator should understand that source documents for this trial should be made available to appropriately qualified personnel from the Roche Research Quality Assurance Unit or its designees, or to health authority inspectors after appropriate notification. The verification of the Case Report Form data must be by direct inspection of source documents.

15.4 Case Report Forms

For each patient enrolled, a Case Report Form must be completed and signed by the principal investigator or authorized delegate from the study staff. This also applies to records for those patients who fail to complete the study (even during a pre-randomization screening period if a Case Report Form was initiated). If a patient withdraws from the study, the reason must be noted on the Case Report Form. If a patient is withdrawn from the study because of a treatment-limiting adverse event, thorough efforts should be made to clearly document the outcome.

All forms should be typed or filled out using indelible ink, and must be legible. Errors should be crossed out but not obliterated, the correction inserted, and the change initialed and dated by the investigator or his/her authorized delegate. The investigator should ensure the accuracy, completeness, legibility, and timeliness of the data reported to the sponsor in the CRFs and in all required reports.

16. MONITORING THE STUDY

It is understood that the responsible Roche monitor (or designee) will contact and visit the Investigator regularly and will be allowed, on request, to inspect the various records of the trial (Case Report Forms and other pertinent data) provided that patient confidentiality is maintained in accord with local requirements.

It will be the monitor's responsibility to inspect the Case Report Forms at regular intervals throughout the study, to verify the adherence to the protocol and the completeness, consistency and accuracy of the data being entered on them. The monitor should have access to laboratory test reports and other patient records needed to verify the entries on the Case Report Form. The Investigator (or his/her deputy) agrees to cooperate with the monitor to ensure that any problems detected in the course of these monitoring visits are resolved.

17. CONFIDENTIALITY OF TRIAL DOCUMENTS AND SUBJECT RECORDS

The investigator must assure that patients' anonymity will be maintained and that their identities are protected from unauthorized parties. On CRFs or other documents submitted to the sponsor, patients should not be identified by their names, but by an identification code. The investigator should keep a patient enrollment log showing codes, names and addresses. The investigator should maintain documents not for submission to Roche, e.g., patients' written consent forms, in strict confidence.

18. PUBLICATION OF DATA AND PROTECTION OF TRADE SECRETS

In accord with standard editorial and ethical practice, Roche will generally support publication of multicenter trials only in their entirety and not as individual center data. A Final Study Report (FSR) will be prepared by Roche and provided to the two Lead Investigators for the study (the principal investigator and the co-principal investigator). Roche will make reasonable efforts to provide this report no more than 6 months following the final analysis of the primary endpoint of the study. The Lead Investigators will, in addition to the FSR, have full access to the complete trial data via Roche, and they will be expected to publish the results of the trial in a timely manner. Selection of co-authors, including Roche personnel where appropriate, will be by mutual agreement between Roche and the Lead Investigators. Roche has the right to review and comment on all publications and presentations of study data. This allows the sponsor to protect proprietary information and to provide comments based on information from other studies that may not yet be available to the investigators. Abstracts or manuscripts that are intended to be submitted for publication should be sent to Roche for review 45 days prior to planned final submission by the Lead Investigators.

Appendix 1 Adverse Events Categories for Determining Relationship to Test Drug

PROBABLE (must have first three)

This category applies to those adverse events which are considered, with a high degree of certainty, to be related to the test drug. An adverse event may be considered probable, if:

1. It follows a reasonable temporal sequence from administration of the drug.
2. It cannot be reasonably explained by the known characteristics of the patients clinical state, environmental or toxic factors, or other modes of therapy administered to the patient.
3. It disappears or decreases on cessation or reduction in dose. (There are important exceptions when an adverse event does not disappear upon discontinuation of the drug, yet drug-relatedness clearly exists; e.g., (1) bone marrow depression, (2) tardive dyskinesias.)
4. It follows a known pattern of response to the suspected drug.
5. It reappears upon rechallenge.

POSSIBLE (must have first two)

This category applies to those adverse events in which the connection with the test drug administration appears unlikely but cannot be ruled out with certainty. An adverse event may be considered possible if, or when:

1. It follows a reasonable temporal sequence from administration of the drug.
2. It may have been produced by the patients clinical state, environmental or toxic factors, or other modes of therapy administered to the patient.
3. It follows a known pattern of response to the suspected drug.

REMOTE (must have first two)

In general, this category is applicable to an adverse event which meets the following criteria:

1. It does not follow a reasonable temporal sequence from administration of the drug.
2. It may readily have been produced by the patients clinical state, environmental or toxic factors, or other modes of therapy administered to the patient.
3. It does not follow a known pattern of response to the suspected drug.
4. It does not reappear or worsen when the drug is readministered.

UNRELATED

This category is applicable to those adverse events which are judged to be clearly and incontrovertibly due only to extraneous causes (disease, environment, etc.) and do not meet the criteria for drug relationship listed under remote, possible, or probable.

Appendix 1 Adverse Events Categories for Determining Relationship to Test Drug (Cont.)

	Probable	Possible	Remote	Unrelated
Clearly due to extraneous causes	–	–	–	+
Reasonable temporal association with drug administration	+	+	–	–
May be produced by patient clinical state, etc.	–	+	+	+
Known response pattern to suspected drug	+	+	–	–
Disappears or decreases on cessation or reduction in dose	+	–	–	–
Reappears on rechallenge	+	–	–	–

Appendix 2 Serious Adverse Events (SAEs) Reporting Procedure

SAEs and pregnancies occurring during this study, or which come to the attention of the investigator during the protocol-defined follow-up period (see Sections 7.2.3, 7.2.4, 7.2.5), should be reported immediately to the sponsor (SAE responsible person). The investigator will complete one SAE reporting form for each SAE experienced by the patient.

When appropriate, this will have to be completed with the available EudraCT/sponsor's trial code number (if not pre-filled on the available forms).

The SAE page will be sent with the SAE Fax Cover sheet as described below:

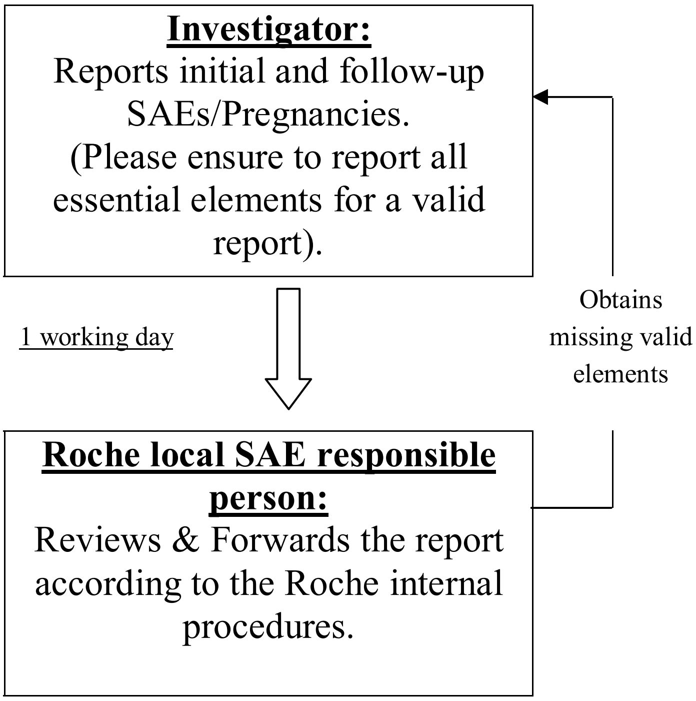

The initial report (containing the minimum reporting criteria) will be followed by detailed descriptions on the SAE form which will be based on hospital case reports, autopsy reports and other documents and applicable in further follow up reports.

The follow up information must be assessed and recorded on the SAE form and should also be faxed within one working day, containing at least the protocol number, the CRF number, event term and onset date.

Appendix 2 Serious Adverse Events (SAEs) Reporting Procedure (Cont.)

The essential elements of information required by Roche to validate SAE reports are:

Roche required minimal criteria for SAE reporting	*EU required minimal criteria*
Reporter / Investigators details<br>Trial number<br>Randomisation/Medication number<br>CRF number<br>Adverse Event<br>Suspected drug<br>Causality to the study medication<br>Reason why considered serious<br>Roche Received date<br>(to be completed by Roche Personnel)	*Suspect drug*<br>*Randomisation/Medication number*<br>*Reason for Serious and unexpected*<br>*Identifiable reporting source*

All missing elements will have to be sent to the sponsor within 1 working day of knowledge of the information.

It is the responsibility of the sponsor to contact the investigator in case of questions or if clarifications are needed. In the event of an "urgent" query from the sponsor, the investigator is expected to respond within one working day. If query is not considered urgent, the investigator has 10 working days to respond and resolve the query.

The SAE reporting form is the only accepted document for any relevant information (i.e. lab result sheets, ECGs and other source documents are not accepted but relevant results should be transcribed to the SAE reporting form). If necessary, source data should only be sent by request to the F. Hoffmann-La Roche monitor.

Initial pregnancy reporting will be performed on the SAE form. Further follow up data will be provided on the Clinical Trial Pregnancy Reporting Form.

Roche local SAE responsible person

(to be entered by the person for the specific country)

Name:

Telephone:

Fax:

Back-up person:

Appendix 3 ICH Guidelines for Clinical Safety Data Management, Definitions and Standards for Expedited Reporting, Topic E2

A serious adverse event is any experience that suggests a significant hazard, contraindication, side effect or precaution. It is any Adverse Event that at any dose fulfills at least one of the following criteria:

- is fatal; (results in death)
- (NOTE: death is an outcome, not an event)
- is Life-Threatening
- (NOTE: the term "Life-Threatening" refers to an event in which the patient was at immediate risk of death at the time of the event; it does not refer to an event which could hypothetically have caused a death had it been more severe).
- required in-patient hospitalization or prolongation of existing hospitalization;
- results in persistent or significant disability/incapacity;
- is a congenital anomaly/birth defect;
- is medically significant or requires intervention to prevent one or other of the outcomes listed above.

It is necessary to determine the causality, the severity (or seriousness) and the expectedness of the SAE (Serious Adverse Event).

Causality (possible cause of SAE), initially assessed by the investigator, can be one of the following 5 possibilities:

- Pre-existing / underlying disease,
- Study treatment,
- Other specific treatment (concomitant or previous),
- Protocol related procedure,
- Other (to be specified e.g. accident, new or intercurrent disease).

Severity is a measure of intensity. A Severe Adverse Event is not necessarily serious. For example, nausea of several hours' duration may be rated as severe, but may not be clinically serious.

The expectedness of an SAE is to be judged as expected or unexpected. An unexpected SAE is one of which the nature (or severity) is not consistent with the applicable product information (which can be found in the IB or in the SPC) and which is considered as adding significant information on the specificity of an expected adverse reaction.

Medical and scientific judgment should be exercised in deciding whether expedited reporting to the sponsor is appropriate in other situations, such as important medical events that may not be immediately life-threatening or result in death or hospitalisation but may jeopardise the patient or may require intervention to prevent one of the outcomes

Appendix 3 ICH Guidelines for Clinical Safety Data Management, Definitions and Standards for Expedited Reporting, Topic E2 (Cont.)

listed in the definitions above. These situations should also usually be considered serious. Examples of such events are intensive treatment in an emergency room or at home for allergic bronchospasm; blood dyscrasias or convulsions that do not result in hospitalisation; or development of drug dependency or drug abuse.

SAEs occurring after the patient signed the informed consent but before study drug exposure (e.g. related to the study related procedures) should also be reported according to the procedure described in Appendix 2.

F. Hoffmann - La Roche headquarters medical contact during office hours in Clinical Science Department (Central European time):

Özlem Anak, MD
PDM2
Bldg. 015/01.062
Grenzacherstrasse 124
CH-4070 BASEL, SWITZERLAND
Tel.: +41 61 688 42 27

Gregory Szelagowicz, MD
PDM2
Bldg. 015/01.062
Grenzacherstrasse 124
CH-4070 BASEL, SWITZERLAND
Tel.: +41 61 688 70 54

FAX number: + 41 61 688 98 99

Appendix 4 EU clinical Directives for SARs (Serious Adverse Reactions) management. Definitions and standard for expedited reporting

SUSARs;

Suspected Unexpected Serious Adverse Reactions (SUSARs) are suspected adverse reactions related to Investigational Medicinal Products (IMP) and/or comparator(s) (including the placebos), occurring in clinical trials, and are both unexpected and serious.

SUSARs associated with an IMP that does not hold a marketing authorisation and any other SUSARs associated with the IMP, in any Member State (MS) of the European Economic Area, are subject to expedited reporting to Competent Authorities and ethics committees/Institutional Review Board of the concerned MS, according to the EU-CTD guidelines, as soon as the sponsor becomes aware of them. This includes SUSARs which:

- Occur in another trial conducted by the same sponsor either in the European Community or in non-European Community countries
- Are identified by spontaneous reports or a publication
- Are transmitted to the sponsor by another regulatory authority

Other safety issues requiring expedited reporting:

Safety issues that might materially alter the current benefit-risk assessment of an IMP or that would be sufficient to consider changes in the IMP administration or in the overall conduct of the trial, also qualify for expedited reporting, for instance:

- Single case reports of an expected serious adverse reaction with an unexpected outcome (e.g. a fatal outcome)
- An increase in the rate and occurrence of an expected serious adverse reaction which is judged to be clinically important
- Post-study SUSARs that occur after the patient has completed a clinical trial and are reported by the investigator to the sponsor
- New events relating to the conduct of the trial or the development of the IMP likely to affect the safety of the subjects, such as:
    - An SAE which could be associated with the trial procedure and which could modify the conduct of the trial
    - A significant hazard to the patient population such as lack of efficacy of an IMP used for the treatment of a life-threatening disease
    - A major safety finding from a newly completed animal study (such as carcinogenicity)

Appendix 4 EU clinical Directives for SARs (Serious Adverse Reactions) management. Definitions and standard for expedited reporting (Cont.)

Where the IMP is authorised in a MS and the sponsor is the marketing authorisation holder, the reporting of SUSARs should take into account national requirements intended to manage duplication of reports in the context of Directive 2001/83/EC, Regulation 2309/93/EC and the: "Detailed guidance on the European database of Suspected Unexpected Serious Adverse Reactions (Eudravigilance – Clinical Trial Module)".

Expedited reporting is usually not required in the following instances:

- Reactions which are serious but expected
- Non-serious adverse reactions, whether they are expected or not
- Events considered unrelated to IMP

Fatal and life threatening SUSARs: Sponsor (F. Hoffman-La Roche) will report SUSARs to the competent authorities and the EC as soon as possible an not later than 7 calendar days. This reporting is based on receipt from the investigator site of the minimum criteria for expedited reporting (as defined in Appendix 2 and Appendix 3). Additional information should be available as soon as possible and reported within an additional 8 calendar days.

In each case, relevant follow-up information should be sought and a report completed as soon as possible. It should be communicated to the competent authority and the EC in the concerned Member States within an additional eight calendar days.

All other SUSARs and safety issues (requiring expedited reporting) will be reported to the competent authority and EC in the concerned member states as soon as possible and no later than 15 calendar days after F. Hoffmann-La Roche has first knowledge of the minimum criteria for expedited reporting. Further relevant follow-up information should be made available as soon as possible.

The distribution to the EC, of the safety letter and CIOMS-I reporting form (with analysis of similar events where produced), will be performed within 15 calendar days from the Roche received date (in all relevant European Economic Area member states).

In case the EC only communicate via investigators, the investigator will forward the information within 7 calendar days from the Roche received date to their ethics committee.

Appendix 5 Classification of Colon/Rectal Cancer

AJCC/ UICC	T	N	M	Dukes	Mod. Astler-Coller
Stage 0	Tis	N0	M0	-	-
Stage I	T1	N0	M0	A	A
	T2	N0	M0	A	B1
Stage IIA	T3	N0	M0	B	B2
Stage IIB	T4	N0	M0	B	B3
Stage IIIA	T1-T2	N1	M0	C	C1
Stage IIIB	T3-T4	N1	M0	C	C2/C3
Stage IIIC	Any T	N2	M0	C	C1/C2/C3
Stage IV	Any T	Any N	M1	-	D

PRIMARY TUMOUR	
TX	Primary tumour cannot be assessed
T0	No evidence of primary tumour
Tis	Carcinoma in situ; intraepithelial or invasion of lamina propria *
T1	Tumour invades submucosa
T2	Tumour invades muscularis propria
T3	Tumour invades through muscularis propria into subserosa or into non-peritonealized pericolic or perirectal tissues
T4	Tumour directly invades other organs or structures[2] and/or perforates visceral peritoneum **,***
*	Tis includes cancer cells confined within the glandular basement membrane (intraepithelial) or lamina propria (intramucosal) with no extension through muscularis mucosae into submucosa.
**	Direct invasion in T4 includes invasion of other segments of the colorectum by way of the serosa e.g invasion of sigmoid colon by a carcinoma of the cecum.
***	Tumour that is adherent to other organs or structures, macroscopically, is classified T4. However, if no tumour is present in the adhesion, microscopically, the classification should be pT3. The V and L substaging should be used to identify the presence or absence of vascular or lymphatic invasion.
**REGIONAL LYMPH NODES**	
NX	Regional lymph nodes cannot be assessed
N0	No regional lymph node metastasis
N1	Metastasis in 1 to 3 regional lymph nodes
N2	Metastasis in 4 or more regional lymph nodes
	A tumour nodule greater than 3 mm in diameter in the pericolorectal adipose tissue of a primary carcinoma without histologic evidence of a residual node in the nodule is classified in the pN category as a regional lymph node metastasis if the nodule has the form and smooth contour of a lymph node. If the nodule has an irregular contour, it should be classified in the T category and also coded as V1 (microscopic venous invasion) or as V2 (if it was grossly evident), because there is a strong likelihood that it represents venous invasion.
**DISTANT METASTASES**	
MX	Distant metastasis cannot be assessed
M0	No distant metastasis
M1	Distant metastasis
**RADIAL MARGINS AND RESIDUAL TUMOUR**	
R0	Complete resection, margins histologically negative, no residual tumour left after resection.
R1	Incomplete resection, margins histologically involved, microscopic tumour remains after resection of gross disease.
R2	Incomplete resection, margins involved or gross disease remains after resection.

Reference: Colon and rectum. In: American Joint Committee on Cancer: AJCC Cancer Staging Manual. 6th ed. / editors, Greene FL, Page, DL, Fleming ID, et al., Springer-Verlag, New York, Berlin, Heidelberg pp 113-119, and Maryland Cancer Registry classification.

Appendix 6 Calculation of Creatinine Clearance (Cockroft and Gault)

Cockroft-Gault Formula for *FEMALES:*

Creatinine clearance (mL/min) =

[(140 – age) x weight (in kg) x 0.85] / [72 x serum creatinine (in mg/dl)]

or

[(140 - age) x weight (in kg) x 0.85] / [0.81 x serum creatinine (in µmol/l)]

Cockroft-Gault Formula for *MALES:*

Creatinine clearance (mL/min) =

[(140 – age) x weight (in kg)] / [72 x serum creatinine (in mg/dl)]

or

[(140 - age) x weight (in kg)] / [0.81 x serum creatinine (in µmol/l)]

Ref: Cockcroft DW, Gault MH. Prediction of Creatinine Clearance from serum creatinine. Nephron 1976; 16:31.

Appendix 7 Nomogram for the Determination of the Body Surface Area

Nomogram for determination of body surface from height and mass[1]

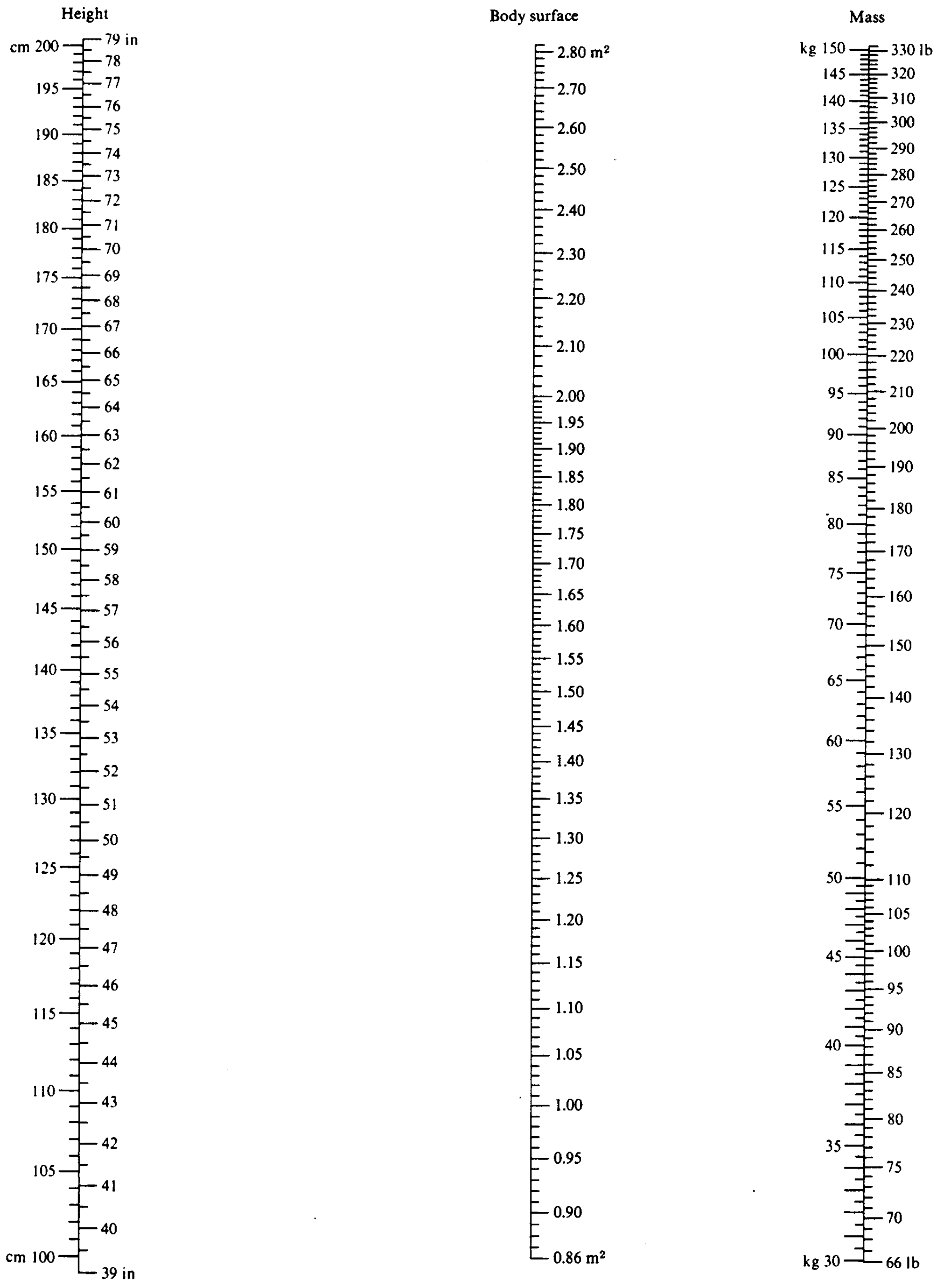

[1] From the formula of Du Bois and Du Bois, *Arch. intern. Med.*, **17**, 863 (1916): $S = M^{0.425} \times H^{0.725} \times 71.84$, or $\log S = \log M \times 0.425 + \log H \times 0.725 + 1.8564$ (S: body surface in cm², M: mass in kg, H: height in cm).

Ref: Lenter C, de. Geigy Scientific Tables, 8th ed. Basle, Switzerland: Ciba Geigy Ltd; 1981

Appendix 8 Capecitabine Dose Calculation According to Surface Area

**100% Dose Level**<br>**= twice daily 1000 mg/m²**		Number of tablets to be taken in the			
		Morning		Evening	
Surface Area (m^2)	Total Dose per Administration (mg)*	150 mg	500 mg	150 mg	500 mg
≤ 1.22	1150	1	2	1	2
1.23 – 1.40	1300	2	2	2	2
1.41 – 1.57	1500	–	3	–	3
1.58 – 1.72	1650	1	3	1	3
1.73 – 1.90	1800	2	3	2	3
1.91 – 2.07	2000	–	4	–	4
2.08 – 2.22	2150	1	4	1	4
≥ 2.23	2300	2	4	2	4

**75% Dose Level**<br>**= twice daily 750 mg/m²**		Number of tablets to be taken in the			
		Morning		Evening	
Surface Area (m^2)	Total Dose per Administration (mg)*	150 mg	500 mg	150 mg	500 mg
≤ 1.22	800	2	1	2	1
1.23 – 1.40	1000	-	2	-	2
1.41 – 1.57	1150	1	2	1	2
1.58 – 1.90	1300	2	2	2	2
1.91 – 2.07	1500	-	3	-	3
≥ 2.08	1650	1	3	1	3

**50% Dose Level**<br>**= twice daily 500 mg/m²**		Number of tablets to be taken in the			
		Morning		Evening	
Surface Area (m^2)	Total Dose per Administration (mg)*	150 mg	500 mg	150 mg	500 mg
≤ 1.22	500	-	1	-	1
1.23 – 1.40	650	1	1	1	1
1.41 – 1.90	800	2	1	2	1
1.91 – 2.07	1000	-	2	-	2
≥ 2.08	1150	1	2	1	2

* Total dose calculated on mg/m^2 for the median value, and is rounded to the nearest value which can be conveniently dosed equally in the morning and afternoon with 150 mg and 500 mg tablets.

Appendix 9 Deviations from treatment schedule

Note: In general, the term "lost treatment days" applies to capecitabine and bevacizumab only. For oxaliplatin, this refers to isolated neurotoxicity only. In contrast "extended rest periods" (treatment delay) refer to a postponement of the entire regimen.

Capecitabine

Normal Treatment:

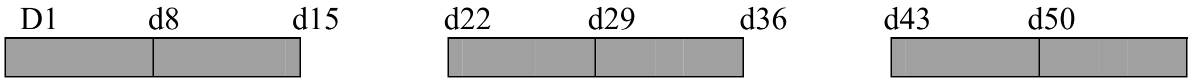

Interruptions During Treatment:
Interruptions are regarded as **lost treatment days** and the planned treatment schedule should be maintained.

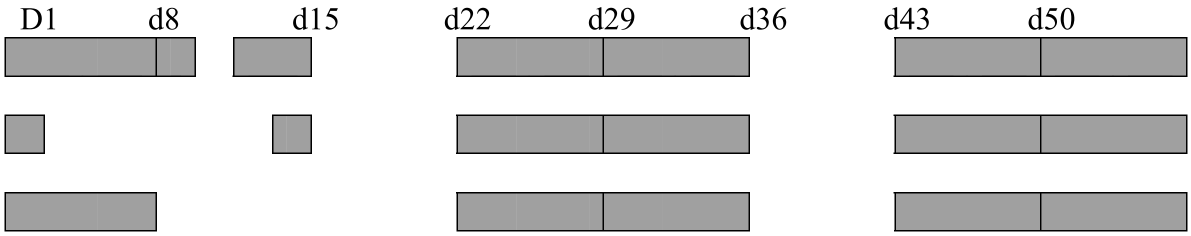

Extended Rest Periods:
If a rest period is extended due to toxicity, the "complete" cycle should be delayed i.e. given afterwards.

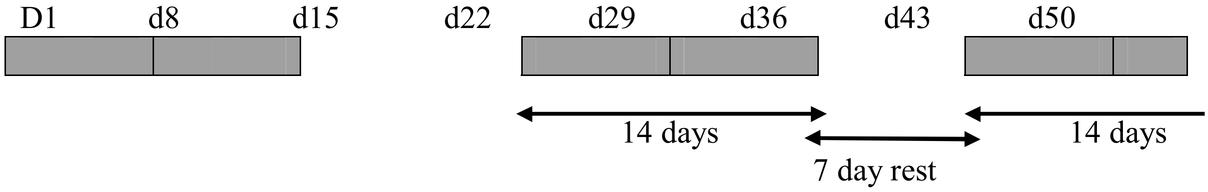

Appendix 9 Deviations from treatment schedule (Cont.)
Bevacizumab in combination with chemotherapy

Normal Treatment:

Bevacizumab + FOLFOX
D1 d8 d15 d22 d29 d36 d43 d50
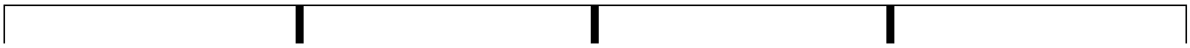

Bevacizumab + XELOX
D1 d8 d15 d22 d29 d36 d43 d50
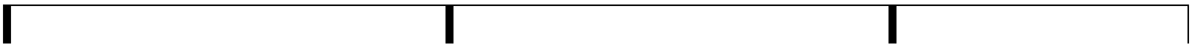

Interruptions During Treatment:
Interruptions are regarded as lost treatment days and the planned treatment schedule should be maintained.

Bevacizumab + FOLFOX
D1 d8 d15 d22 d29 d36 d43 d50
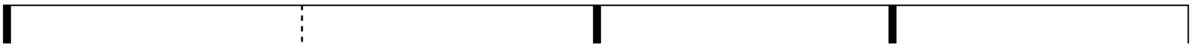

Bevacizumab + XELOX
D1 d8 d15 d22 d29 d36 d43 d50
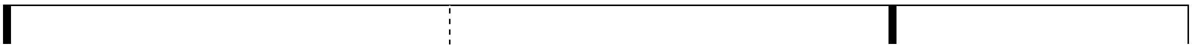

Extended Rest Periods:
If a rest period is extended due to toxicity, the "complete" cycle should be delayed i.e. given afterwards.

Bevacizumab + FOLFOX
D1 d8 d15 d22 d29 d36 d43 d50
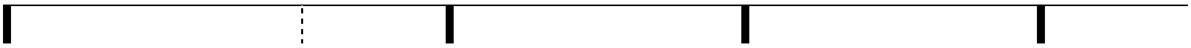

Bevacizumab + XELOX
D1 d8 d15 d22 d29 d36 d43 d50
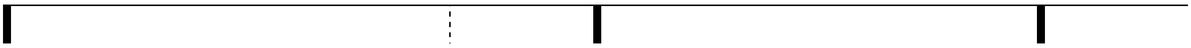

▌ ≅ administered bevacizumab
┆ ≅ planned administration

Appendix 10 Cardiovascular Disease

Congestive Heart Failure

New York Heart Association Criteria

Class	
I	*No limitation:* Ordinary physical activity does not cause undue fatigue, dyspnea, or palpitation.
II	*Slight limitation of physical activity:* Such patients are comfortable at rest. Ordinary physical activity results in fatigue, palpitations, dyspnea, or angina.
III	*Marked limitation of physical activity:* Although patients are comfortable at rest, less than ordinary physical activity will lead to symptoms.
IV	*Inability to carry on physical activity without discomfort:* Symptoms of congestive heart failure are present even at rest. With any physical activity, increased discomfort is experienced.

Source: Criteria Committee, New York Heart Association, Inc. Diseases of the heart and blood vessels. Nomenclature and criteria for diagnosis. 6th ed. Boston, Little, Brown and Co, 1964:114.

Peripheral vascular disease

Clinical Categories of Chronic Limb Ischemia

Grade	Category	Clinical Description	Objective Criteria
0	0	Asymptomatic—not hemodynamically significant	Normal treadmill/stress test
I	1	Mild claudication	Completes treadmill exercise (5 min at 2 mph on a 12% incline) AP after exercise <50 mm Hg but >25 mm Hg less than BP
	2	Moderate claudication	Between Categories 1 and 3
	3	Severe claudication	Cannot complete treadmill exercise and AP after exercise <50 mm Hg
II	4	Ischemic rest pain	Resting AP <40 mm Hg, flat or barely pulsatile ankle or metatarsal PVR; TP <30 mm Hg
	5	Minor tissue loss-nonhealing ulcer; focal gangrene with diffuse pedal ischemia	Resting AP <60 mm Hg, ankle metatarsal PVR flat or barely pulsatile; TP <40 mm Hg
III	6	Major tissue loss extending above TM level; functional foot no longer salvageable	Same as Category 5

AP=ankle pressure; BP=blood pressure; PVR=pulse volume recording; TP=toe pressure; TM=transmetatarsal.
Source: Pentecost MJ, Criqui MH, Dorros G, Goldstone J, Johnston KW, Martin EC, et al. Guidelines for peripheral percutaneous transluminal angioplasty of the abdominal aorta and lower extremity vessels. Circulation 1994;89:511–31.

Appendix 11 Monitoring of Proteinuria

Baseline Assessment of Proteinuria

Twenty-four hour urine collection and dipstick urinalysis will be used as the baseline assessment of proteinuria before randomisation.

Assessment of Proteinuria during Study

Assessment of proteinuria and adjustment of bevacizumab administration for proteinuria will occur according to the following guidelines:

- First occurrence of proteinuria:
  - 1+ (dipstick): Administer bevacizumab as scheduled, NO additional evaluation is required.
  - 2+ , 3+ and 4+ proteinuria (dipstick): Administer bevacizumab as scheduled and collect 24-hour urine to determine the total protein within 3 days prior to the next scheduled dose is required:
    - 24-hour proteinuria ≤ 2 g: Administer bevacizumab as scheduled.
    - 24-hour proteinuria > 2 g: Bevacizumab treatment should be withheld pending next 24 hour total protein.
      - Repeat 24-hour urine protein ≤ 2 g: Administer bevacizumab as schedule. 24-hour protein should be further monitored prior to each administration of bevacizumab until it has decreased to ≤ 1 g/24-hour.
      - Repeat 24-hour urine protein > 2 g: Bevacizumab dose should be withheld until 24-hour protein has decreased to ≤ 2 g. 24-hour protein should be further monitored prior to each administration of bevacizumab until it has decreased to ≤ 1 g/24-hour.
- Second and subsequent occurrence of ≥ 2+ proteinuria (dipstick):
  - 2+ (dipstick): Administer bevacizumab as scheduled, NO additional evaluation is required.
  - 3+ and 4+ proteinuria (dipstick): Administer bevacizumab as scheduled and collect 24-hour urine to determine the total protein within 3 days prior to the next scheduled dose is required:
    - 24-hour proteinuria ≤ 2 g: Administer bevacizumab as scheduled.
    - 24-hour proteinuria > 2 g: Bevacizumab treatment should be withheld pending next 24 hour total protein.

Appendix 11 Monitoring of Proteinuria (Cont.)

- Repeat 24-hour urine protein ≤ 2 g: Administer bevacizumab as schedule. 24-hour protein should be further monitored prior to each administration of bevacizumab until it has decreased to ≤ 1 g/24-hour.
- Repeat 24-hour urine protein > 2 g: Bevacizumab dose should be withheld until 24-hour protein has decreased to ≤ 2 g. 24-hour protein should be further monitored prior to each administration of bevacizumab until it has decreased to ≤ 1 g/24-hour.

- *Nephrotic syndrome* (Grade 4, CTCAE v 3.0): Discontinue bevacizumab treatment.

Algorithm for proteinuria (dipstick) 2+ and bevacizumab dose interruption:

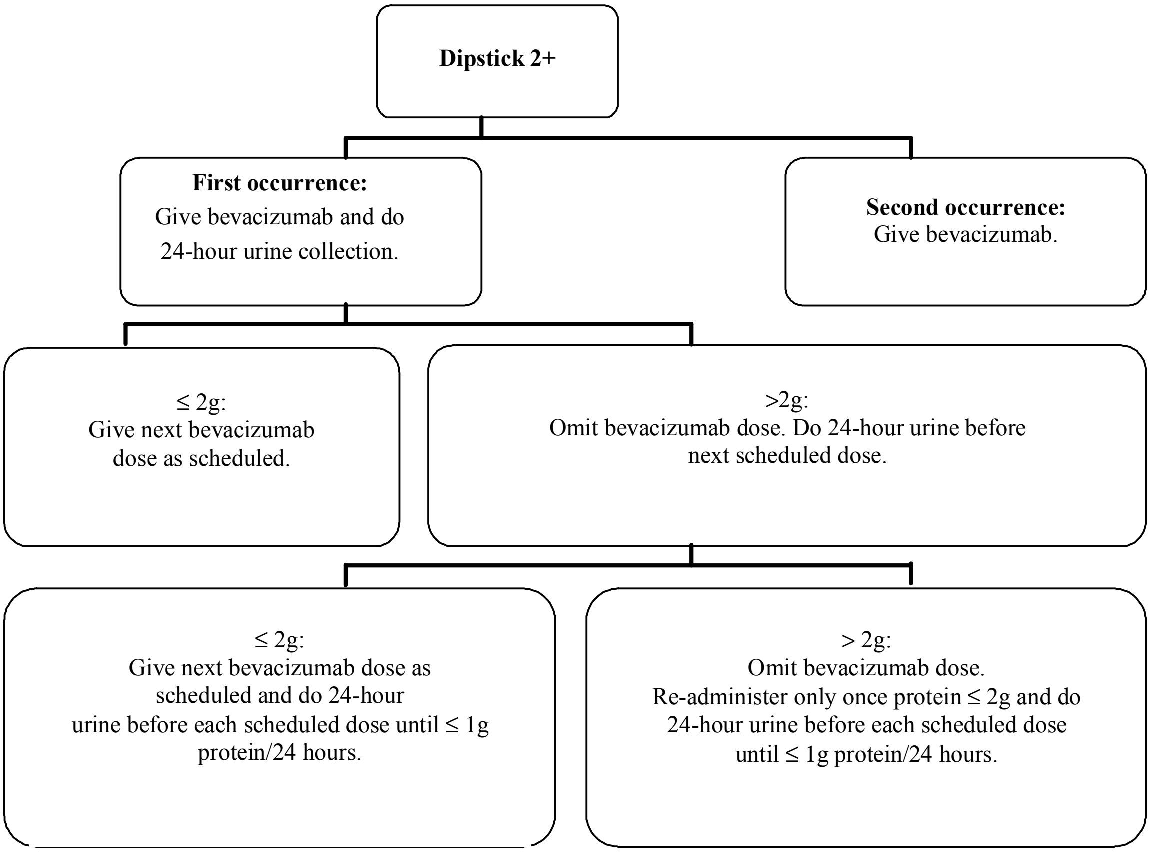

Appendix 11 Monitoring of Proteinuria (Cont.)

Algorithm for proteinuria (dipstick) ≥ 3+ and bevacizumab dose interruption:

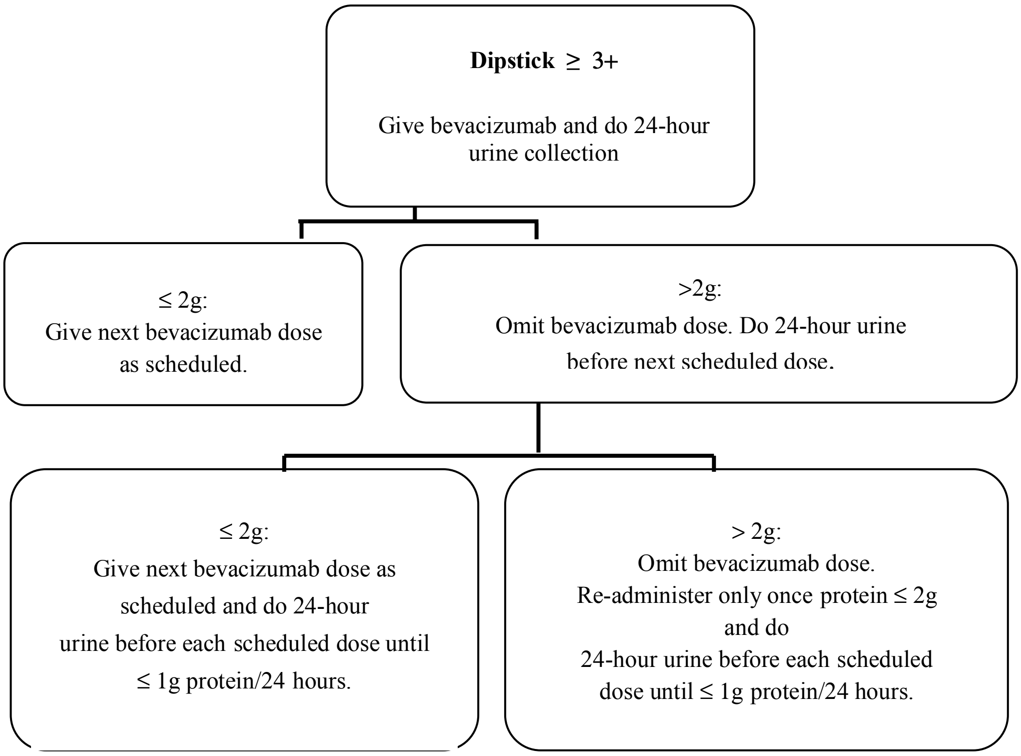

Additional Monitoring of Proteinuria during the Study

At the first onset of proteinuria of ≥ 2+ on dipstick during the study, patients should undergo additional monitoring of proteinuria in a 24-hour urine collection:

- Urine protein/creatinine ratio.

Status of Subjects with Proteinuria

Note: Patients with >1g proteinuria/24-hour at baseline are excluded from this study.

Patients who develop > 2g proteinuria/24-hour during the study will not receive additional doses of bevacizumab unless proteinuria improves to ≤ 2g/24-hour.

Patients who have ongoing proteinuria at the termination or early termination visit or who experience a new proteinuria event up to 6 months after the end of study treatment phase will be monitored for 24-hour urine collection every 3 months for up to 1 year or until total 24-hour urine protein improves to ≤ 1g.

Appendix 12 Anaphylaxis Precautions

Anaphylaxis

Anaphylaxis is defined as vascular collapse and shock (blood pressure <90 mm Hg that is unresponsive to IV fluids) believed to be allergic in origin, with or without antecedent respiratory distress and occurring within 30 minutes of initiation of bevacizumab infusion. Cutaneous manifestations include pruritus, urticaria, or angioedema.

Equipment Needed

Tourniquet
Oxygen
Epinephrine 1:1000 solution for IV or endotracheal injection
Antihistamines
Corticosteroids
IV infusion solutions, tubing, catheters, and tape

Procedures

In the event of a suspected anaphylactic reaction during infusion of study drug:

1. Stop the study drug infusion.
2. Apply a tourniquet proximal to the injection site to slow systemic absorption of bevacizumab. Do not obstruct arterial flow in the limb.
3. Maintain an adequate airway.
4. Administer antihistamines, epinephrine, or other medications as required by patient status and directed by the physician in charge.

Continue to observe the patient and document observations.

Appendix 13 ECOG Performance Status Scale

ECOG Scale		Approximate Karnofsky Score
0	Asymptomatic, normal activity	100%
1	Symptomatic but fully ambulatory	85%
2	Symptomatic, in bed less than 50% of time	65%
3	Symptomatic, in bed more than 50% of time, not bedridden	40%
4	100% bedridden	15%

Appendix 14 Human Anti-Human Antibodies to bevacizumab (HAHA)

In arms B and C, sample will be taken pre-bevacizumab on day 1 of cycle 1, after 1 year and after 1.5 years after randomization. In the case of a withdrawal of the patient from treatment samples have to be drawn 3 and 6 months after last administration.

To allow potential later investigation, a sample will also be collected in the event of a hypersensitivity reaction attributable to bevacizumab, occurring beyond the first administration.

Before the sample is collected ensure that the tubes for the blood and serum are correct and that their labels correspond to the patient and sample numbers in the CRF. Blood collection tube should be labelled with a WHITE label and the serum plastic storage tube should have a GREY label. 5 mL of blood will be drawn into a blood collection tube (WHITE labelled vacuum tube with no anticoagulant, to be provided by the site), at the sampling times mentioned above.

The sample should be processed within one hour of collection. Allow to clot at room temperature for approximately 30 minutes. Ensure the sample has fully clotted before centrifuging. Centrifuge the sample within one hour of collection. Centrifuge at approximately 1500g for 10 minutes. If the sample does not separate in this time and the clot has not fallen to the bottom of the tube, then re-centrifuge until the clot sinks to the bottom of the tube. Use a pipette to transfer the top layer of serum into a plastic storage tube (GREY label).

The serum samples should be stored in an upright position at -70°C in the polystyrene storage racks supplied by the sponsor. In case the samples have to be kept at -20°C, shipment to Roche central sample office (CSO) should be arranged within one month after the blood draw. The temperature of the freezer should be monitored and recorded.

Sample shipment for HAHA samples

Biological samples taken from all subjects are not known to be infectious. The samples from this study are classified as diagnostic specimens. For shipping purposes the samples should be classified as 'UN 3373 diagnostic specimens'.

Roche will supply the investigator with all materials and instructions required for shipment of samples. Samples should be shipped in the polystyrene racks provided by Roche.

The site will be provided with 'Biological Sample Transfer Forms' and 'Sample Proforma Invoice Forms' for use in organising shipment of samples to Roche. The Transfer Form must be completed by the study site at least 3 days before any shipment is planned and faxed to the nominated Courier Company. The appropriate Roche Central Sample Office (CSO) will provide instructions on how these forms should be completed, and where the samples should be shipped.

The completed Delivery Note for Biological samples should be included with each package in a sealed plastic envelope.

If you have any problems or queries regarding the collection, handling and shipping of the samples please contact your local monitor.

Appendix 15 Chemotherapy Convenience and Satisfaction Questionnaire (CCSQ)

CHEMOTHERAPY CONVENIENCE AND SATISFACTION QUESTIONNAIRE
ON THERAPY

Below is a list of statements that people receiving chemotherapy like yours have said are important. For each statement, please choose the reply that best fits your experience with receiving chemotherapy, and circle the number corresponding to your reply.

"Chemotherapy" means the drug(s) you receive to treat your cancer or tumor.

	Chemotherapy Experience - 1	Not at all	A little bit	Some-what	Quite a bit	Very Much
CS1	Chemotherapy treatment takes up my time.	0	1	2	3	4
CS2	My chemotherapy treatment takes up my family's time.	0	1	2	3	4
CS3	I worry about side effects from chemotherapy treatment.	0	1	2	3	4
CS4	My chemotherapy treatment causes me physical pain.	0	1	2	3	4
CS5	Receiving chemotherapy is inconvenient.	0	1	2	3	4
CS6	I worry that my chemotherapy will not be effective.	0	1	2	3	4
CS7	Chemotherapy treatment seems harmful to me.	0	1	2	3	4
CS8	My chemotherapy schedule is stressful to me.	0	1	2	3	4
CS9	My chemotherapy schedule is stressful to my family.	0	1	2	3	4
GP5	I am bothered by side effects of treatment.	0	1	2	3	4
	Please answer this last question about how you have felt this past week.					
GF7	I am content with the quality of my life right now.	0	1	2	3	4

Copyright 2002, FACIT, David Cella, Ph.D.
Version 6, 11-27-02

Appendix 15 Chemotherapy Convenience and Satisfaction Questionnaire (CCSQ) (Cont.)

CHEMOTHERAPY CONVENIENCE AND SATISFACTION QUESTIONNAIRE
ON THERAPY

Considering your experience with chemotherapy to date, please respond to the following questions.

	Chemotherapy Experience - 2	No, not at all	Yes, to some extent	Yes, for the most part	Yes, completely
CS 10	Are you satisfied with the current results of your chemotherapy?	0	1	2	3

		No	Maybe	Yes
CS 11	Would you recommend this chemotherapy to others with your illness?	0	1	2
CS 12	Would you choose this chemotherapy again?	0	1	2

		Poor	Fair	Good	Very Good	Excellent
CS 13	How would you rate this chemotherapy?	0	1	2	3	4

Copyright 2002, FACIT, David Cella, Ph.D.
Version 6, 11-27-02
