# Supplementary material for: Association of Bevacizumab Plus Oxaliplatin-Based Chemotherapy With Disease-Free Survival and Overall Survival in Patients With Stage II Colon Cancer: A Secondary Analysis of the AVANT Trial
Source: JAMA Netw Open. 2020 Oct 19;3(10):e2020425. doi: 10.1001/jamanetworkopen.2020.20425 (PMC7573695; doi:10.1001/jamanetworkopen.2020.20425)
Supplement: Supplement 2. — eTable 1. Univariate Analysis of Prognostic Factors for Disease-Free Survival and Overall Survival eTable 2. Multivariate Analysis of Prognostic Factors for Disease-Free Survival and Overall Survival eFigure 1. Distribution of Patients According to the Criteria Defining High-Risk Stage II Colon Cancer eFigure 2. Forest Plots for Disease-Free Survival and Overall Survival for FOLFOX4 vs FOLFOX4 with Bevacizumab and Disease-Free Survival and Overall Survival for FOLFOX4 vs XELOX with Bevacizumab [file jamanetwopen-e2020425-s002.pdf]

Supplemental Online Content

Chibaudel B, Henriques J, Rakez M, et al. Association of bevacizumab plus oxaliplatin-based chemotherapy with disease-free survival and overall survival in patients with stage II colon cancer: a secondary analysis of the AVANT Trial. *JAMA Netw Open*. 2020;3(10):e2020425. doi:10.1001/jamanetworkopen.2020.20425

eTable 1. Univariate Analysis of Prognostic Factors for Disease-Free Survival and Overall Survival

eTable 2. Multivariate Analysis of Prognostic Factors for Disease-Free Survival and Overall Survival

eFigure 1. Distribution of Patients According to the Criteria Defining High-Risk Stage II Colon Cancer

eFigure 2. Forest Plots for Disease-Free Survival and Overall Survival for FOLFOX4 vs FOLFOX4 with Bevacizumab and Disease-Free Survival and Overall Survival for FOLFOX4 vs XELOX with Bevacizumab

This supplemental material has been provided by the authors to give readers additional information about their work.

© 2020 Chibaudel B et al. *JAMA Network Open.*

eTable 1. Univariate Analysis of Prognostic Factors for Disease-Free Survival and Overall Survival

Variable		No. (event)	HR	95% CI	*P*
**Disease-free survival**					
**Arm**	A	192 (38)	1		
	B	194 (36)	0.94	0.59 to 1.48	.78
	C	187 (40)	1.07	0.69 to 1.67	.76
**Gender**	Male	325 (70)	1		
	Female	248 (44)	0.82	0.56 to 1.20	.31
**Age, year**	< 50	191 (26)	1		
	≥ 50	382 (88)	1.69	1.09 to 2.61	.02
	< 60	349 (56)	1		
	≥ 60	224 (58)	1.58	1.10 to 2.28	.01
	< 70	498 (85)	1		
	≥ 70	75 (29)	2.42	1.58 to 3.68	< .001
**Primary tumor**	Left/rectum	343 (71)	1		
	Right	211 (38)	0.83	0.56 to 1.23	.34
**Differentiation**	Well	109 (22)	1		
	Moderately	374 (74)	0.93	0.58 to 1.50	.77
	Poorly	67 (12)	0.76	0.37 to 1.53	.44
**ECOG PS**	0	464 (85)	1		
	1	101 (25)	1.61	1.03 to 2.51	.04
**T stage**	T3	362 (63)	1		
	T4	211 (51)	1.41	0.98 to 2.04	.07
**Perforation or obstruction**	No	428 (81)	1		
	Yes	145 (33)	1.23	0.82 to 1.85	.32
**Vascular or perineural invasion**	No	456 (90)	1		
	Yes	117 (24)	1.01	0.64 to 1.58	.97
**No. of examined nodes**	≥ 12	328 (55)	1		
	< 12	243 (58)	1.57	1.08 to 2.27	.02
**BMI**	< 30	501 (102)	1		
	≥ 30	72 (12)	0.82	0.45 to 1.49	.52
**Overall survival**					
**Arm**	A	192 (30)	1		
	B	194 (28)	0.92	0.55 to 1.55	.76
	C	187 (26)	0.85	0.50 to 1.44	.55
**Gender**	Male	325 (50)	1		
	Female	248 (34)	0.88	0.57 to 1.37	.58
**Age, year**	< 50	191 (16)	1		
	≥ 50	382 (68)	2.07	1.20 to 3.57	.009
	< 60	349 (36)	1		
	≥ 60	224 (48)	2.05	1.33 to 3.16	.001
	≤ 70	498 (58)	1		
	< 70	75 (26)	3.21	2.02 to 5.10	< .001

© 2020 Chibaudel B et al. *JAMA Network Open.*

**Primary tumor**	Left/rectum	343 (52)	1		
	Right	211 (28)	0.84	0.53 to 1.32	.44
**Differentiation**	Well	109 (15)	1		
	Moderately	374 (58)	1.08	0.79 to 2.37	.26
	Poorly	67 (8)	0.74	0.31 to 1.75	.49
**ECOG PS**	0	464 (64)	1		
	1	101 (16)	1.37	0.79 to 2.37	.26
**T stage**	T3	362 (46)	1		
	T4	211 (38)	1.45	0.95 to 2.23	.09
**Perforation or obstruction**	No	428 (59)	1		
	Yes	145 (25)	1.25	0.79 to 2.00	.34
**Vascular or perineural invasion**	No	456 (67)	1		
	Yes	117 (17)	0.92	0.54 to 1.57	.77
**No. of examined nodes**	≥ 12	328 (39)	1		
	< 12	243 (45)	1.75	1.14 to 2.69	.01
**BMI**	< 30	501 (72)	1		
	≥ 30	72 (12)	1.25	0.68 to 2.31	.47

Abbreviations: HR, hazard ratio; CI, confidence interval; ECOG, Eastern Cooperative Oncology Group; PS, performance status; BMI, body mass index

© 2020 Chibaudel B et al. *JAMA Network Open.*

eTable 2. Multivariate Analysis of Prognostic Factors for Disease-Free Survival and Overall Survival

**Variable**		**No. (event)**	**HR**	**95% CI**	***P***
**Disease-free survival**					
		571[ϕ] (113)			
**Arm**	A		1		
	B		0.91	0.58 to 1.45	.70
	C		1.08	0.69 to 1.68	.74
**Age, year**	< 50		1		
	≥ 50		1.56	1.00 to 2.45	.05
**T stage**	T3		1		
	T4		1.52	1.04 to 2.21	.03
**No. of examined nodes**	≥ 12		1		
	< 12		1.57	1.07 to 2.29	.02
**Overall survival**					
		571[ϕ] (84)			
**Arm**	A		1		
	B		0.92	0.55 to 1.54	.75
	C		0.85	0.50 to 1.44	.55
**Age, year**	< 50		1		
	≥ 50		1.83	1.05 to 3.17	.03
**T stage**	T3		1		
	T4		1.57	1.01 to 2.44	.04
**No. of examined nodes**	≥ 12		1		
	< 12		1.73	1.11 to 2.69	.02

ϕ: Two patients had missing data for the number of examined nodes

Abbreviations: HR, hazard ratio; CI, confidence interval

© 2020 Chibaudel B et al. *JAMA Network Open.*

eFigure 1. Distribution of Patients According to the Criteria Defining High-Risk Stage II Colon Cancer

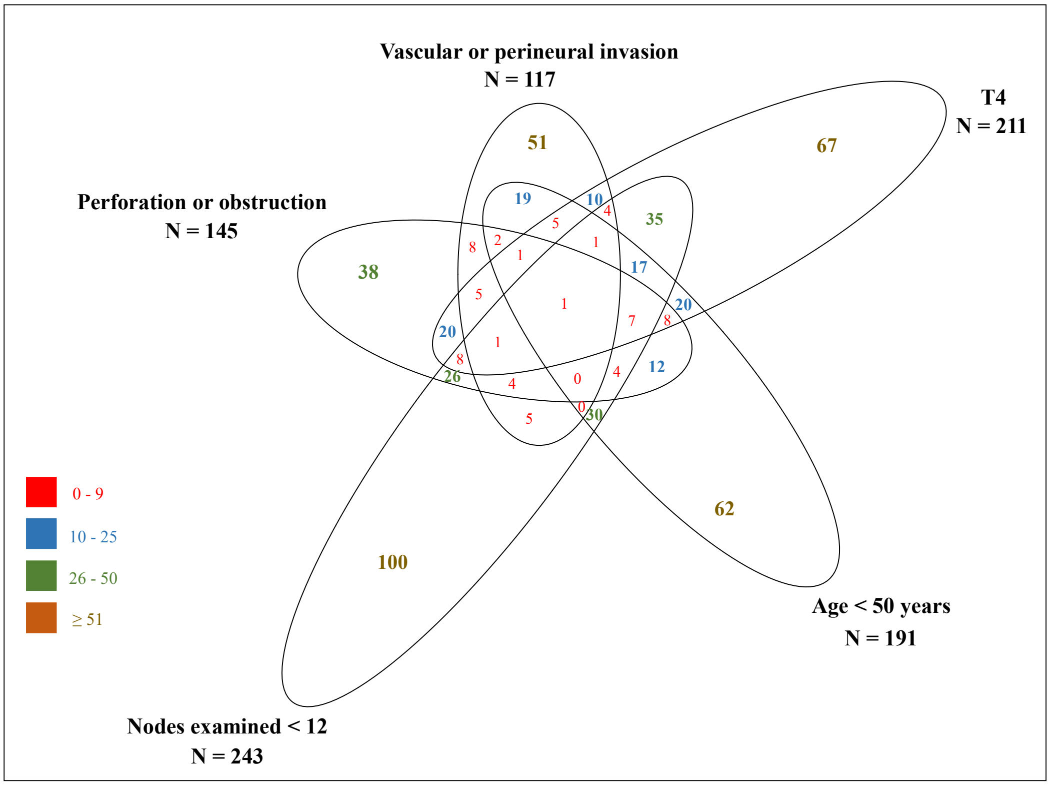

© 2020 Chibaudel B et al. *JAMA Network Open.*

eFigure 2. Forest Plots for Disease-Free Survival (A) and Overall Survival (B) for FOLFOX4 vs FOLFOX4 with Bevacizumab and Disease-Free Survival (C) and Overall Survival (D) for FOLFOX4 vs XELOX with Bevacizumab

A

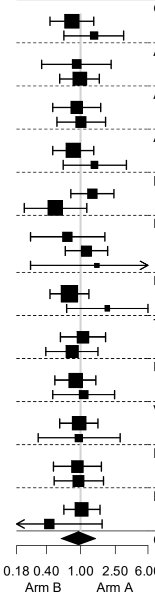

Covariate	N	Event	HR	LCI	UCI	P-value*
Gender						.25
male	223	47	0.79	0.44	1.42	
female	163	27	1.42	0.64	3.16	
Age (cutoff = 50 yrs)						.89
< 50	132	18	0.89	0.35	2.25	
50+	254	56	0.97	0.57	1.63	
Age (cutoff = 60 yrs)						.80
< 60	230	37	0.90	0.47	1.71	
60+	156	37	1.00	0.53	1.92	
Age (cutoff = 70 yrs)						.24
< 70	327	52	0.82	0.47	1.41	
70+	59	22	1.44	0.62	3.35	
Primary tumor						.06
left/rectum	236	47	1.35	0.76	2.40	
right	137	24	0.50	0.22	1.18	
Differentiation						.38
well	73	17	0.70	0.26	1.89	
moderately	253	48	1.16	0.66	2.05	
poorly	44	5	1.54	0.26	9.23	
ECOG PS						.01
0	321	58	0.74	0.44	1.24	
1	62	15	2.03	0.69	5.95	
T-stage						.52
T3	243	42	1.06	0.58	1.93	
T4	143	32	0.79	0.39	1.58	
Perforation or obstruction						.67
no	284	51	0.87	0.50	1.50	
yes	102	23	1.08	0.47	2.46	
Vascular or perineural invasion						.96
no	309	61	0.95	0.57	1.57	
yes	77	13	0.94	0.32	2.83	
Number of examined nodes						.91
< 12	165	38	0.91	0.48	1.72	
12+	220	35	0.94	0.49	1.83	
BMI						.35
< 30	338	65	1.02	0.63	1.66	
30+	48	9	0.43	0.10	1.76	
**Overall**	**386**	**74**	**0.94**	**0.59**	**1.48**	

B

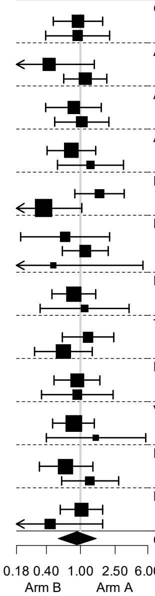

Covariate	N	Event	HR	LCI	UCI	P-value*
Gender						.98
male	223	36	0.93	0.48	1.80	
female	163	22	0.92	0.39	2.16	
Age (cutoff = 50 yrs)						.16
< 50	132	12	0.43	0.13	1.43	
50+	254	46	1.13	0.63	2.02	
Age (cutoff = 60 yrs)						.65
< 60	230	28	0.83	0.39	1.74	
60+	156	30	1.03	0.50	2.11	
Age (cutoff = 70 yrs)						.32
< 70	327	38	0.78	0.41	1.48	
70+	59	20	1.30	0.54	3.14	
Primary tumor						.01
left/rectum	236	37	1.65	0.85	3.20	
right	137	19	0.37	0.13	1.02	
Differentiation						.41
well	73	12	0.66	0.20	2.18	
moderately	253	41	1.13	0.61	2.09	
poorly	44	3	0.48	0.04	5.30	
ECOG PS						.74
0	321	46	0.83	0.46	1.48	
1	62	11	1.10	0.34	3.61	
T-stage						.20
T3	243	32	1.22	0.61	2.43	
T4	143	26	0.63	0.29	1.37	
Perforation or obstruction						.99
no	284	41	0.91	0.49	1.68	
yes	102	17	0.91	0.35	2.37	
Vascular or perineural invasion						.43
no	309	49	0.83	0.47	1.45	
yes	77	9	1.5	0.40	5.62	
Number of examined nodes			1.50			.24
< 12	165	31	0.67	0.33	1.38	
12+	220	27	1.28	0.60	2.73	
BMI						.32
< 30	338	49	1.02	0.58	1.79	
30+	48	9	0.44	0.11	1.80	
**Overall**	**386**	**58**	**0.91**	**0.55**	**1.53**	

C

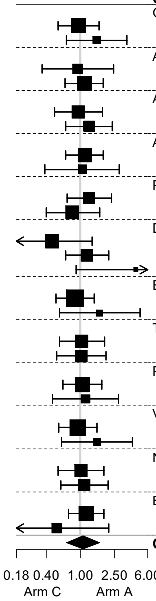

Covariate	N	Event	HR	LCI	UCI	P-value*
Gender						.34
male	226	52	0.95	0.55	1.64	
female	153	26	1.56	0.69	3.50	
Age (cutoff = 50 yrs)						.74
< 50	121	17	0.93	0.36	2.42	
50+	258	61	1.11	0.67	1.84	
Age (cutoff = 60 yrs)						.51
< 60	231	38	0.95	0.50	1.80	
60+	148	40	1.27	0.68	2.36	
Age (cutoff = 70 yrs)						.89
< 70	330	61	1.13	0.68	1.86	
70+	49	17	1.05	0.39	2.82	
Primary tumor						.33
left/rectum	223	44	1.27	0.70	2.31	
right	145	30	0.81	0.40	1.67	
Differentiation						.14
well	75	16	0.47	0.16	1.37	
moderately	242	47	1.20	0.68	2.13	
poorly	45	9	4.43	0.90	21.78	
ECOG PS						.26
0	304	60	0.87	0.52	1.44	
1	68	15	1.68	0.57	4.92	
T-stage						.99
T3	243	42	1.04	0.57	1.91	
T4	136	36	1.03	0.53	1.99	
Perforation or obstruction						.87
no	289	58	1.06	0.63	1.78	
yes	90	20	1.15	0.48	2.77	
Vascular or perineural invasion						.33
no	299	60	0.94	0.56	1.56	
yes	80	18	1.56	0.61	4.03	
Number of examined nodes						.86
< 12	160	40	1.02	0.55	1.89	
12+	218	38	1.11	0.59	2.10	
BMI						.29
< 30	329	69	1.17	0.73	1.88	
30+	50	9	0.53	0.13	2.14	
**Overall**	**379**	**78**	**1.07**	**0.69**	**1.67**	

D

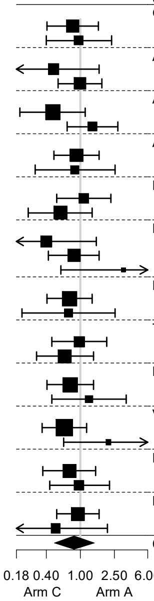

Covariate	N	Event	HR	LCI	UCI	P-value*
Gender						.78
male	226	35	0.82	0.41	1.61	
female	153	21	0.96	0.40	2.30	
Age (cutoff = 50 yrs)						.30
< 50	121	12	0.49	0.15	1.64	
50+	258	44	0.99	0.55	1.78	
Age (cutoff = 60 yrs)						.06
< 60	231	23	0.48	0.20	1.14	
60+	148	33	1.38	0.70	2.75	
Age (cutoff = 70 yrs)						.93
< 70	330	41	0.90	0.49	1.66	
70+	49	15	0.87	0.30	2.52	
Primary tumor						.24
left/rectum	223	29	1.09	0.53	2.27	
right	145	23	0.59	0.25	1.36	
Differentiation						.33
well	75	11	0.40	0.11	1.53	
moderately	242	35	0.84	0.43	1.64	
poorly	45	7	3.15	0.59	16.73	
ECOG PS						.98
0	304	43	0.75	0.41	1.37	
1	68	10	0.73	0.21	2.54	
T-stage						.48
T3	243	29	0.97	0.47	2.02	
T4	136	27	0.66	0.31	1.43	
Perforation or obstruction						.39
no	289	40	0.76	0.41	1.42	
yes	90	16	1.26	0.47	3.38	
Vascular or perineural invasion						.08
no	299	44	0.65	0.36	1.19	
yes	80	12	2.11	0.64	7.02	
Number of examined nodes						.67
< 12	160	32	0.75	0.37	1.51	
12+	218	24	0.97	0.44	2.17	
BMI						.41
< 30	329	47	0.94	0.53	1.66	
30+	50	9	0.52	0.13	2.08	
**Overall**	**379**	**56**	**0.85**	**0.50**	**1.44**	

HR, hazard ration; LCI, UCI, Lower and Upper 95% Confidence Interval; ECOG, Eastern Cooperative Oncology Group; PS, performance status; BMI, body mass index

*: *P*-value for the interaction test between the subgroup and treatment arm

© 2020 Chibaudel B et al. *JAMA Network Open.*
