# Supplementary material for: Association of Bevacizumab Plus Oxaliplatin-Based Chemotherapy With Disease-Free Survival and Overall Survival in Patients With Stage II Colon Cancer: A Secondary Analysis of the AVANT Trial
Source: JAMA Netw Open. 2020 Oct 19;3(10):e2020425. doi: 10.1001/jamanetworkopen.2020.20425 (PMC7573695; doi:10.1001/jamanetworkopen.2020.20425)
Supplement: Supplement 3. — Data Sharing Statement [file jamanetwopen-e2020425-s003.pdf]

Data Sharing Statement

Chibaudel. Association of Bevacizumab Plus Oxaliplatin-Based Chemotherapy With Disease-Free Survival and Overall Survival in Patients With Stage II Colon Cancer. *JAMA Netw Open*. Published October 19, 2020. 10.1001/jamanetworkopen.2020.20425

Data
Data available: No
